# Supplementary material for: Virtual fragment screening for DNA repair inhibitors in vast chemical space
Source: Nat Commun. 2025 Feb 18;16:1741. doi: 10.1038/s41467-025-56893-9 (PMC11836371; doi:10.1038/s41467-025-56893-9)
Supplement: Supplementary file 1 — Supplementary Information [file 41467_2025_56893_MOESM1_ESM.pdf]

Supporting Information:

Virtual Fragment Screening for DNA Repair Inhibitors in Vast Chemical Space

Andreas Luttens[1,2,3], Duc Duy Vo[1], Emma R. Scaletti[4], Elisée Wiita[5], Ingrid Almlöf[5], Olov Wallner[5], Jonathan Davies[4], Sara Košenina[4], Liuzhen Meng[5], Maeve Long[5], Oliver Mortusewicz[5], Geoffrey Masuyer[4], Flavio Ballante[1], Maurice Michel[5], Evert Homan[5], Martin Scobie[5], Christina Kalderén[5], Ulrika Warpman Berglund[5], Andrii V. Tarnovskiy[6], Dmytro S. Radchenko[6], Yurii. S. Moroz[6,7,8], Jan Kihlberg[9], Pål Stenmark[4], Thomas Helleday[5,10], Jens Carlsson[1,*]

[1]Science for Life Laboratory, Department of Cell and Molecular Biology, Uppsala University, BMC, Box 596, SE-751 24 Uppsala, Sweden

[2]Institute for Medical Engineering & Science and Department of Biological Engineering, Massachusetts Institute of Technology, Cambridge, MA 02139, USA

[3]Infectious Disease and Microbiome Program, Broad Institute of MIT and Harvard, Cambridge, MA 02142, USA

[4]Department of Biochemistry and Biophysics, Stockholm University, SE-106 91 Stockholm, Sweden

[5]Science for Life Laboratory, Department of Oncology-Pathology, Karolinska Institute, SE-171 77 Stockholm, Sweden

[6]Enamine Ltd., 02094 Kyiv, Ukraine

[7]Taras Shevchenko National University of Kyiv, Kyiv 01601, Ukraine

[8]Chemspace LLC, Kyiv 02094, Ukraine

[9]Department of Chemistry-BMC, Uppsala University, SE-751 23 Uppsala, Sweden

[10]Sheffield Cancer Centre, Department of Oncology and Metabolism, University of Sheffield, Sheffield, United Kingdom

[*]To whom correspondence should be addressed: jens.carlsson@icm.uu.se

Table of Contents

Page

Supplementary Tables

Table S1a. Summary of selected fragments from the docking screen. S3
Table S1b. Summary of selected lead-like compounds from the docking screen. S5
Table S2. Virtual fragment screening hits S8
Table S3. Crystallographic data collection and refinement statistics S9
Table S4. Fragment elaborations in commercial chemical space. S12
Table S5. Fragment elaborations in tailored chemical space. S17
Table S6. Physicochemical and *in vitro* ADME properties of OGG1 inhibitors. S20
Table S7. Size comparison between GDB17 and UniverseGenerator chemical spaces. S21
Table S8. Virtual fragment screening for three diverse drug targets. S22
Table S9. Protein preparation for molecular docking (SMYD3, NUDT5, PHIP). S23

Supplementary Figures

Figure S1. Evaluation of the virtual screening performance for OGG1 crystal structure. S24
Figure S2. mOGG1 crystal structures with electron density difference and omit maps S25
Figure S3. Workflow for the generation of the tailored virtual libraries. S26
Figure S4. Workflow for generation of the substituent space. S27
Figure S5. Example of scaffold activation. S28
Figure S6. Example of superstructure generation. S29

Supplementary Methods

Synthesis Procedures S30
LCMS Spectral Data S40
NMR Spectra S58

Supplementary References S74

Supplementary Tables

Supplementary Table S1a. Top-ranked compounds selected from the fragment docking screen. Thermal shift assay was carried out at a concentration of 495 μM.

Structure	Identifier[a]	Score (kcal/mol)	Cluster rank	Global rank	ΔT_m (K)
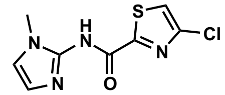	ZINC000851710058	-44.70	26	45	-0.05
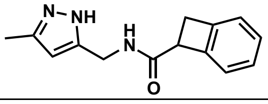	ZINC000088452370	-43.78	45	88	0.26
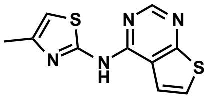	ZINC000163077816	-43.61	49	103	-0.51
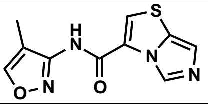	ZINC000772563154	-43.54	56	112	0.52
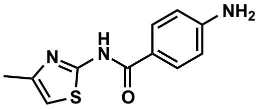	ZINC000005740683[b]	-43.46	63	124	0.52
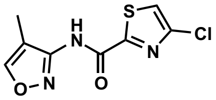	ZINC000789997344	-43.19	77	156	-0.03
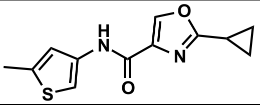	ZINC000634423932	-43.13	86	169	-0.08
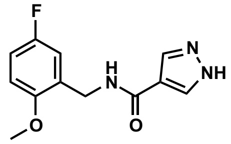	ZINC000188683358	-42.85	105	217	-0.01
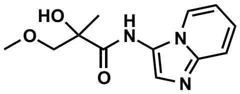	ZINC000618358369	-42.73	116	238	0.11
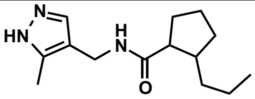	ZINC000294547394	-42.66	125	250	0.18
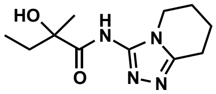	ZINC000935297684	-42.63	127	257	-0.15
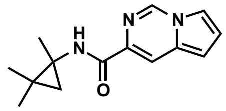	ZINC000825682784[b] compound **2**	-42.36	147	311	1.6
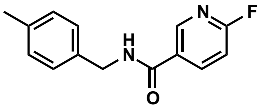	ZINC000069704260[b] compound **3**	-42.10	181	391	0.6
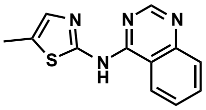	ZINC000031949200	-41.86	219	480	-0.03

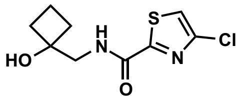	ZINC000789956517	-41.80	231	502	0.07
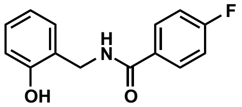	ZINC000082283111[b]	-41.72	242	535	0.67
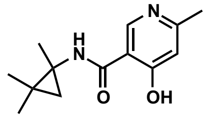	ZINC000620078540	-41.63	258	570	0.03
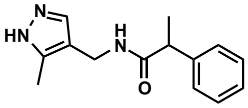	ZINC000182379077	-41.61	263	581	0.08
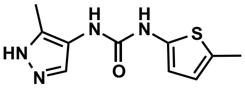	ZINC000075413056	-41.57	278	619	0.35
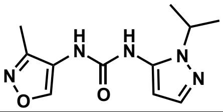	ZINC000866108350	-41.48	296	658	-0.03
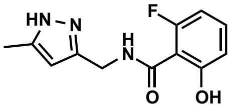	ZINC000681276767	-41.43	313	693	0.01
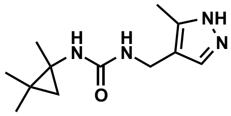	ZINC000663691150	-41.25	350	802	0.01
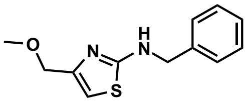	ZINC000196610182	-41.21	369	850	-0.06
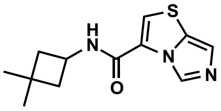	ZINC000856336878[b]<br>compound **4**	-41.20	371	854	0.5
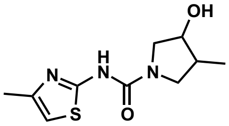	ZINC000350512979	-41.12	397	914	0.07
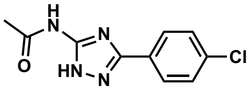	ZINC000517746346	-41.06	418	965	-0.15
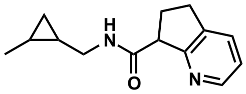	ZINC000341791847	-41.05	420	970	0.38
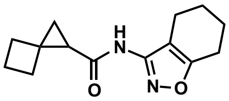	ZINC000900780107[b]<br>compound **1**	-40.89	464	1104	2.8
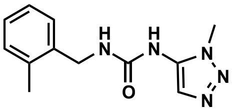	ZINC000787791820	-40.84	482	1150	-0.02

[a] ZINC database identifier. [b] Protein crystallography was attempted for these compounds. Source data are provided as a Source Data file.

Supplementary Table S1b. Top-ranked compounds selected from the lead-like docking screen. Thermal shift assay was carried out at a concentration of 99 μM.

Structure	Identifier[a]	Score (kcal/mol)	Cluster rank	Global rank	ΔT_m (K)
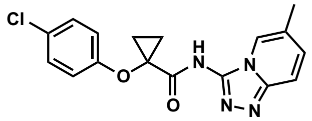	ZINC000341519123	-50.43	54	713	0.37
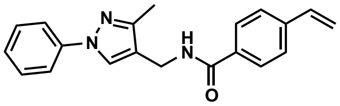	ZINC000192206237	-49.21	131	1708	0.83
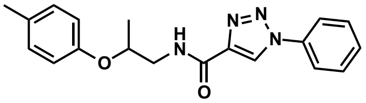	ZINC000080662795	-48.92	164	2061	-0.11
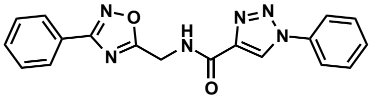	ZINC000046356107	-48.50	225	2705	-0.26
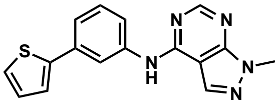	ZINC000507023994	-48.19	253	3276	-0.92
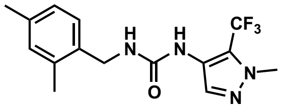	ZINC000445022399	-47.95	286	3820	-0.23
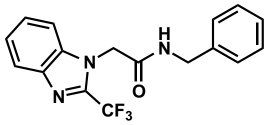	ZINC000000241283	-47.53	367	4901	0.39
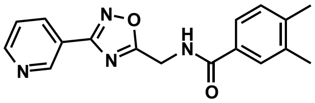	ZINC000032814728	-47.45	389	5124	0.79
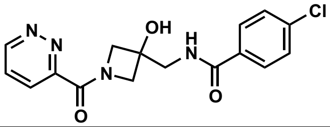	ZINC001046413262	-47.38	402	5341	-0.07
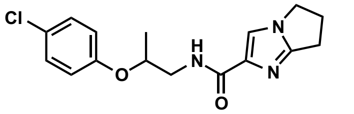	ZINC000584413720	-47.26	305	5726	0.65
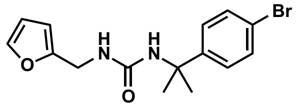	ZINC000047544921	-47.14	469	6153	0.05
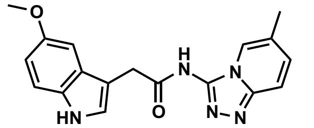	ZINC000088254610	-47.11	481	6251	0.27
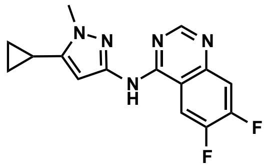	ZINC000520908378	-46.93	545	6920	-0.38

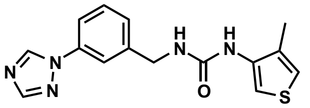	ZINC000439491208	-46.62	661	8276	-0.04
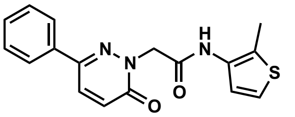	ZINC001624168456	-46.52	693	8750	0.15
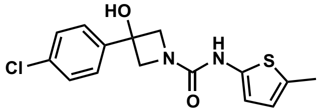	ZINC000776423770	-46.40	739	9360	-0.42
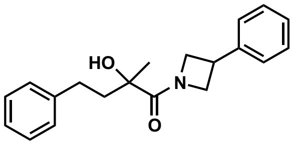	ZINC000633809751	-46.36	760	9562	0.48
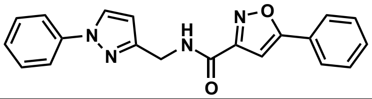	ZINC000342133714	-46.24	822	10283	0.08
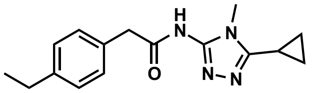	ZINC000617287579	-46.14	874	10892	-0.19
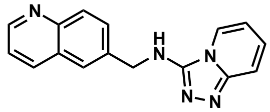	ZINC000157183023	-45.96	970	12002	Inactive[b]
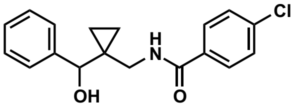	ZINC000155922887	-45.94	983	12093	inactive[b]
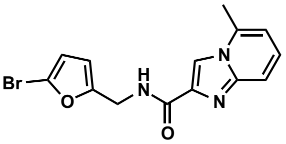	ZINC000700852234	-45.79	1073	13110	-0.27
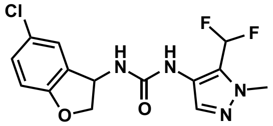	ZINC000677630607	-45.70	1132	13776	-0.08
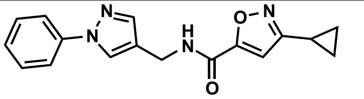	ZINC000337657385	-45.20	1486	17986	-0.16
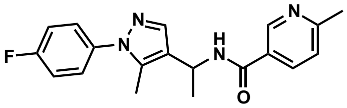	ZINC000045518705	-45.12	1543	18823	-0.07
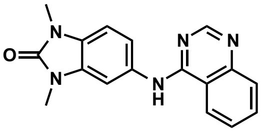	ZINC000142711832	-45.11	1545	18860	-0.11
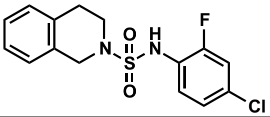	ZINC000358521717	-44.69	1944	23709	0.43

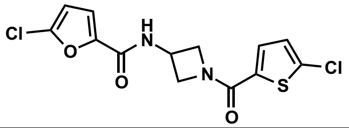	ZINC000990255105	-44.67	1969	23944	-0.03
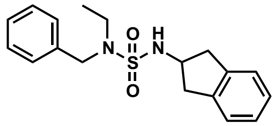	ZINC000357905855	-44.44	2258	27051	0.23
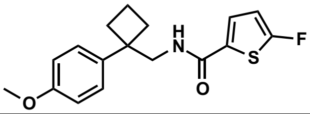	ZINC000613462643	-44.38	2318	27936	0.05
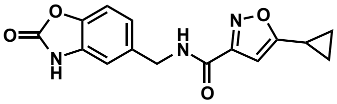	ZINC001146929060	-44.25	2492	29925	0.00
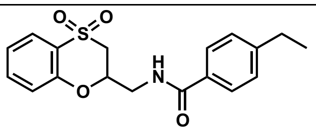	ZINC000572161337	-44.12	2662	31940	0.17
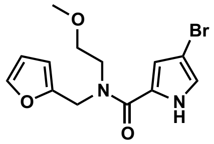	ZINC000025095824	-43.82	3120	37314	0.10
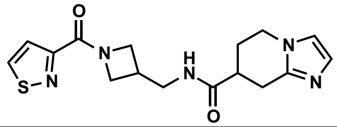	ZINC001002782586	-43.74	3263	38897	-0.13
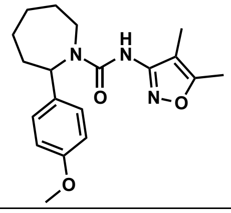	ZINC000455661485	-43.66	3421	40568	-0.14
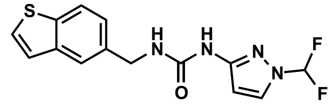	ZINC000350560229	-43.60	3517	41751	-0.01

[a] ZINC database identifier. [b] No curve could be obtained for these molecules. Source data are provided as a Source Data file.

Supplementary Table S2. Hits from the virtual fragment screen, inhibitory potencies, thermal shifts, and structures of complexes with OGG1.

Cmpd	Structure	pIC_{50}[a]	ΔT_m (K)[b]
**TH5487**[1,2]	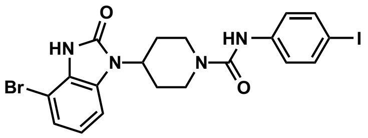	6.47[d]	4.3
**1**	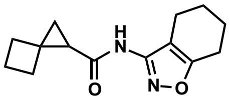	< 4	2.8
**2**	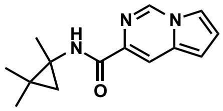	< 4	1.6
**3**	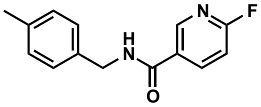	< 4	0.6
**4**	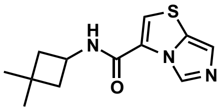	< 4	0.5

[a] pIC_{50} values from enzyme inhibition assay. [b] Thermal stabilization by differential scanning fluorimetry from four technical replicates at a concentration of 495 μM. Source data are provided as a Source Data file.

Supplementary Table S3. Data collection and refinement statistics from crystallographic experiments.

Complex identifier	mOGG1-TH011247	mOGG1-TH011228	mOGG1-TH11227
Ligand identifier	Compound **1**	Compound **2**	Compound **3**
**Data collection**			
PDB code	7QEL	7ZG3	8CEX
Station	DLS-I24	DLS-I24	DLS-I04
Space group	$P2_12_12_1$	$P2_12_12_1$	$P2_12_12_1$
Cell dimensions:			
a, b, c (Å)	81.4, 81.4, 168.4	80.8, 81.3, 169.9	81.1, 81.3, 170.2
α, β, γ (°)	90.0, 90.0, 90.0	90.0, 90.0, 90.0	90.0, 90.0, 90.0
Resolution (Å)	40.7-2.50 (2.60-2.50)	84.9-2.30 (2.38-2.30)	58.8-2.30 (2.38-2.30)
Total reflections	445719 (49570)	1222331 (113233)	660160 (62309)
Unique reflections	39517 (4392)	50573 (4564)	50891 (4601)
R_{merge}	0.171 (2.591)	0.171 (3.859)	0.139 (2.056)
R_{pim}	0.075 (1.124)	0.050 (1.119)	0.057 (0.837)
$CC_{1/2}$	0.996 (0.637)	0.990 (0.540)	0.998 (0.543)
I/σ	10.5 (2.0)	12.0 (0.9)	9.9 (1.3)
Completeness	100 (100)	100 (100)	100 (100)
Redundancy	11.3 (11.3)	24.2 (24.8)	13.0 (13.5)
**Refinement**			
R_{work}/R_{free} (%)	25.2/29.5	27.6/30.7	24.2/27.8
B-factors:			
Protein [a]	47.2/70.0/71.1	77.5/74.9/55.1	51.5/77.7/77.7
Ligand [a]	51.3/73.9/80.3	74.2/75.9/50.8	43.9
Water	49.1	56.4	49.5
R.m.s. deviations:			
Bond lengths (Å)	0.002	0.007	0.008
Bond angles (°)	1.18	0.944	1.40
Ramachandran statistics:			
Favoured (%)	94.4	96.4	95.5
Allowed (%)	4.8	3.2	0.1
Outliers (%)	0.8	0.4	0.4

(**Supplementary Table S3** Continued.)

Complex identifier	mOGG1-TH11233	mOGG1-TH012035	mOGG1-TH012941
Ligand identifier	Compound **4**	Compound **5**	Compound **7**
**Data collection**			
PDB code	8CEY	7Z5R	7ZC7
Station	DLS-I04	DESY PETRA3-P13	DLS-I03
Space group	$P2_12_12_1$	$P2_12_12_1$	$P2_12_12_1$
Cell dimensions:			
a, b, c (Å)	81.6, 81.7, 170.4	81.5, 81.5, 170.1	80.9, 81.7, 168.2
α, β, γ (°)	90.0, 90.0, 90.0	90.0, 90.0, 90.0	90.0, 90.0, 90.0
Resolution (Å)	59.0-1.95 (1.99-1.95)	170.2-2.50 (2.60-2.50)	168.2-2.30 (2.38-2.30)
Total reflections	1146762 (63762)	506588 (58073)	663622 (62335)
Unique reflections	83828 (4543)	40059 (4457)	50390 (4567)
R_{merge}	0.102 (4.975)	0.165 (2.508)	0.083 (2.183)
R_{pim}	0.041 (1.981)	0.069 (1.037)	0.034 (0.879)
$CC_{1/2}$	0.999 (0.372)	0.997 (0.586)	0.999 (0.635)
I/σ	14.1 (0.6)	8.8 (1.3)	17.3 (1.0)
Completeness	99.9 (99.9)	100 (100)	100 (100)
Redundancy	13.7 (14.0)	12.6 (13.0)	13.2 (13.6)
**Refinement**			
R_{work}/R_{free} (%)	24.4/28.2	23.7/29.4	23.9/30.0
B-factors:			
Protein [a]	45.5/65.2/54.7	54.8/87.1/75.7	74.3/76.7/56.2
Ligand [a]	44.6/49.5/64.6	61.3/85.2/82.1	68.5/62.7/n.a
Waters	48.3	54.8	55.2
R.m.s. deviations:			
Bond lengths (Å)	0.007	0.009	0.014
Bond angles (°)	1.34	1.24	1.60
Ramachandran statistics:			
Favoured (%)	96.81	95.2	94.5
Allowed (%)	3.19	4.3	4.7
Outliers (%)	0.0	0.5	0.9

(**Supplementary Table S3** Continued.)

Complex identifier	mOGG1-TH013545	mOGG1-TH013546
Ligand identifier	Compound **8**	Compound **17**
**Data collection**		
PDB code	7Z3Y	7Z5B
Station	MAXIV-BioMAX	DLS-I03
Space group	$P2_12_12_1$	$P2_12_12_1$
Cell dimensions:		
a, b, c (Å)	80.1, 81.8, 168.9	80.5, 80.6, 165.9
α, β, γ (°)	90.0, 90.0, 90.0	90.0, 90.0, 90.0
Resolution (Å)	73.0-2.35 (2.43-2.35)	57.8-2.60 (2.72-2.60)
Total reflections	632790 (61979)	392023 (52743)
Unique reflections	47609 (4580)	33992 (4084)
R_{merge}	0.098 (3.316)	0.179 (3.572)
R_{pim}	0.040 (1.340)	0.079 (1.492)
$CC_{1/2}$	0.999 (0.562)	0.994 (0.996)
I/σ	12.8 (1.0)	9.0 (1.0)
Completeness	100 (100)	100 (100)
Redundancy	13.3 (13.5)	11.5 (12.9)
**Refinement**		
R_{work}/R_{free} (%)	25.9/31.0	25.9/30.3
B-factors:		
Protein [a]	65.2/87.6/95.0	70.9/81.2/80.2
Ligand [a]	85.6/n.a/n.a	70.6/82.5/72.4
Waters	68.2	60.6
R.m.s. deviations:		
Bond lengths (Å)	0.002	0.007
Bond angles (°)	1.16	1.01
Ramachandran statistics:		
Favoured (%)	95.7	96.4
Allowed (%)	4.3	3.2
Outliers (%)	0	0.3

Values in parentheses are for the highest-resolution shell. [a] Values for each monomer (A, B and C) of the asymmetric unit. In the crystal structure of compound **8** the ligand is only bound in monomer A. In the crystal structure of compound **7** the ligand is only bound in monomers A and B.

Supplementary Table S4. Fragment elaborations from commercial chemical libraries and inhibitory potencies.

Structure	Identifier[a]	IC_{50} (μM)
**Optimization Round 1**		
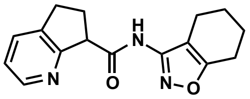	ZINC900786926	>99
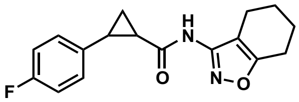	ZINC900776931	>99
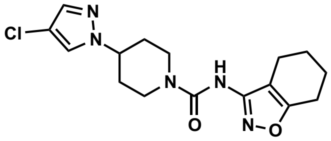	ZINC1333476568	>99
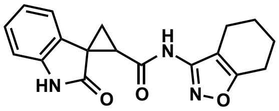	ZINC900789538	>99
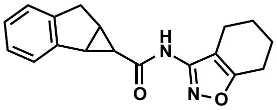	ZINC900785889	>99
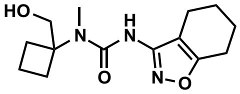	ZINC934349345	>99
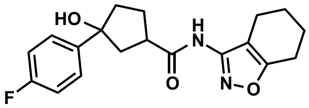	ZINC934354817	>99
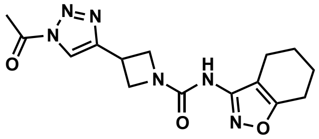	ZINC1467519463	>99
**Optimization Round 2**		
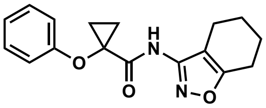	ZINC1339068026	>99
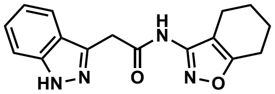	ZINC900776257	21
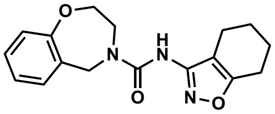	ZINC864252211	>99
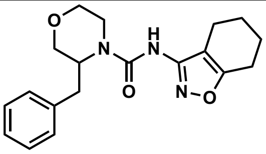	ZINC934346451	>99

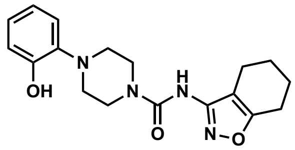	ZINC934354679	>99
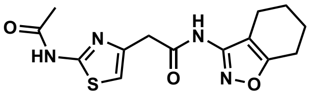	ZINC900776225	>99
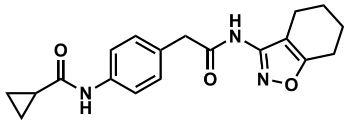	ZINC900779120	>99
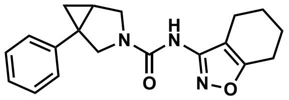	ZINC934343692	>99
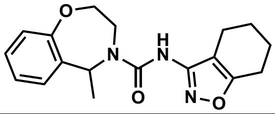	ZINC934350377	>99
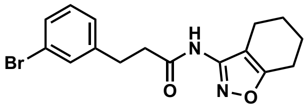	ZINC1540181889	>99
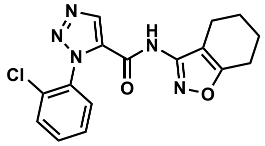	ZINC1339066206	>99
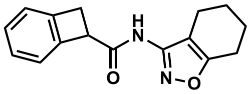	ZINC900781082<br>compound **5**	58
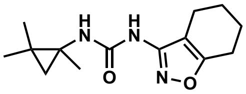	ZINC934346704	>99
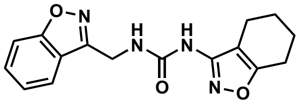	ZINC934345618	>99
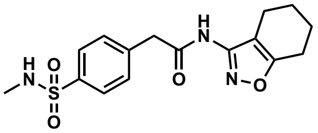	ZINC1364121893	>99
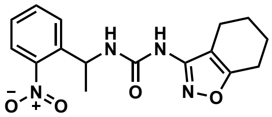	ZINC1311315433	>99
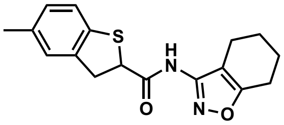	ZINC1875406025	>99
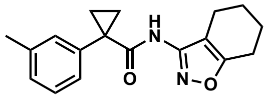	ZINC1337721906	>99

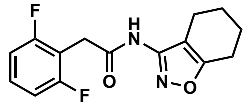	ZINC900780900<br>compound **6**	36
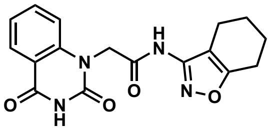	ZINC1472632088	>99
**Optimization Round 3**		
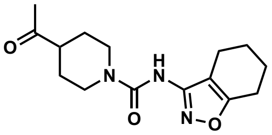	ZINC934348809	>99
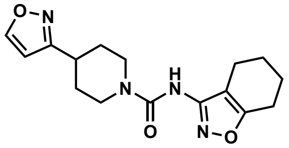	ZINC934345542	>99
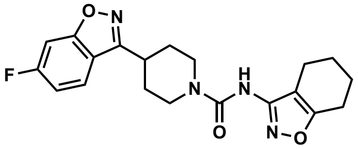	ZINC934352132	>99
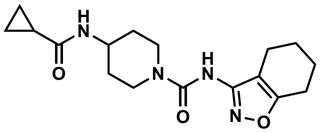	ZINC934353088	>99
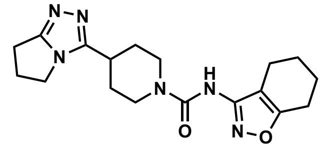	ZINC934358969	>99
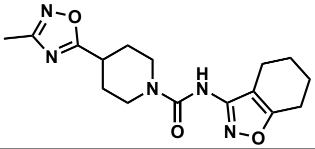	ZINC934362357	>99
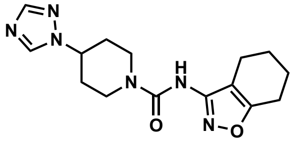	ZINC1875271616	>99
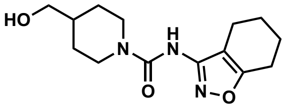	ZINC934361799	>99
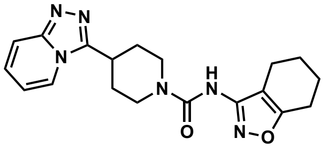	ZINC934363767	>99

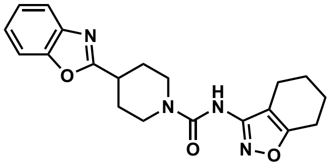	ZINC1355080415	>99
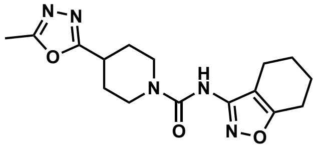	ZINC1875296543	>99
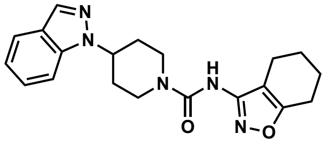	ZINC1875296755	>99
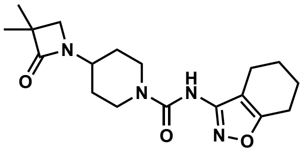	ZINC1875374552	>99
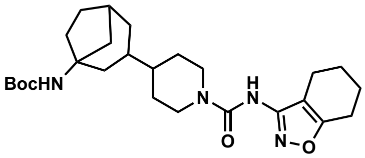	Z4327164893	>99
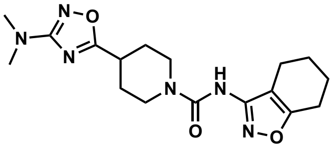	ZINC1875323528	>99
**Optimization Round 4**		
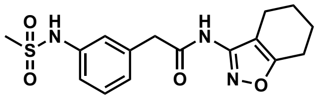	ZINC1364134940	>99
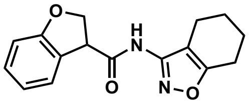	ZINC900780699	>99
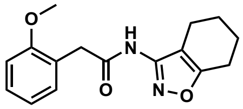	ZINC900773561	>99
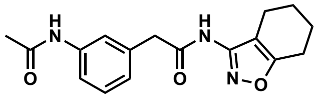	ZINC900787359	>99
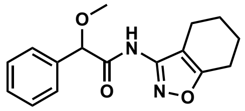	ZINC1650248873	>99
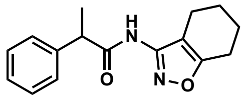	ZINC900779065	>99

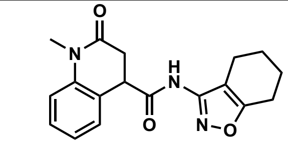	ZINC2087899171	>99
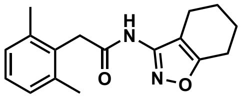	Z2987483969	>99
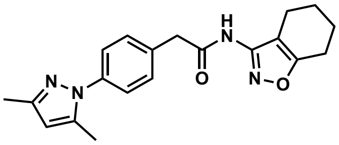	ZINC1134903408<br>compound **7**	6.6
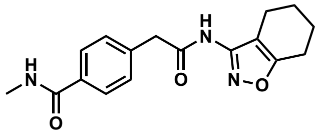	ZINC2087902149	>99
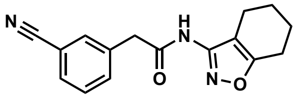	ZINC900779624	>99
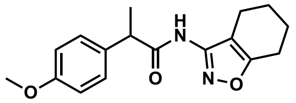	ZINC900779149	>99
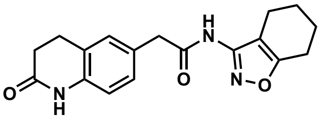	ZINC2087901924	>99
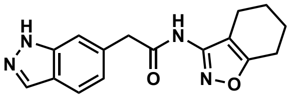	ZINC900780314	>99
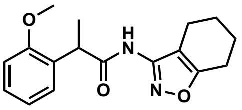	Z3950301447	>99
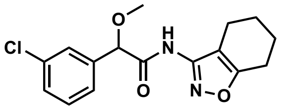	Z4141434163	>99
**Optimization Round 5**		
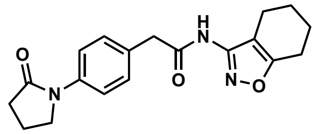	ZINC2325764888	>99
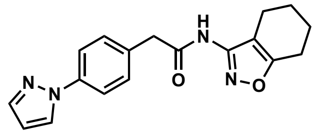	ZINC900777273	>99
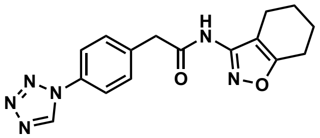	ZINC900776621	>99

[a] ZINC database (ZINC*) or Enamine (Z*) identifier. Source data are provided as a Source Data file.

Supplementary Table S5. Fragment elaborations from in-house synthesis: Inhibitory potencies, NFκB inhibition, and thermal stabilization.

Cmpd	Structure	pIC_{50} (M)[a]	pEC_{50} (M)[b]	ΔT_m (K)[c]
**7**	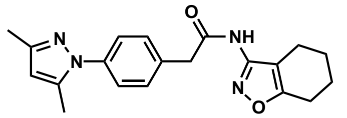	5.18 ± 0.05 (n=5)	4.45 ± 0.15 (n=3)	5.5 ± 1.2 (n=4)
**8**	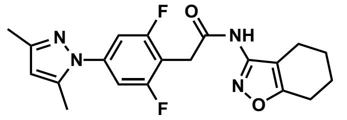	5.19 ± 0.08 (n=3)	4.95 ± 0.12 (n=2)	5.4 ± 0.5 (n=3)
**9 (Mixture of regio-isomers)**	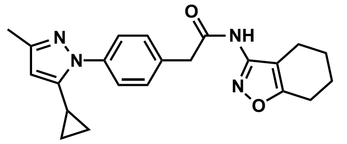	5.43 ± 0.00 (n=2)	5.04 ± 0.10 (n=3)	6.6 ± 0.6 (n=3)
**10**	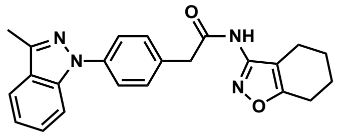	5.89 ± 0.06 (n=3)	5.18 ± 0.18 (n=3)	4.8 ± 0.4 (n=3)
**11**	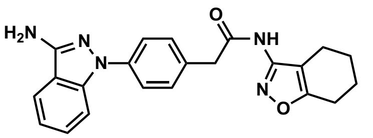	5.69 ± 0.08 (n=3)	5.00 ± 0.02 (n=3)	4.7 (n=1)

**12**	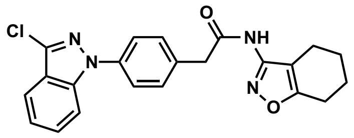	4.98 ± 0.95<br>(n=2)	5.26<br>(n=1)	2.3<br>(n=1)
**13**	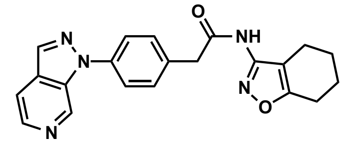	4.32 ± 0.02<br>(n=2)	4.85<br>(n=1)	not<br>determined
**14**	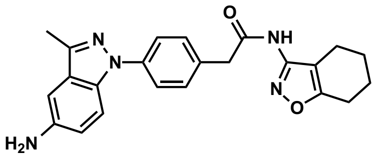	5.01 ± 0.07<br>(n=3)	5.18<br>(n=1)	not<br>determined
**15**	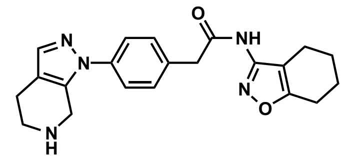	< 4<br>(n=2)	4.88<br>(n=1)	0.3<br>(n=1)
**16**	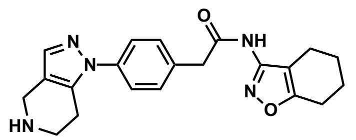	< 4<br>(n=1)	< 4.5<br>(n=1)	not<br>determined
**17**	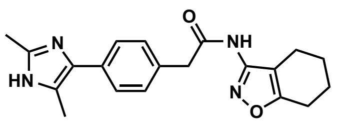	6.22 ± 0.13<br>(n=9)	4.72 ± 0.18<br>(n=3)	8.1 ± 0.1<br>(n=3)

**18**	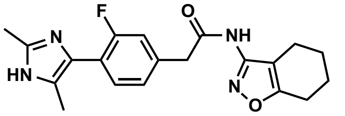	6.08 ± 0.07 (n=3)	4.93 ± 0.08 (n=3)	7.8 (n=1)
**19**	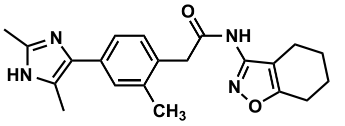	6.09 ± 0.05 (n=3)	4.98 ± 0.30 (n=3)	7.4 ± 0.2 (n=3)
**20**	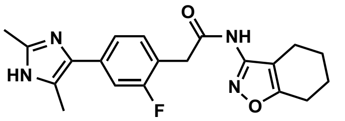	6.13 ± 0.07 (n=3)	4.69 ± 0.12 (n=3)	8.6 ± 0.5 (n=3)
**21**	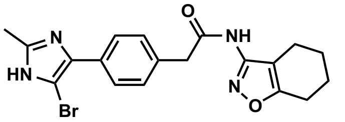	5.68 ± 0.14 (n=3)	4.94 ± 0.09 (n=3)	5.9 (n=1)
**22**	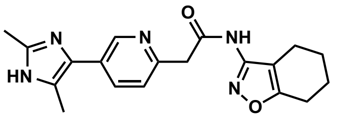	5.71 ± 0.12 (n=3)	4.55 ± 0.02 (n=2)	6.3 (n=1)
**23**	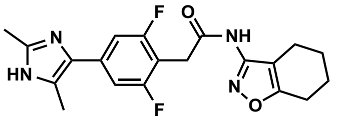	6.22 ± 0.10 (n=3)	5.20 ± 0.11 (n=3)	9.0 ± 0.2 (n=3)

[a] pIC_{50} values ± SD from enzyme inhibition assay. [b] pEC_{50} values ± SD from NFκB activation assay. [c] Thermal shift values ± SD from DSF experiments at a concentration of 99 μM. Source data are provided as a Source Data file.

Supplementary Table S6. Physicochemical and *in vitro* ADME properties of OGG1 inhibitors.

Property	TH5487	Compound **17**	Compound **23**
pIC_{50} (μM)	6.47	6.22	6.22
Number of heavy atoms	27	26	28
Molecular weight (g/mol)	541.2	350.4	386.4
clogP	4.6	3.7	4.0
LE (kcal/mol/HA)[a]	0.33	0.33	0.31
LLE[b]	1.87	2.52	2.22
In vitro ADME properties[c]			
Thermodynamic solubility (μmol/L)	< 1	667	16
CL_{int} (μL/min/mg)[d]	4.6	23.9	39.7
Protein plasma binding, fu (%)[e]	0.1	1.4	4.8
P_{app} AB (10^{-6} cm/s)[f]	4.16	19.82	6.13
P_{app} BA (10^{-6} cm/s)[f]	4.83	133.78	94.19
Efflux ratio[f]	1.16	6.7	15.4

[a] Ligand efficiency (LE), calculated as: LE = –RTLn(IC_{50})/HA, HA = Number of ligand heavy atoms. [b] Lipophilicity Ligand Efficiency, calculated as: LLE = pIC_{50} – cLogP. [c] Experiments carried out in a single replicate. [d] Intrinsic clearance (CL_{int}) in human liver microsomes. [e] Fraction unbound (fu) in human plasma. [f] Results from Caco-2 cell permeability assays. Source data are provided as a Source Data file.

Supplementary Table S7. Size comparison between GDB17 and UniverseGenerator chemical spaces. Differences can be attributed to a combination of different toolkit library versions, handling and standardization of tautomers, and the chemical perception of specific functional groups, such as bridgehead atoms. Source data are provided as a Source Data file.

Heavy Atom Count	GDB17	UniverseGenerator
1	3	3
2	6	6
3	14	14
4	47	49
5	219	228
6	1091	1244
7	6029	7260
8	37435	46001
9	243233	262914
10	1670163	2297138
11	12219460	18486152

Supplementary Table S8. Virtual fragment screening statistics for three diverse drug targets.

Protein target	SMYD3	NUDT5	PHIP
Fragment docking score (kcal/mol)	–48.89	–48.85	–33.71
	**Random molecules**		
Successfully docked	9756 (100%)	974743 (100%)	121644 (100%)
Improved docking score [a]	9 (0.1%)	73888 (7.6%)	6997 (5.8%)
	**Substructure search: Unconstrained SMARTS pattern**		
Superstructures	9914 (100%)	737407 (100%)	145400 (100%)
Ready to dock format	9907 (99.9%)	735998 (99.8%)	124059 (85.7%)
Successfully docked	9756 (98.4%)	715311 (97.0%)	121644 (83.7%)
Improved docking score[a]	836 (8.4%)	57523 (7.8%)	58955 (40.5%)
RMSD < 2 Å[b]	3016 (30.4%)	3109 (0.4%)	73497 (50.5%)
Improved docking score and RMSD < 2 Å[c]	814 (8.2%)	1856 (0.3%)	45679 (31.4%)
Fold improvement over random molecules [d]	84.0	1.0	7.0
	**Substructure search: Constrained SMARTS pattern[e]**		
Superstructures	6600 (100%)	5051 (100%)	133568 (100%)
Ready to dock format	6594 (99.9%)	4693 (92.9%)	113197 (84.7%)
Successfully docked	6516 (98.7%)	4645 (92.0%)	111872 (83.8%)
Improved docking score	826 (12.5%)	1962 (38.8%)	57218 (42.8%)
RMSD < 2 Å	2808 (42.5%)	3109 (61.6%)	67729 (50.7%)
Improved docking score and RMSD < 2 Å	807 (12.2%)	1856 (36.7%)	44433 (33.3%)
Fold improvement over random molecules	125.0	5.6	7.4

[a] The number of compounds with an improved docking score compared to the bound fragment. [b] The number of compounds with a common-heavy-atom RMSD value < 2 Å to the bound fragment. [c] The number of compounds with a common-heavy-atom RMSD value <2 Å to the bound fragment *and* improved docking score compared to the bound fragment. [d] Ratio of fraction of superstructures with docking scores better than the initial fragment and fraction of random molecules with better docking scores than the initial fragment. [e]SMARTS pattern in which growing vectors corresponding to key hydrogen bond donors observed in the crystal structured are occluded. Source data are provided as a Source Data file.

Supplementary Table S9. Protein preparation for molecular docking (SMYD3, NUDT5, PHIP).

Target	Template[a]	Polarized residues[b]	Histidine protonation states	Matching spheres	Electrostatic radius[c]	Desolvation radius
SMYD3	5CCL[3]	T184, S202, Y239	δ: 83, 153, 220, 199<br>ε: 206, 366, 382, 404, 408	45	default	0.0 Å
NUDT5	5QJK[4]	E47	δ: 142, 206<br>ε: 73, 190	45	1.0 Å	0.2 Å
PHIP	5RKI[5]	P1340, S1392	δ: 1417, 1432	45	1.2 Å	0.2 Å

[a] PDB accession code. [b] Increase of dipole moments by adding partial charges to atoms, without altering the total charge of the residue. [c] Tangent thin sphere radius. Default refers to low dielectric spheres generated by the DOCK3.7/blastermaster SPHGEN program prior to thin sphere protocols.

Supplementary Figures

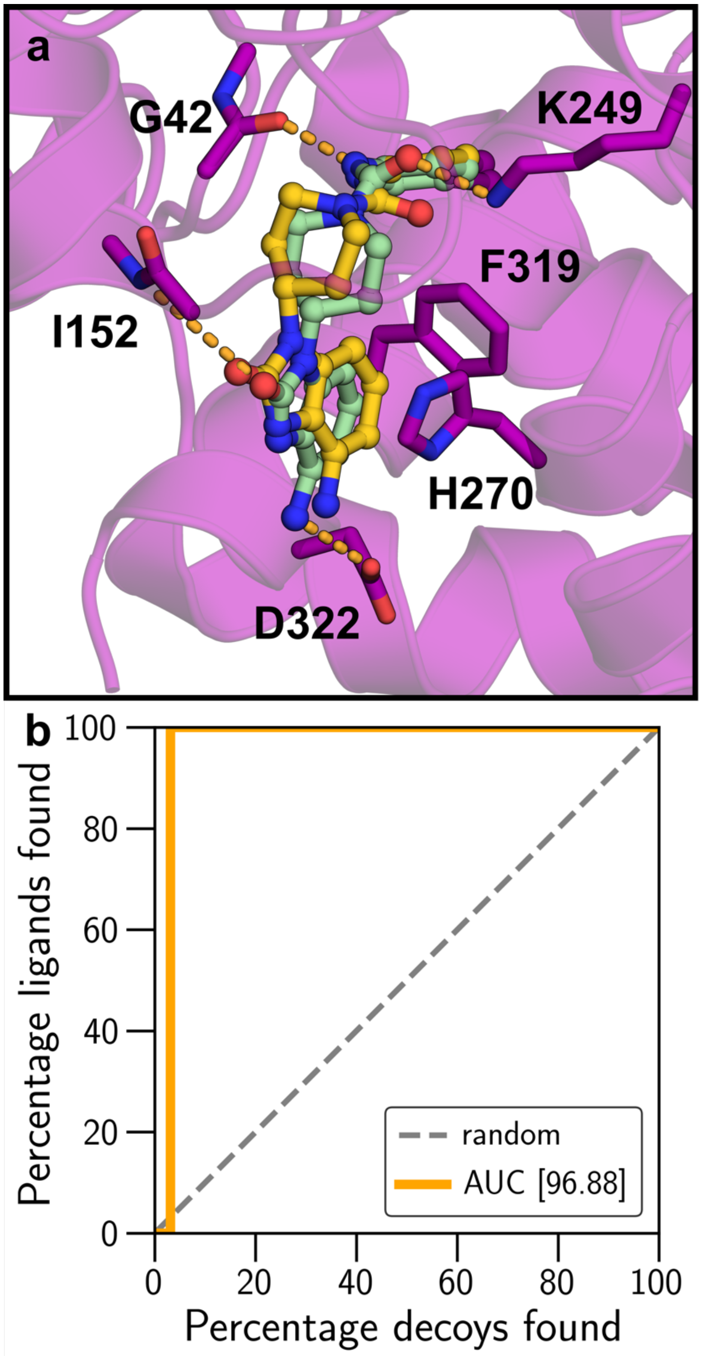

Supplementary Figure S1. Evaluation of the virtual screening performance for the OGG1 crystal structure. (a) Redocking of **TH5675** into the active site resulted in an accurate binding mode (symmetry-corrected heavy atom RMSD = 0.77 Å) and successfully captured the key interactions between OGG1 and the inhibitor. The protein (PDB accession code: 6G3Y) is shown as a purple cartoon. The experimental and predicted inhibitor binding modes are shown as golden and green sticks, respectively. Selected side chains are shown as sticks, and hydrogen bonds are shown as yellow dashed lines. (b) Receiver operator characteristic (ROC) curve describing the enrichment of OGG1 inhibitors (**TH5675** scaffold) over property-matched decoys. Docking to the OGG1 crystal structure strongly enriched the inhibitors. Source data are provided as a Source Data file.

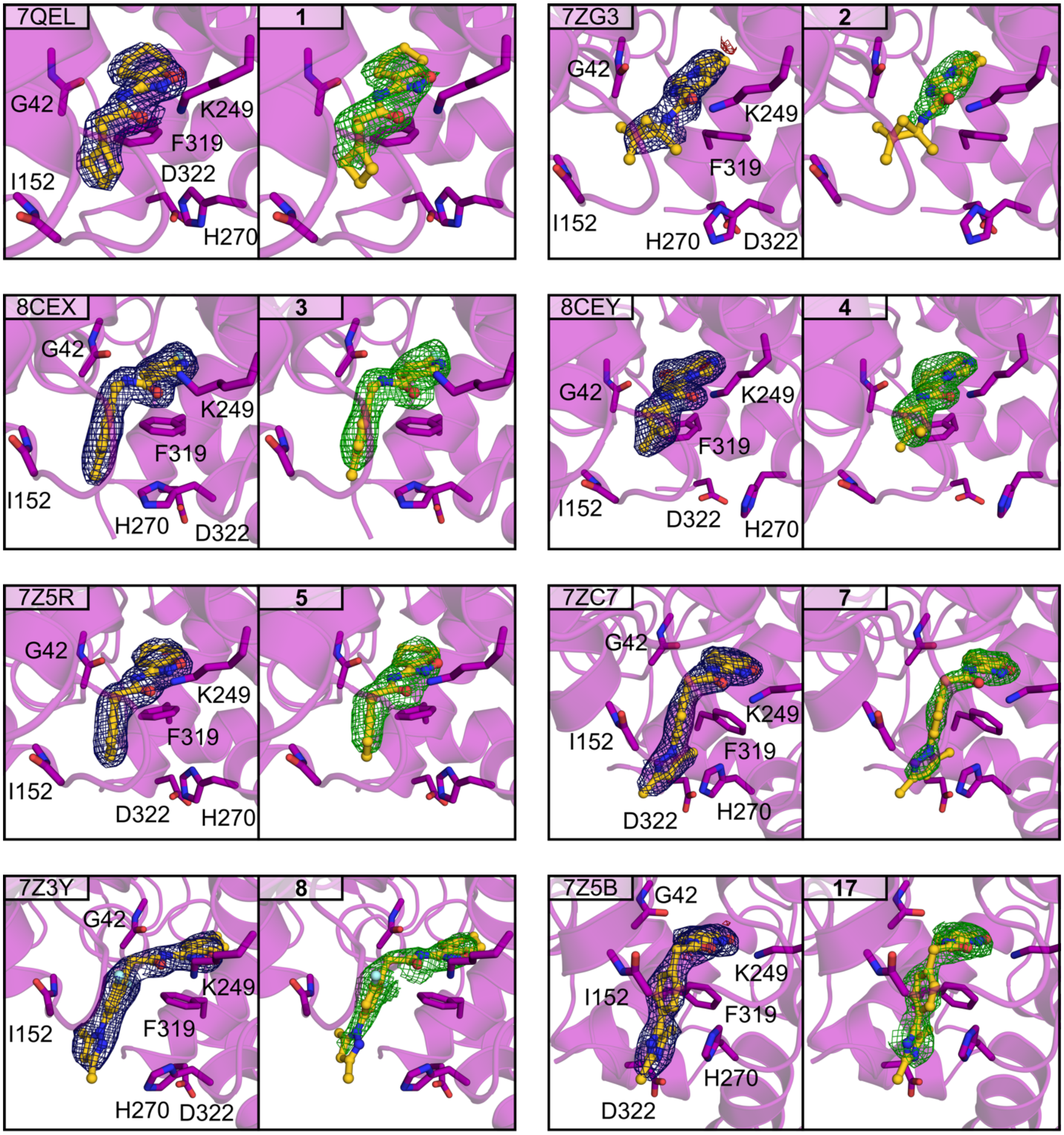

Supplementary Figure S2. mOGG1 crystal structures with electron density difference and omit maps. For each compound, the 2Fo-Fc map is represented by a blue isomesh at +1σ, the Fo-Fc map is +3σ and -3σ are represented by green and red isomeshes respectively (left). The Fo-Fc omit map is represented by a green isomesh at +3σ (right). Maps are carved at 1.5 Å from the ligand. The proteins are drawn as purple cartoons and selected amino acid side chains and the ligands are shown as sticks.

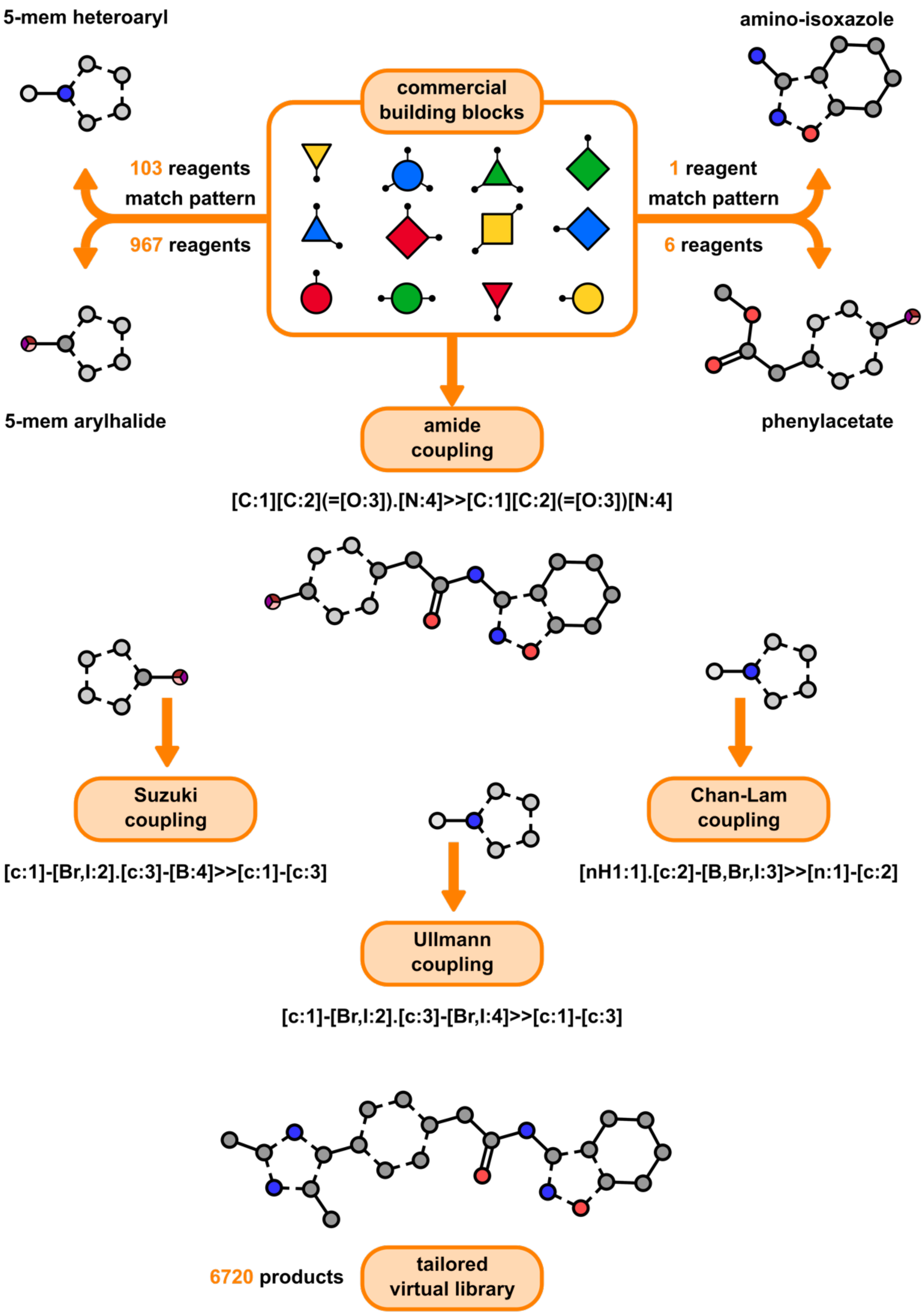

Supplementary Figure S3. Workflow for the generation of the tailored virtual libraries. Chemical pattern matching identified commercially available building blocks that were suitable for Suzuki, Ullmann, Chan-Lam and amide couplings. These building blocks were further pruned to yield topologies (Bemis-Murcko scaffolds) identical to compound **17**. Using SMIRKS patterns, the building blocks were combinatorially coupled to form a tailored virtual library that can be docked to the OGG1 active site.

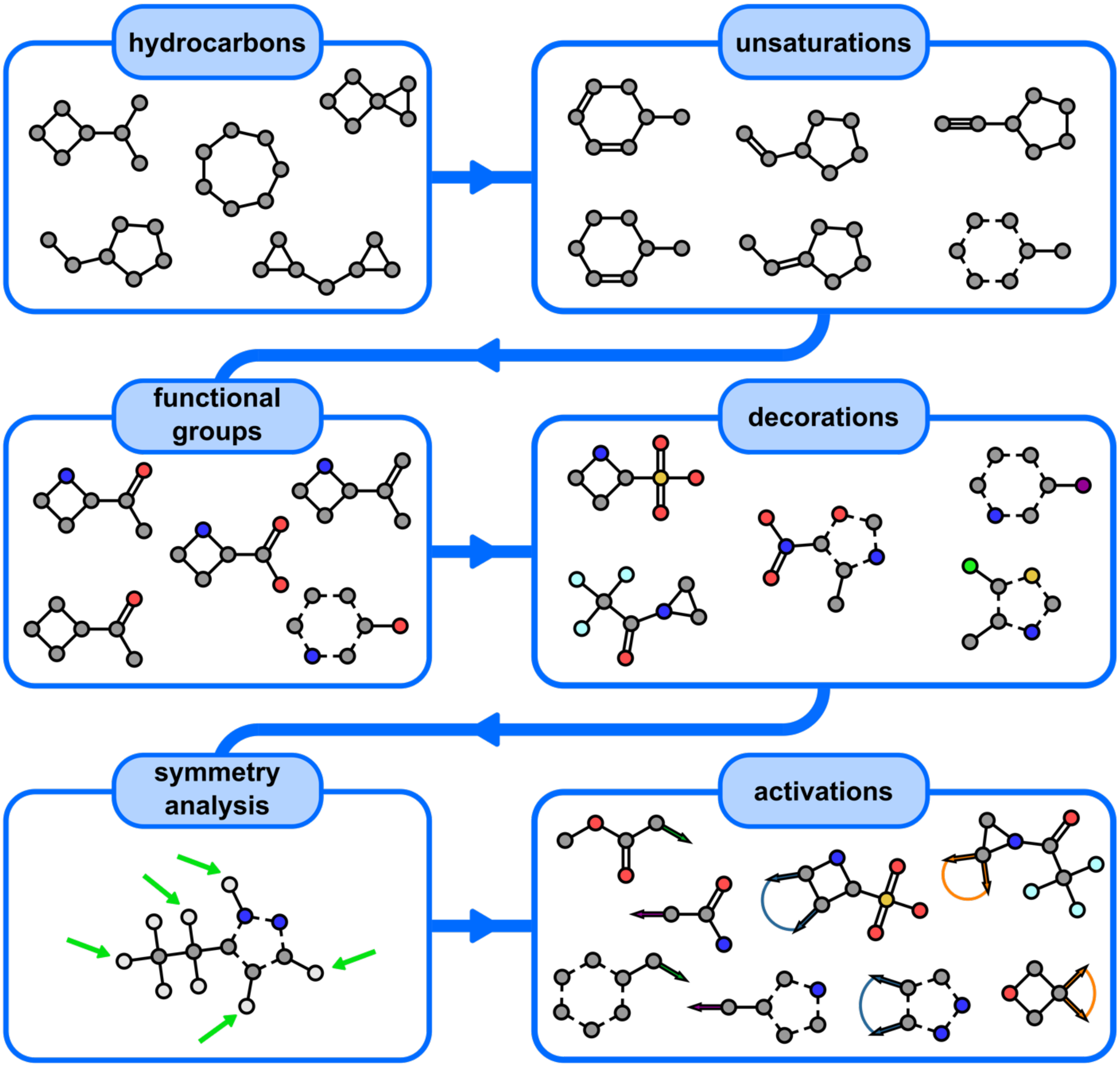

Supplementary Figure S4. Workflow for generation of the substituent space. *Hydrocarbons:* Planar graphs are first generated, followed by conversion into saturated hydrocarbons and embedding into three-dimensional conformations. Hydrocarbons with strained conformations are systematically filtered. *Unsaturations:* The available hydrogens adjacent to each carbon-carbon-bond in the saturated hydrocarbon are analyzed, which determines whether a double or triple bond can be introduced. Multiple unsaturations are introduced via a combinatorial enumeration of viable bonds. *Functional groups:* The available hydrogens on each carbon in a molecule are analyzed, which determines whether those carbons can be mutated into nitrogen or oxygen atoms. Multiple heteroatoms are introduced in a (unsaturated) hydrocarbon via combinatorial enumeration. *Decorations:* Functionalized molecules are further decorated by chemically transforming existing functional groups in a combinatorial manner. *Symmetry analysis:* Hydrogen atoms are analyzed in terms of molecular symmetry to accelerate assignment of activation tags (green vectors) and avoid generation of duplicates. Single, double, and triple couplings require one, two, and three hydrogens, respectively. Spiro-cyclizations require two hydrogens on a single atom, and ring fusions require two atoms with each at least one hydrogen atom. *Activations*: substituents with activation tags are stored and subsequently used for superstructure generation.

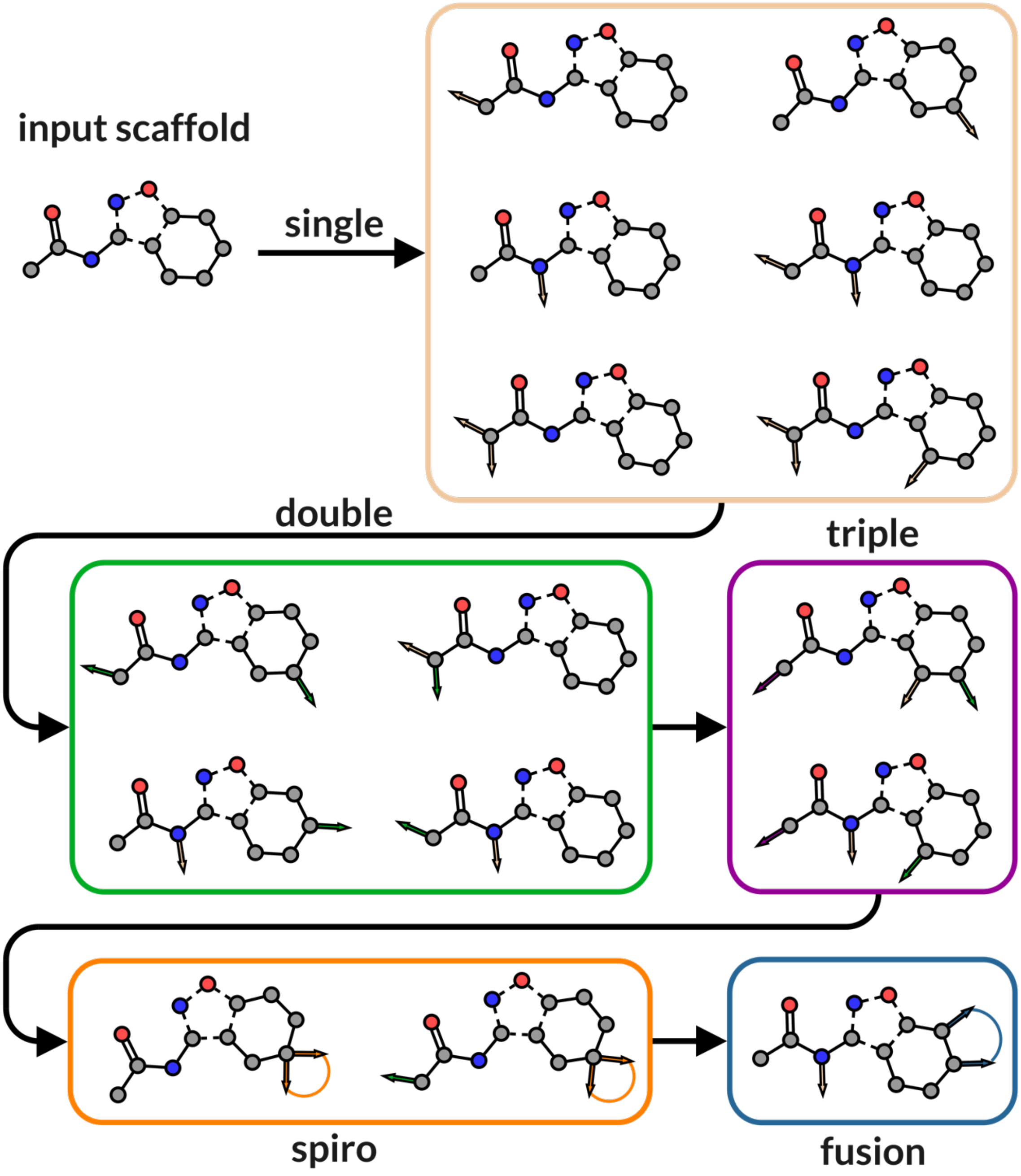

Supplementary Figure S5. Example of scaffold activation. All arrangements to activate an input scaffold are found by iteratively searching for suitable growing vectors. First, all possible growing vectors that correspond to the introduction of a single sigma-bonded substituent (sand-colored vector) are retrieved. Combinatorial enumeration identifies all arrangements with multiple vectors of the same activation type. Each resulting activated compound, including the input scaffold, are subjected to the same vector search algorithm for introduction of double-bonded substituents (green-colored vectors). This results in the formation of mixed type activated compounds, which are iteratively given as input for the remaining activation type search algorithms (purple vectors for triple-bonded substituents, orange vectors for spiro-cyclized substituents, and blue vectors for ring-fused substituents). Note that a single scaffold heavy atom can bear multiple growing vectors of the same type, *e.g.*, introduction of a single sigma-bonded substituent. Activated molecules inside boxes are random samples and hence do not represent the entire activated scaffold space.

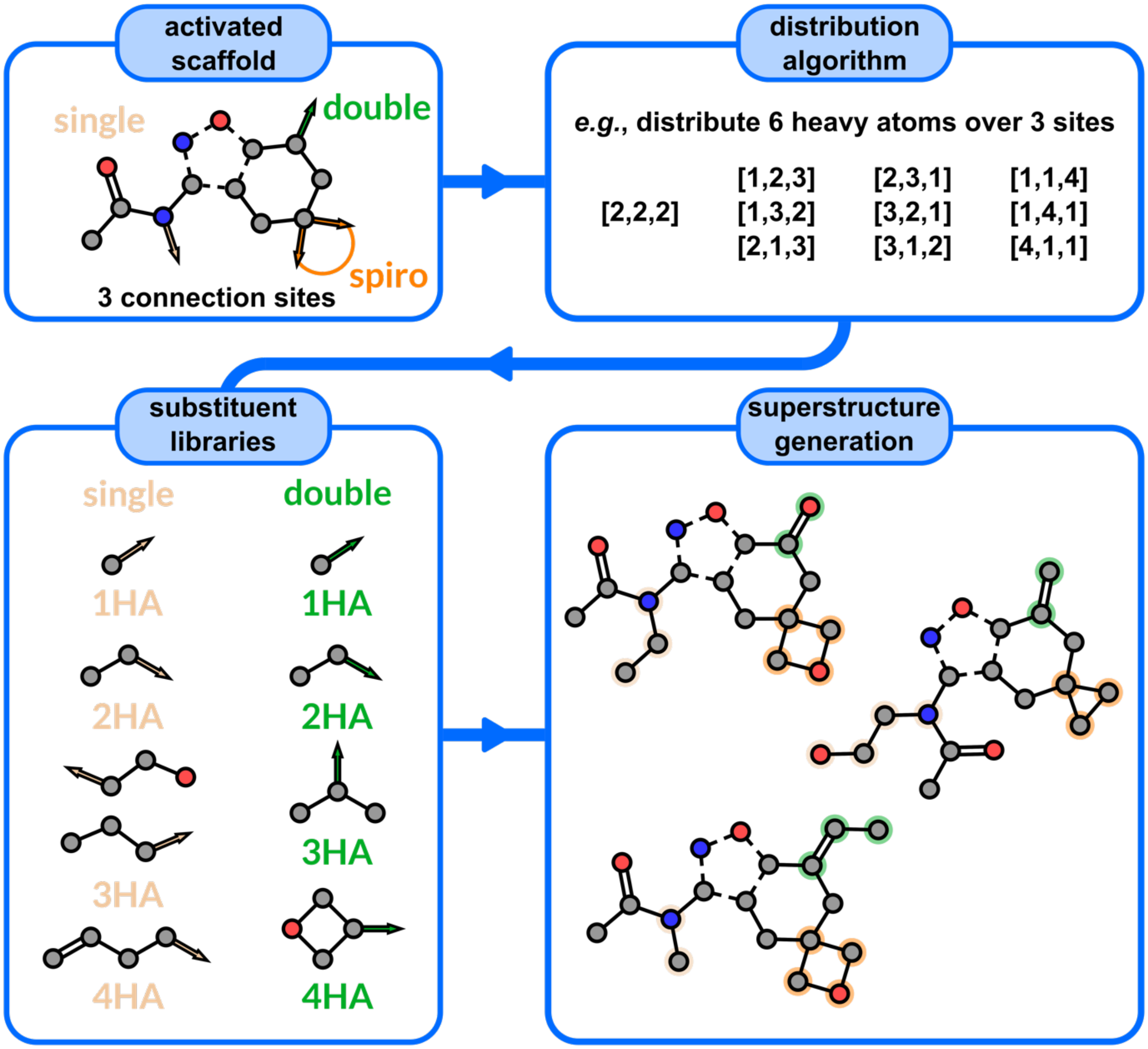

Supplementary Figure S6. Example of superstructure generation. The input scaffold has three connection sites, onto which six additional heavy atoms (HA) are to be distributed. An atom distribution algorithm determines all possible arrangements to place six heavy atoms over three connection sites, after which suitable substituent libraries are retrieved for coupling. Substituents are introduced onto the activated scaffold, and valid superstructures are generated.

Supplementary Methods

Synthesis Procedures

Compounds in Supplementary Table 1-3 were synthesized by Enamine, and the purity of these were at least 90% based on LC/MS data. Compounds **8**-**23** were synthesized in-house according to the following procedure. All reagents were purchased from Fluorochem, Sigma-Aldrich, Enamine and Chemtronica. DCM, methanol, DMF, and acetonitrile (99.9%) were purchased from VWR International AB, whereas THF was purchased from Sigma-Aldrich. Reagents and solvents were used as such without further purification. All reactions involving air or moisture-sensitive reagents or intermediates were performed under a nitrogen atmosphere. Mainly LC-MS was used for monitoring reactions using an Agilent 1100 series HPLC having a C18 Atlantis T3 column (3.0 × 50 mm, 5 μm). Acetonitrile–water (flow rate 0.75 mL/min over 6 min) was used as mobile phase and a Waters micromass Z_Q (model code: MM1) mass spectrometer with electrospray ionization mode was used for detection of molecular ions. TLC (Merck, silica gel 60 F_{254} plates) was sometimes used for monitoring reactions, particularly in purification of compounds. Visualization of the developed TLC was done using UV light (254 nm) and staining with ninhydrin stain or anisaldehyde stain. After workup, organic phases were dried over Na_2SO_4/$MgSO_4$ and filtered before being concentrated under reduced pressure. ^{1}H NMR spectra for the synthesized compounds were recorded at 298 K on an Agilent Technologies 400 MR spectrometer at 400 MHz or 100 MHz, or on Bruker Avance Neo spectrometers at 500/600 MHz or 125/150 MHz. Chemical shifts are reported in parts per million (ppm, δ) referenced to the residual ^{1}H resonance of the solvent ($(CD_3)_2CO$, δ 2.05; $CDCl_3$, δ 7.26; CD_3OD δ 3.31; DMSO-*d*$_6$ δ 2.50). Splitting patterns are designated as follows: s (singlet), d (doublet), t (triplet), m (multiplet) and br (broad). Coupling constants (J) are listed in hertz (Hz). Preparative reversed-phase HPLC was performed on a Kromasil C8 column (250 × 21.2 mm, 5 μm) on a Gilson HPLC equipped with Gilson 322 pump, UV/Visible-156 detector and 202 collector using acetonitrile-water gradients as eluents with a flow rate of 15 mL/min and detection at 210 or 254 nm. All tested compounds were purified by HPLC. ^{1}H NMR spectroscopy (500/600 MHz) and LCMS were used to determine the purity of the tested compounds, which all had a purity $\geq$90%. For the most potent compounds that were extensively characterized in biological assays (**17** and **23**), purity was higher (95%), which was confirmed in-house by LC/MS and NMR spectroscopy.

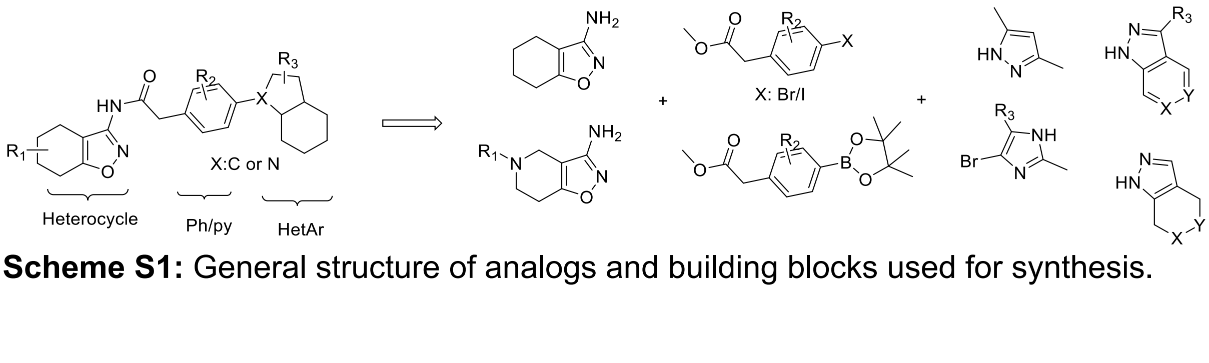

Scheme S1: General structure of analogs and building blocks used for synthesis.

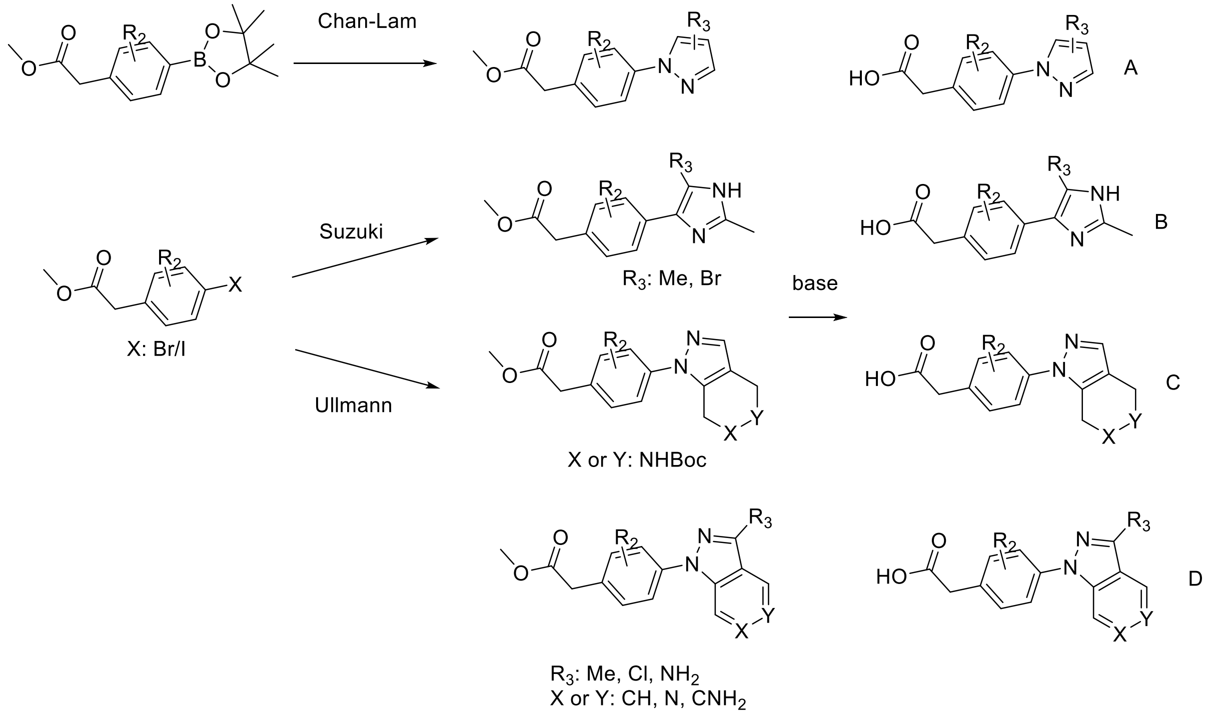

Scheme S2. General strategies for preparation of benzylic acid intermediates.

General procedure for Chan-Lam coupling, synthesis of pyrazole benzylic acid intermediates A

The mixture of substituted 4-Bpin phenyl acetic acid methyl ester (0.1-0.2 mmol), pyrazole derivative (1 equiv), $Cu(OAc)_2.H_2O$ (20% mol), $B(OH)_3$ (2 equiv), 4Å MS (40 mg) in CH_3CN (1-2 mL) was heated at reflux for overnight. After cooling down, the mixture was diluted with ethyl acetate and filtered on celite, concentrated and purified by silica gel column chromatography eluting with hexane:ethyl acetate. The ester product was dissolved in DMSO (1.5 mL). 1 N NaOH was added (4-10 equiv) and the mixture was stirred at rt for 15 min. LCMS confirmed full ester hydrolysis. The mixture was acidified with TFA, filtered and purified by C18 hplc using gradient of 20-80% ACN in H_2O over 30 min (flowrate 15ml/min) to afford the desired product. The product was confirmed by LCMS and used as such for the next step.

The following intermediates were prepared:

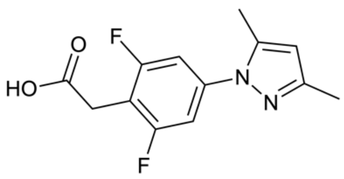

8i

mixture 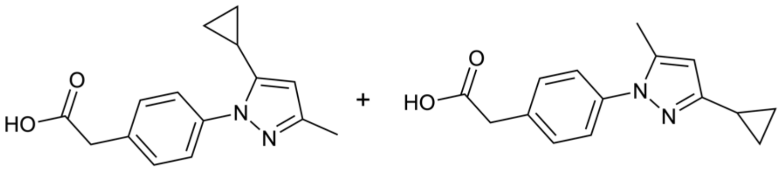

9i

Compound **9i**
^{1}H NMR (600 MHz, CD_3OD) δ 7.53 – 7.36 (m, 4H), 5.91 (s, 1H, iso1), 5.86 (s, 1H, iso2), 3.70 (s, 2H), 2.25 (s, 3H, iso1), 2.22 (s, 3H, iso2), 1.93 – 1.72 (m, 1H), 0.99 – 0.90 (m, 2H), 0.75 – 0.69 (m, 2H).
LCMS (ESI+): calculated for $C_{15}H_{16}N_2O_2$ $(M+H)^+$: 257.1; found 256.9.

General procedure for Suzuki coupling followed by ester hydrolysis, synthesis of imidazole benzylic acid intermediates B
The mixture of substituted 4-Bpin phenyl acetic acid methyl ester (0.2 mmol), $Pd(PPh_3)_4$ (10% mol), Na_2CO_3 (3 equiv), dioxane:H_2O (4:1, 2.5 mL) was degassed and heated at 120 ^{o}C under argon atmosphere for 20 min under MWI. After cooling down, the mixture was diluted with ethyl acetate and filtered on celite. The mixture was washed with brine and concentrated to give a crude which was used as such for the next step or purified by silica gel column chromatography. The ester product was dissolved in DMSO (1.5 mL). 1 N NaOH was added (4-10 equiv) and the mixture was stirred at rt for 15 min. LCMS confirmed full ester hydrolysis. The mixture was acidified with TFA, filtered and purified by C18 hplc using gradient of 5-50% ACN in H_2O over 30 min (flowrate 15ml/min) to afford the desired product. The product was confirmed by LCMS and used as such for the next step.

The following intermediates were prepared:

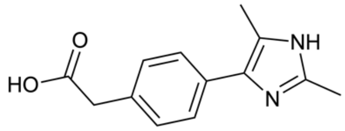

17i

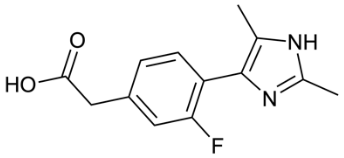

18i

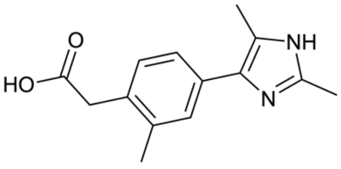

19i

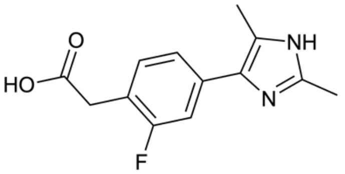

20i

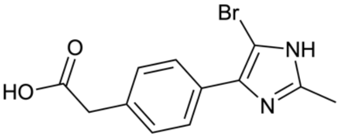

21i

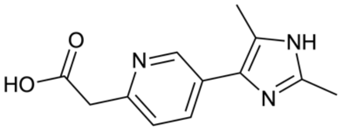

22i

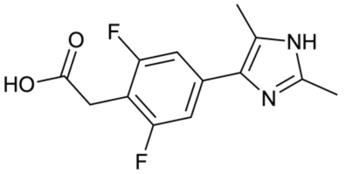

23i

Compound **17i** 2-(4-(2,5-dimethyl-1H-imidazol-4-yl)phenyl)acetic acid

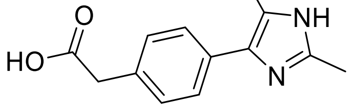

^{1}H NMR (400 MHz, CD_3OD) δ 7.47 – 7.38 (m, 4H), 3.57 (s, 2H), 2.52 (s, 3H), 2.37 (s, 3H).
LCMS (ESI+): calculated for $C_{13}H_{15}N_2O_2$ $(M+H)^+$: 231.1; found 231.2.

Compound **19i** 2-(4-(2,5-dimethyl-1H-imidazol-4-yl)-2-methylphenyl)acetic acid

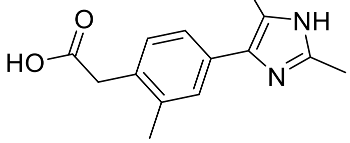

^{1}H NMR (500 MHz, CD_3OD) δ 7.19 – 7.08 (m, 3H), 3.47 (s, 2H), 2.39 (s, 3H), 2.23 (s, 6H).
LCMS (ESI+): calculated for $C_{14}H_{17}N_2O_2$ $(M+H)^+$: 245.1; found 245.2.

General procedure for Ullmann coupling, synthesis of pyrazole and indazole benzylic acid intermediates C and D

The mixture of substituted 4-iodo (or bromo) phenyl acetic acid methyl ester (1-2 equiv), pyrazole or indazole derivative (0.1 mmol), CuI (20% mol), DMEDA (1 equiv), Cs_2CO_3 (2-3 equiv) in DMF (≥ 0.5 M) was degassed and heated at 120 °C under argon atmosphere for overnight. The ester group was hydrolyzed during the reaction. After cooling down and filtered, the mixture was acidified with TFA, filtered and purified by C18 hplc using gradient of 20-100% ACN in H_2O over 30 min (flowrate 15ml/min) to afford the desired product. The product was confirmed by LCMS and used as such for the next step.

The following intermediates were prepared:

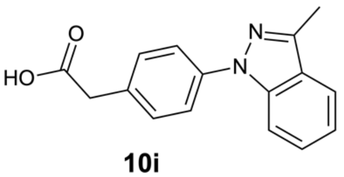

10i

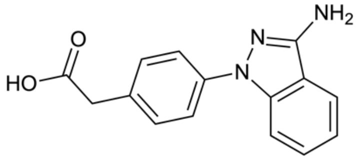

11i

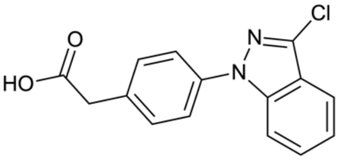

12i

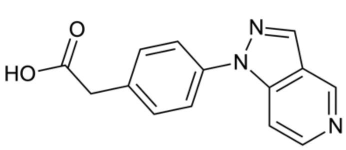

13i

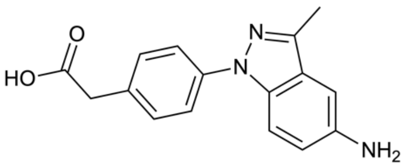

14i

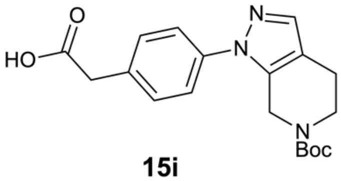

15i

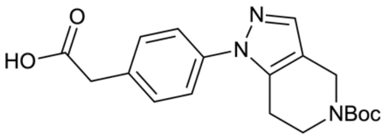

16i

Compound **10i** 2-(4-(3-methyl-1H-indazol-1-yl)phenyl)acetic acid

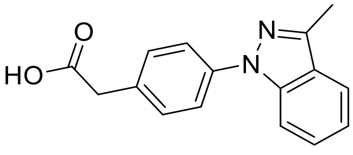

^{1}H NMR (500 MHz, CD_3OD) δ 7.78 (dt, *J* = 8.1, 1.1 Hz, 1H), 7.70 (dd, *J* = 8.6, 1.0 Hz, 1H), 7.67 – 7.61 (m, 2H), 7.51 – 7.42 (m, 3H), 7.26 – 7.19 (m, 1H), 3.70 (s, 2H), 2.62 (s, 3H).
LCMS (ESI+): calculated for $C_{16}H_{15}N_2O_2$ $(M+H)^+$: 267.1; found 267.2.

Compound **16i** 2-(4-(5-(tert-butoxycarbonyl)-4,5,6,7-tetrahydro-1H-pyrazolo[4,3-c]pyridin-1-yl)phenyl)acetic acid

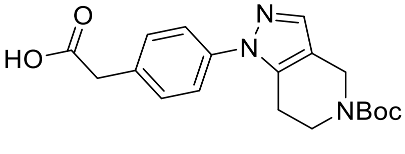

^{1}H NMR NMR (400 MHz, CD_3OD) δ 7.99 (s, 1H), 7.63 (d, *J* = 8.2 Hz, 2H), 7.39 (d, *J* = 8.2 Hz, 2H), 4.55 (s, 2H), 3.75 (t, *J* = 5.9 Hz, 2H), 3.64 (s, 2H), 2.80 (t, *J* = 5.9 Hz, 2H), 1.49 (s, 9H).
LCMS (ESI+): calculated for $C_{19}H_{24}N_3O_4$ $(M+H)^+$: 358.2; found 358.2.

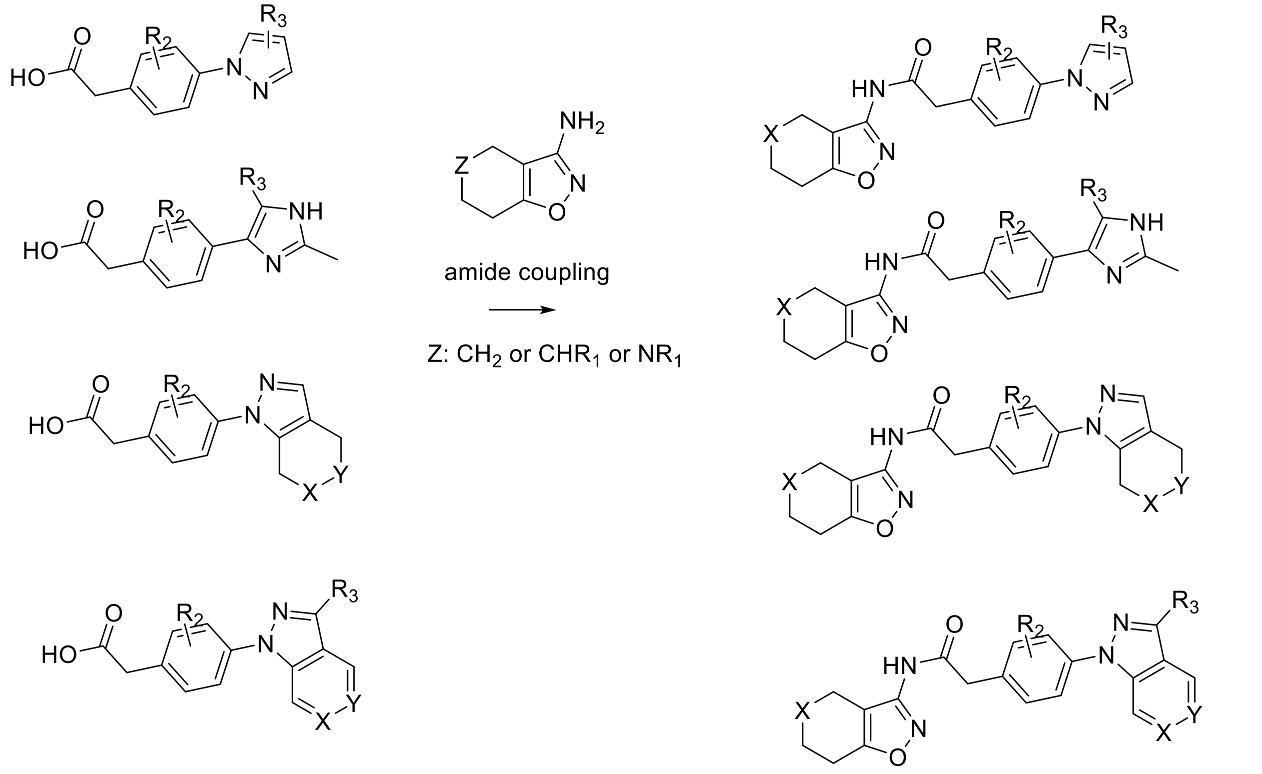

General procedure for amide coupling, synthesis of compounds 8-23
The mixture of acid (1 equiv), amine (1 equiv), DMTMM 4-(4,6-Dimethoxy-1,3,5-triazin-2-yl)-4-methylmorpholin-4-ium chloride (2 equiv), DIEA (3 equiv) in DMF was stirred at rt for overnight. Then the mixture was acidified with TFA, filtered and purified by C18 hplc using gradient 5-50% of ACN in H_2O (H_2O + 0.1% TFA) over 30 min (flowrate 15ml/min) to afford desired product.

Compound **21** 2-(4-(5-bromo-2-methyl-1H-imidazol-4-yl)phenyl)-N-(4,5,6,7-tetrahydrobenzo[d]isoxazol-3-yl)acetamide
Purity NMR + LCMS 95%+

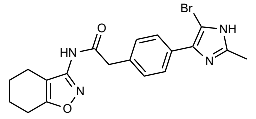

^{1}H NMR (600 MHz, CD_3OD) δ 7.70 (d, *J* = 7.9 Hz, 2H), 7.50 (d, *J* = 7.9 Hz, 2H), 3.81 (s, 2H), 2.65 (td, *J* = 6.4, 1.4 Hz, 2H), 2.58 (d, *J* = 1.1 Hz, 3H), 2.39 (t, *J* = 6.2 Hz, 2H), 1.90 – 1.83 (m, 2H), 1.77 – 1.70 (m, 2H).
^{13}C NMR (150 MHz, CD_3OD) δ 171.7, 170.7, 157.6, 147.1, 137.7, 131.1, 130.8, 128.1, 127.1, 109.4, 103.5, 43.2, 23.6, 23.4, 23.1, 21.3, 12.2.

LCMS (ESI+): calculated for $C_{19}H_{20}BrN_4O_2$ $(M+H)^+$: 415.1; found 415.0.

Compound **18** 2-(4-(2,5-dimethyl-1H-imidazol-4-yl)-3-fluorophenyl)-N-(4,5,6,7-tetrahydrobenzo[d]isoxazol-3-yl)acetamide
Purity NMR + LCMS 95%+

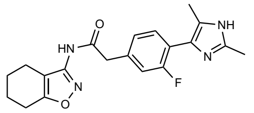

^{1}H NMR (600 MHz, CD_3OD) δ 7.49 (t, *J* = 7.7 Hz, 1H), 7.34 (d, *J* = 9.5 Hz, 2H), 3.85 (s, 2H), 2.66 (dt, *J* = 7.7, 3.8 Hz, 2H), 2.64 (d, *J* = 1.2 Hz, 3H), 2.43 – 2.38 (m, 2H), 2.34 (s, 3H), 1.88 (p, *J* = 6.1 Hz, 2H), 1.79 – 1.72 (m, 2H).
^{13}C NMR (150 MHz, CD_3OD) δ 171.0, 170.7, 161.6, 160.0, 157.6, 144.9, 141.0, 141.0, 131.7, 127.9, 127.2, 123.6, 118.5, 118.3, 115.0, 109.3, 42.8, 23.6, 23.4, 23.1, 21.3, 11.3, 9.8.
LCMS (ESI+): calculated for $C_{20}H_{22}FN_4O_2$ $(M+H)^+$: 369.2; found 369.0.

Compound **22** 2-(5-(2,5-dimethyl-1H-imidazol-4-yl)pyridin-2-yl)-N-(4,5,6,7-tetrahydrobenzo[d]isoxazol-3-yl)acetamide
Purity NMR + LCMS 90%

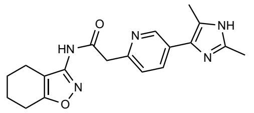

^{1}H NMR (600 MHz, CD_3OD) δ 8.71 (s, 1H), 7.99 (dt, *J* = 8.1, 1.8 Hz, 1H), 7.62 (d, *J* = 8.1 Hz, 1H), 2.69 – 2.62 (m, 5H), 2.46 (s, 3H), 2.44 – 2.40 (m, 2H), 1.88 (p, *J* = 5.8 Hz, 2H), 1.75 (ddt, *J* = 9.0, 6.2, 3.5 Hz, 2H).
LCMS (ESI+): calculated for $C_{19}H_{22}N_5O_2$ $(M+H)^+$: 352.2; found 352.0.

Compound **11** 2-(4-(3-amino-1H-indazol-1-yl)phenyl)-N-(4,5,6,7-tetrahydrobenzo[d]isoxazol-3-yl)acetamide
Purity NMR + LCMS 90%

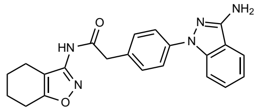

^{1}H NMR (600 MHz, CD_3OD) δ 7.81 (d, *J* = 8.1 Hz, 1H), 7.70 – 7.60 (m, 3H), 7.53 – 7.45 (m, 3H), 7.17 (s, 1H), 3.80 (s, 2H), 2.66 (td, *J* = 6.2, 1.5 Hz, 2H), 2.39 (t, *J* = 6.2 Hz, 2H), 1.90 – 1.84 (m, 2H), 1.77 – 1.71 (m, 2H).
LCMS (ESI+): calculated for $C_{22}H_{22}N_5O_2$ $(M+H)^+$: 388.2; found 388.0.

Compound **12** 2-(4-(3-chloro-1H-indazol-1-yl)phenyl)-N-(4,5,6,7-tetrahydrobenzo[d]isoxazol-3-yl)acetamide
Purity NMR + LCMS 95%+

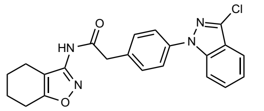

^{1}H NMR (600 MHz, DMSO-d6) δ 10.71 (s, 1H), 7.88 (d, *J* = 8.6 Hz, 1H), 7.81 (dd, *J* = 8.1, 1.2 Hz, 1H), 7.75 – 7.71 (m, 2H), 7.61 (ddt, *J* = 8.3, 6.9, 1.2 Hz, 1H), 7.53 (d, *J* = 8.1 Hz, 2H), 7.39 (t, *J* = 7.5 Hz, 1H), 3.78 (s, 2H), 2.63 (dt, *J* = 6.3, 3.2 Hz, 2H), 2.33 – 2.28 (m, 2H), 1.77 (p, *J* = 5.7 Hz, 2H), 1.62 (dp, *J* = 8.3, 3.2 Hz, 2H).
^{13}C NMR (150 MHz, DMSO-d6) δ 169.2, 168.8, 156.7, 139.4, 137.5, 134.6, 134.5, 130.5, 129.1, 122.9, 122.3, 121.5, 119.5, 111.3, 108.4, 41.5, 22.3, 21.9, 21.6, 20.5.

LCMS (ESI+): calculated for $C_{22}H_{20}ClN_4O_2$ $(M+H)^+$: 407.1; found 407.0.

Compound **13** 2-(4-(1H-pyrazolo[3,4-c]pyridin-1-yl)phenyl)-N-(4,5,6,7-tetrahydrobenzo[d]isoxazol-3-yl)acetamide
Purity NMR + LCMS 95%

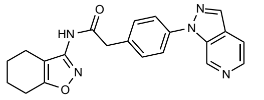

^{1}H NMR (600 MHz, CD_3OD) δ 9.51 (s, 1H), 8.67 (s, 1H), 8.45 (d, *J* = 6.1 Hz, 1H), 8.29 (d, *J* = 6.0 Hz, 1H), 7.83 (d, *J* = 8.0 Hz, 2H), 7.65 (d, *J* = 8.0 Hz, 2H), 3.89 (s, 2H), 2.66 (dt, *J* = 7.7, 3.9 Hz, 2H), 2.42 (t, *J* = 6.3 Hz, 2H), 1.88 (p, *J* = 6.1 Hz, 2H), 1.75 (qd, *J* = 6.0, 2.5 Hz, 2H).
^{13}C NMR (150 MHz, CD_3OD) δ 171.7, 170.7, 157.7, 138.9, 137.1, 136.9, 136.1, 134.8, 133.3, 132.9, 132.0, 124.3, 119.3, 109.4, 42.9, 23.6, 23.5, 23.2, 21.3.
LCMS (ESI+): calculated for $C_{21}H_{20}N_5O_2$ $(M+H)^+$: 374.2; found 374.0.

Compound **15** 2-(4-(4,5,6,7-tetrahydro-1H-pyrazolo[3,4-c]pyridin-1-yl)phenyl)-N-(4,5,6,7-tetrahydrobenzo[d]isoxazol-3-yl)acetamide
Purity NMR + LCMS 90%+

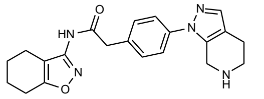

^{1}H NMR (600 MHz, CD_3OD) δ 7.62 (s, 1H), 7.53 (d, *J* = 8.1 Hz, 2H), 7.46 (d, *J* = 8.1 Hz, 2H), 4.57 (s, 2H), 4.38 (s, 3H), 3.83 (s, 2H), 2.93 (s, 2H), 2.66 (t, *J* = 6.4 Hz, 2H), 2.40 (s, 2H), 1.87 (d, *J* = 5.8 Hz, 2H), 1.74 (d, *J* = 6.8 Hz, 2H).
LCMS (ESI+): calculated for $C_{21}H_{24}N_5O_2$ $(M+H)^+$: 378.2; found 378.0.

Compound **16** 2-(4-(4,5,6,7-tetrahydro-1H-pyrazolo[4,3-c]pyridin-1-yl)phenyl)-N-(4,5,6,7-tetrahydrobenzo[d]isoxazol-3-yl)acetamide
Purity NMR + LCMS 90%+

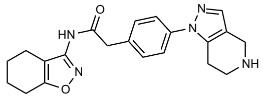

^{1}H NMR (600 MHz, CD_3OD) δ 8.17 (s, 1H), 7.69 (d, *J* = 8.2 Hz, 2H), 7.47 (d, *J* = 8.1 Hz, 2H), 4.35 (s, 2H), 3.78 (s, 2H), 3.57 (t, *J* = 6.3 Hz, 2H), 3.11 (t, *J* = 6.3 Hz, 2H), 2.67 – 2.64 (m, 2H), 2.41 – 2.36 (m, 2H), 1.87 (td, *J* = 7.6, 4.1 Hz, 2H), 1.76 – 1.71 (m, 2H).
LCMS (ESI+): calculated for $C_{21}H_{24}N_5O_2$ $(M+H)^+$: 378.2; found 378.0.

Compound **14** 2-(4-(5-amino-3-methyl-1H-indazol-1-yl)phenyl)-N-(4,5,6,7-tetrahydrobenzo[d]isoxazol-3-yl)acetamide
Purity NMR + LCMS 95%+

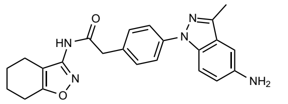

^{1}H NMR (600 MHz, CD_3OD) δ 7.87 (d, *J* = 8.9 Hz, 1H), 7.78 (d, *J* = 2.1 Hz, 1H), 7.68 (d, *J* = 8.1 Hz, 2H), 7.57 (d, *J* = 8.0 Hz, 2H), 7.44 (dt, *J* = 8.9, 1.5 Hz, 1H), 3.85 (s, 2H), 2.69 – 2.62 (m, 5H), 2.41 (t, *J* = 6.2 Hz, 2H), 1.91 – 1.84 (m, 2H), 1.78 – 1.72 (m, 2H).
^{13}C NMR (150 MHz, CD_3OD) δ 172.0, 170.7, 157.7, 145.5, 140.0, 139.8, 135.4, 131.6, 126.3, 126.0, 123.8, 123.3, 115.6, 113.4, 109.4, 43.0, 23.6, 23.4, 23.1, 21.3, 11.6.
LCMS (ESI+): calculated for $C_{23}H_{24}N_5O_2$ $(M+H)^+$: 402.2; found 402.0.

Compound **23** 2-(4-(2,5-dimethyl-1H-imidazol-4-yl)-2,6-difluorophenyl)-N-(4,5,6,7-tetrahydrobenzo[d]isoxazol-3-yl)acetamide
Purity NMR + LCMS 95%+

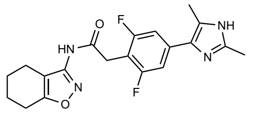

^{1}H NMR (600 MHz, CD_3OD) δ 7.24 (d, *J* = 7.4 Hz, 2H), 3.93 (s, 2H), 2.66 (tt, *J* = 6.3, 1.6 Hz, 2H), 2.64 (s, 3H), 2.46 (s, 3H), 2.41 (tt, *J* = 6.2, 1.6 Hz, 2H), 1.91 – 1.84 (m, 2H), 1.79 – 1.72 (m, 2H).
^{13}C NMR (150 MHz, CD_3OD) δ 170.7, 169.3, 164.2, 164.2, 163.0, 162.7, 162.6, 162.5, 157.5, 145.1, 130.3, 127.4, 127.2, 113.2, 113.1, 111.2, 111.2, 111.1, 111.0, 109.2, 30.0, 23.6, 23.4, 23.1, 21.2, 11.3, 10.2.
LCMS (ESI+): calculated for $C_{20}H_{21}F_2N_4O_2$ $(M+H)^+$: 387.2; found 387.2.

Compound **20** 2-(4-(2,5-dimethyl-1H-imidazol-4-yl)-2-fluorophenyl)-N-(4,5,6,7-tetrahydrobenzo[d]isoxazol-3-yl)acetamide
Purity NMR + LCMS 95%+

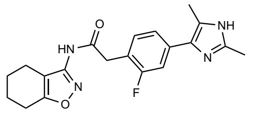

^{1}H NMR (600 MHz, CD_3OD) δ 7.54 (t, *J* = 7.8 Hz, 1H), 7.35 (ddd, *J* = 12.1, 9.1, 1.8 Hz, 2H), 3.89 (s, 2H), 2.69 – 2.65 (m, 2H), 2.64 (s, 3H), 2.45 (s, 3H), 2.41 (t, *J* = 6.2 Hz, 2H), 1.88 (ddt, *J* = 9.9, 6.5, 3.4 Hz, 2H), 1.78 – 1.72 (m, 2H).
^{13}C NMR (150 MHz, CD_3OD) δ 170.7, 163.6, 162.0, 157.6, 144.7, 134.1, 134.1, 129.9, 128.1, 126.6, 124.6, 124.5, 124.2, 115.1, 115.0, 109.3, 36.4, 23.6, 23.4, 23.2, 21.2, 11.2, 10.1.
LCMS (ESI+): calculated for $C_{20}H_{22}FN_4O_2$ $(M+H)^+$: 369.2; found 369.2.

Compound **19** 2-(4-(2,5-dimethyl-1H-imidazol-4-yl)-2-methylphenyl)-N-(4,5,6,7-tetrahydrobenzo[d]isoxazol-3-yl)acetamide
Purity NMR + LCMS 95%+

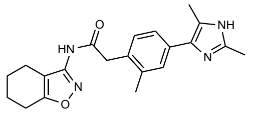

^{1}H NMR (600 MHz, CD_3OD) δ 7.43 – 7.32 (m, 3H), 3.87 (s, 2H), 2.66 (t, *J* = 6.4 Hz, 2H), 2.63 (s, 3H), 2.46 – 2.35 (m, 8H), 1.91 – 1.85 (m, 2H), 1.75 (dp, *J* = 8.9, 2.8 Hz, 2H).
^{13}C NMR (150 MHz, CD_3OD) δ 171.6, 170.7, 162.8, 162.6, 157.7, 144.2, 139.7, 136.2, 132.3, 129.9, 129.3, 127.7, 126.0, 125.6, 109.4, 40.9, 23.6, 23.4, 23.1, 21.3, 19.8, 11.2, 10.1.
LCMS (ESI+): calculated for $C_{21}H_{25}N_4O_2$ $(M+H)^+$: 365.2; found 365.2.

Compound **17** 2-(4-(2,5-dimethyl-1H-imidazol-4-yl)phenyl)-N-(4,5,6,7-tetrahydrobenzo[d]isoxazol-3-yl)acetamide
Purity NMR + LCMS 95%+

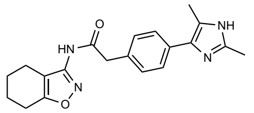

^{1}H NMR (500 MHz, CD_3OD) δ 7.58 – 7.44 (m, 4H), 3.81 (s, 2H), 2.66 (td, *J* = 4.7, 2.3 Hz, 2H), 2.63 (s, 3H), 2.43 (s, 3H), 2.39 (tt, *J* = 6.1, 1.5 Hz, 2H), 1.92 – 1.82 (m, 2H), 1.79 – 1.70 (m, 2H).
^{13}C NMR (125 MHz, CD_3OD) δ 171.7, 170.7, 157.6, 144.3, 137.5, 131.3, 129.1, 128.5, 127.6, 125.8, 109.3, 43.1, 23.6, 23.4, 23.1, 21.3, 11.2, 10.1.

LCMS (ESI+): calculated for $C_{20}H_{23}N_4O_2$ $(M+H)^+$: 351.2; found 351.3.

Compound **9** 2-(4-(5-cyclopropyl-3-methyl-1H-pyrazol-1-yl)phenyl)-N-(4,5,6,7-tetrahydrobenzo[d]isoxazol-3-yl)acetamide
3:2 mixture of isomers
Purity NMR + LCMS 95%+

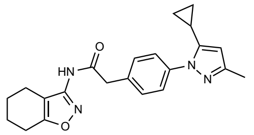

^{1}H NMR (500 MHz, CD_3OD) δ 7.57 – 7.36 (m, 4H), 5.91 (s, 1H), 3.81 (s, 2H), 2.65 (tt, *J* = 6.4, 1.6 Hz, 2H), 2.38 (tt, *J* = 6.1, 1.6 Hz, 2H), 2.24 (s, 3H), 1.94 – 1.69 (m, 5H), 0.99 – 0.88 (m, 2H), 0.72 (ddt, *J* = 9.3, 6.6, 4.3 Hz, 2H).
^{13}C NMR (125 MHz, CD_3OD) δ 170.7, 157.7, 157.3, 150.4, 148.9, 141.7, 139.8, 136.3, 136.1, 131.1, 131.1, 126.5, 126.3, 104.0, 103.6, 43.0, 23.6, 23.4, 23.1, 21.3, 13.2, 12.2, 9.7, 9.2, 8.4, 8.3.
LCMS (ESI+): calculated for $C_{22}H_{25}N_4O_2$ $(M+H)^+$: 377.2; found 377.3.

Compound **8** 2-(4-(3,5-dimethyl-1H-pyrazol-1-yl)-2,6-difluorophenyl)-N-(4,5,6,7-tetrahydrobenzo[d]isoxazol-3-yl)acetamide
Purity NMR + LCMS 95%

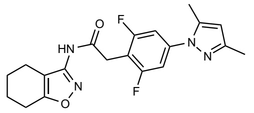

^{1}H NMR (600 MHz, CD_3OD) δ 7.23 – 7.14 (m, 2H), 6.11 (s, 1H), 3.91 (s, 2H), 2.66 (t, *J* = 6.4 Hz, 2H), 2.41 (t, *J* = 6.2 Hz, 2H), 2.36 (s, 3H), 2.25 (s, 3H), 1.92 – 1.84 (m, 2H), 1.79 – 1.72 (m, 2H).
^{13}C NMR (150 MHz, CD_3OD) δ 170.7, 169.5, 163.7, 162.0, 157.6, 151.4, 142.2, 141.5, 109.1, 108.9, 108.7, 49.4, 49.3, 49.1, 49.0, 48.9, 48.7, 48.6, 29.9, 23.6, 23.5, 23.2, 21.2, 13.2, 12.4.
LCMS (ESI+): calculated for $C_{20}H_{21}F_2N_4O_2$ $(M+H)^+$: 387.2; found 387.1.

Compound **10** 2-(4-(3-methyl-1H-indazol-1-yl)phenyl)-N-(4,5,6,7-tetrahydrobenzo[d]isoxazol-3-yl)acetamide
Purity NMR + LCMS 95%+

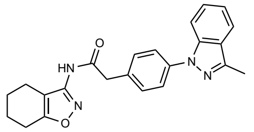

^{1}H NMR (500 MHz, CD_3OD) δ 7.79 (dd, *J* = 8.1, 1.1 Hz, 1H), 7.72 (d, *J* = 8.5 Hz, 1H), 7.70 – 7.66 (m, 2H), 7.55 (d, *J* = 8.1 Hz, 2H), 7.47 (ddd, *J* = 8.3, 6.9, 1.2 Hz, 1H), 7.24 (dd, *J* = 8.0, 7.0 Hz, 1H), 3.83 (s, 2H), 2.69 – 2.64 (m, 2H), 2.63 (s, 3H), 2.40 (t, *J* = 6.0 Hz, 2H), 1.90 – 1.84 (m, 2H), 1.77 – 1.71 (m, 2H).
^{13}C NMR (150 MHz, CD_3OD) δ 172.0, 170.7, 157.7, 145.4, 140.9, 140.3, 134.7, 131.5, 128.9, 125.9, 123.7, 122.3, 121.8, 111.3, 109.5, 43.0, 23.6, 23.5, 23.2, 21.3, 11.7.
LCMS (ESI+): calculated for $C_{23}H_{23}N_4O_2$ $(M+H)^+$: 387.2; found 387.3.

LCMS Spectral Data

LCMS of **Compound 1**

MaxPeak: 100.00%
Ret_Time: 1.356 min

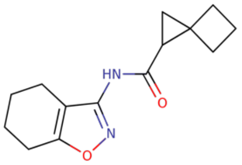

Mol Wt 246.3
Exact Mass 246.16

#	Time	Area%
1	1.356	100.00

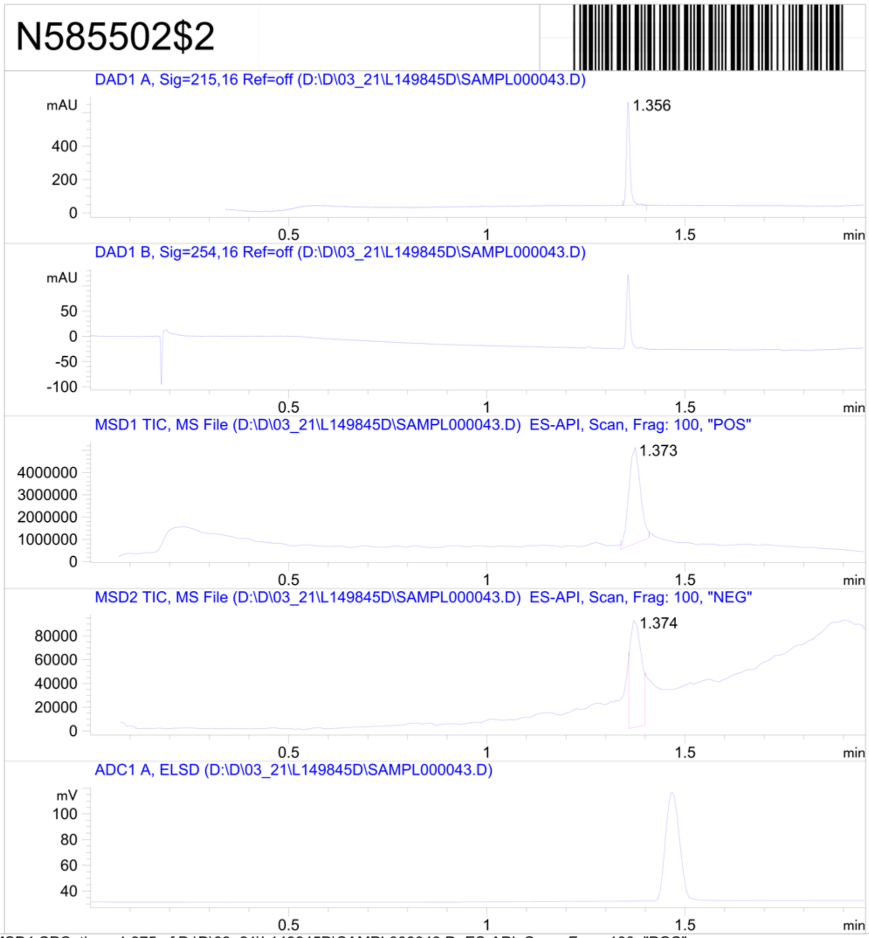

RT 1.373

RT 1.374

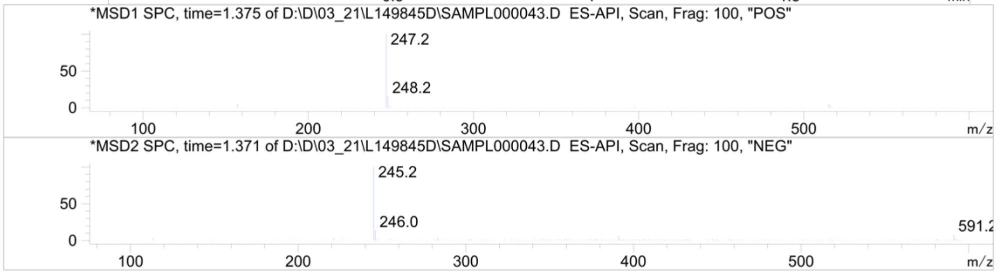

Inj.Date 3/21/2019 N P2-E-06 -3- Acq. Method C:\CHEM32\-> ->

LCMS of **Compound 2**

MaxPeak: 100.00%
Ret_Time: 1.092 min

6503799206

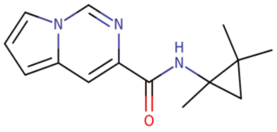

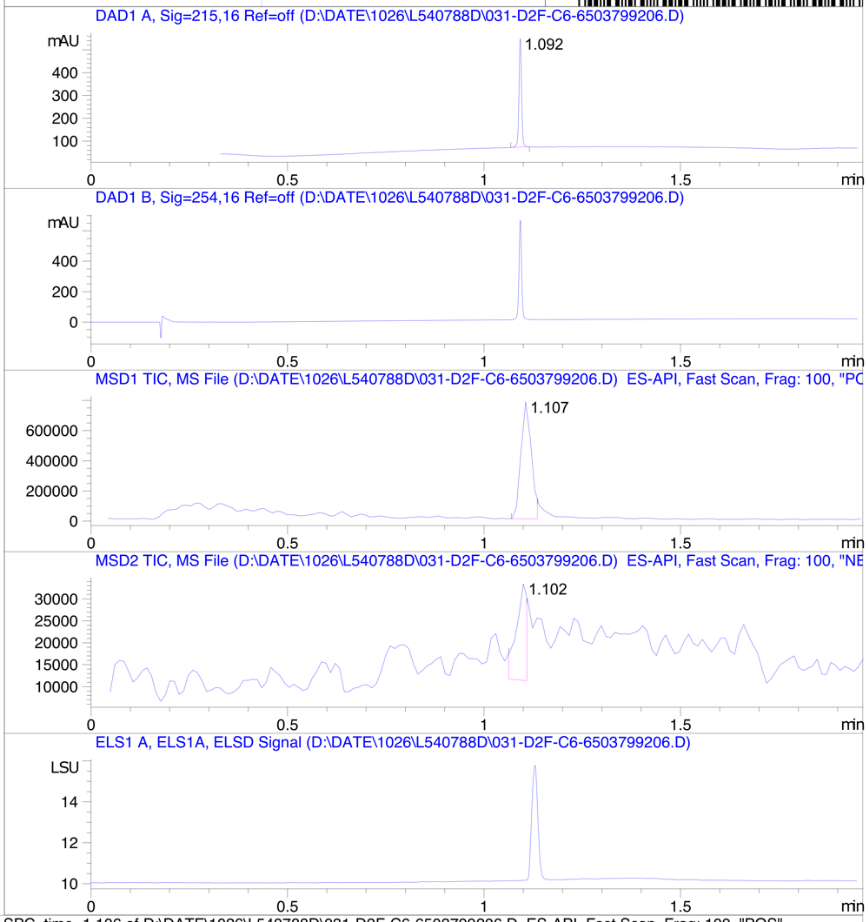

Mol Wt 243.3

Exact Mass 243.16

#	Time	Area%
1	1.092	100.00

RT 1.107

RT 1.102

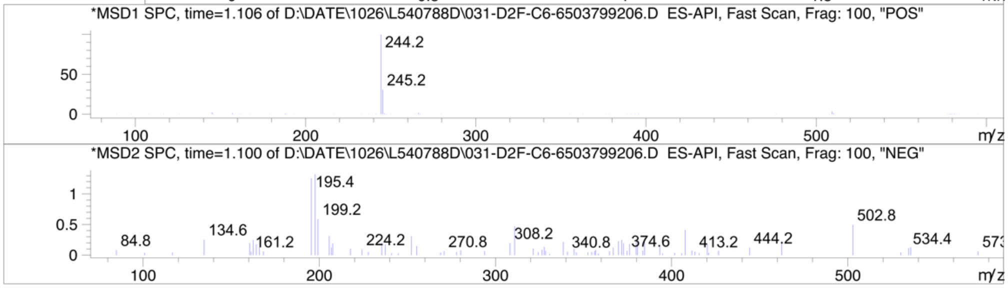

Inj.Date 10/26/2022 E Acq. Method C:\Users\ -> ->

LCMS of **Compound 3**

MaxPeak: 100.00%
Ret_Time: 1.187 min

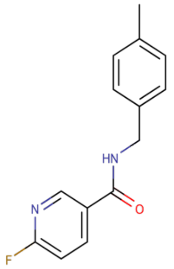

Mol Wt 244.26
Exact Mass 244.12

#	Time	Area%
1	1.187	100.00

RT 1.205

RT 1.206

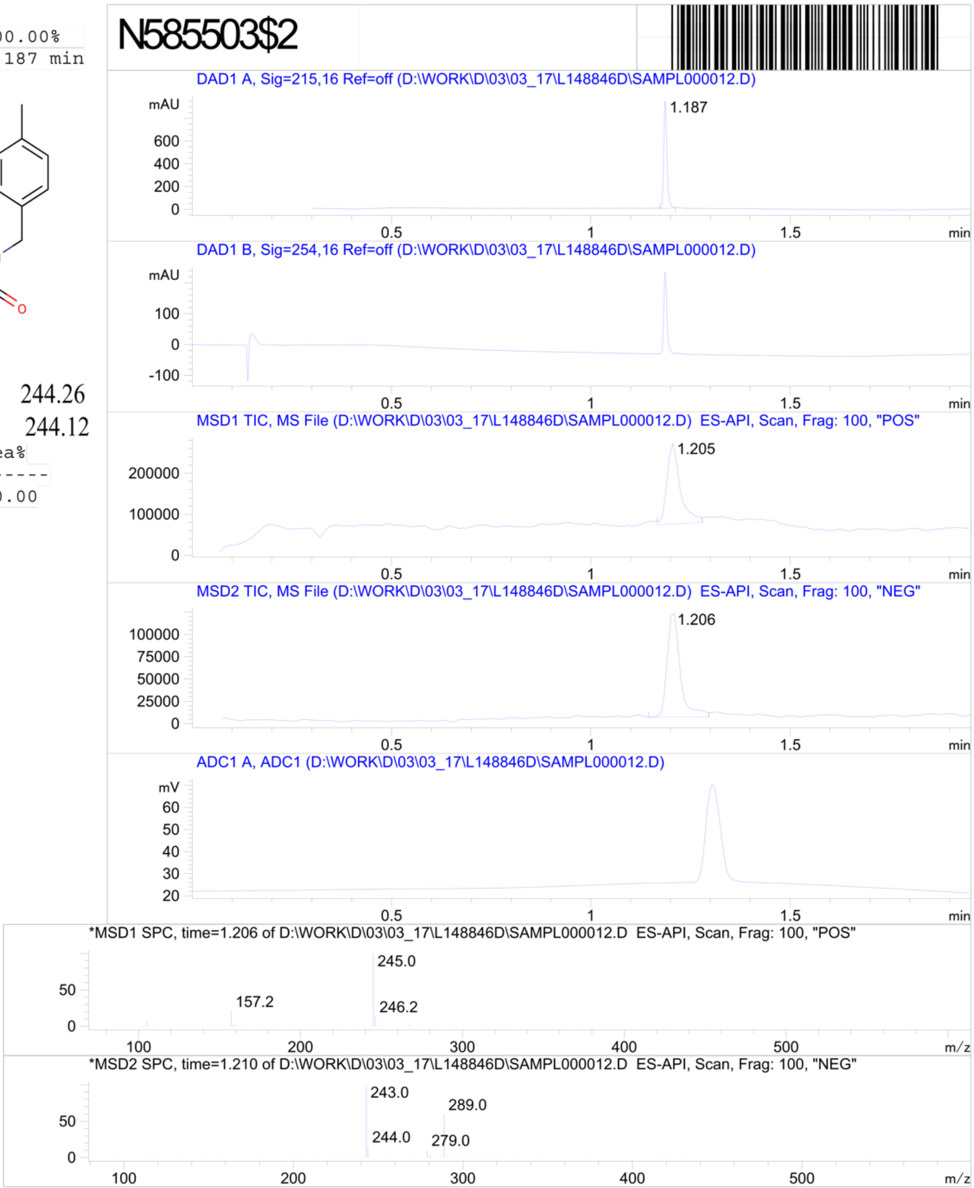

Inj.Date 3/16/2019 K P2-B-04 - 4 - Acq. Method C:\CHEM32\-> ->

LCMS of **Compound 4**

MaxPeak: 100.00%
Ret_Time: 0.755 min

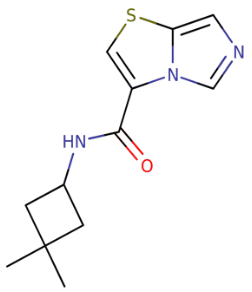

Mol Wt 249.33
Exact Mass 249.11

#	Time	Area%
1	0.755	100.00

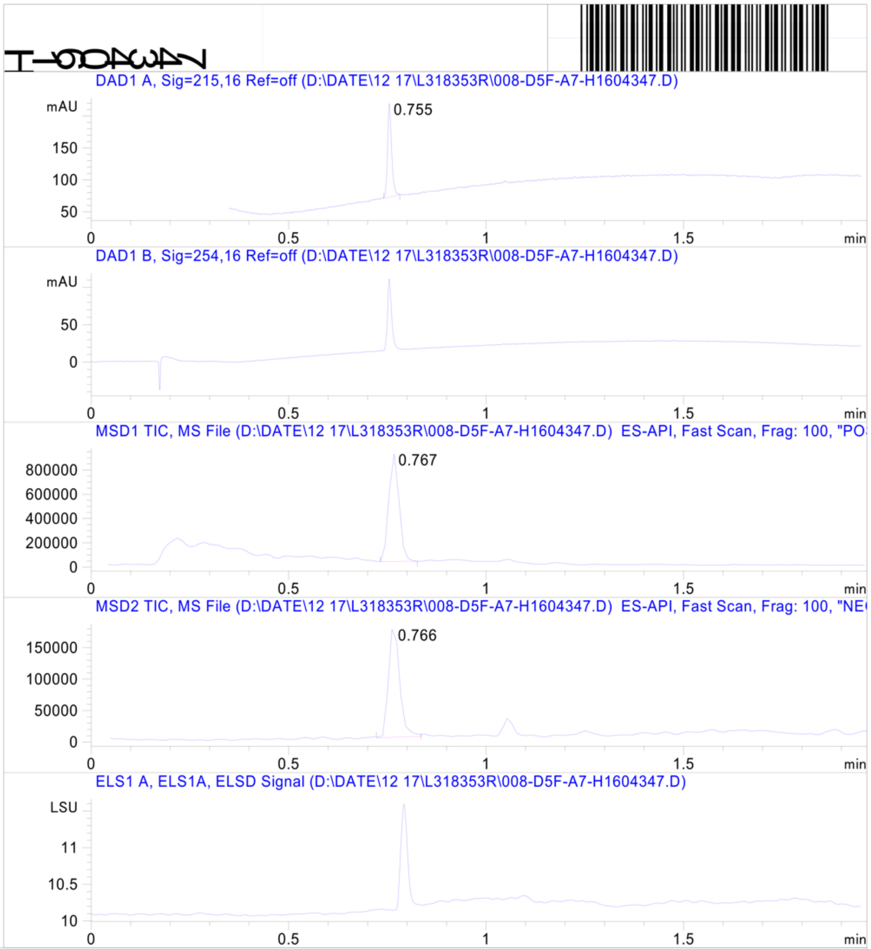

RT 0.767

RT 0.766

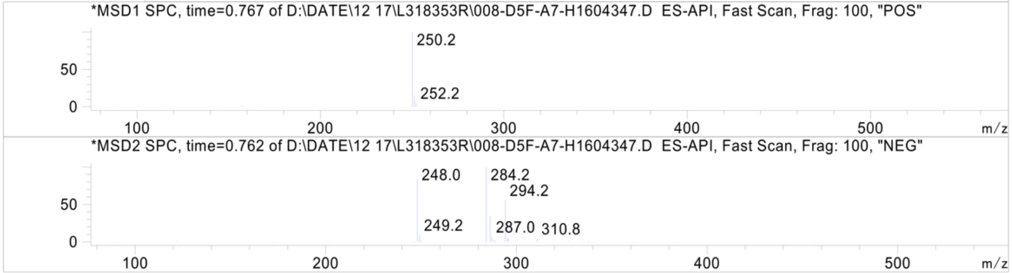

Inj.Date 12/17/2020 E Acq. Method C:\Users\ -> ->

LCMS of **Compound 5**

MaxPeak: 100.00%
Ret_Time: 1.311 min

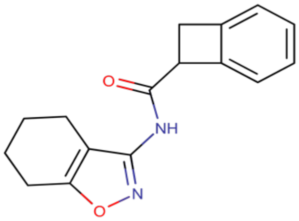

Mol Wt 268.31
Exact Mass 268.14

#	Time	Area%
1	1.311	100.00

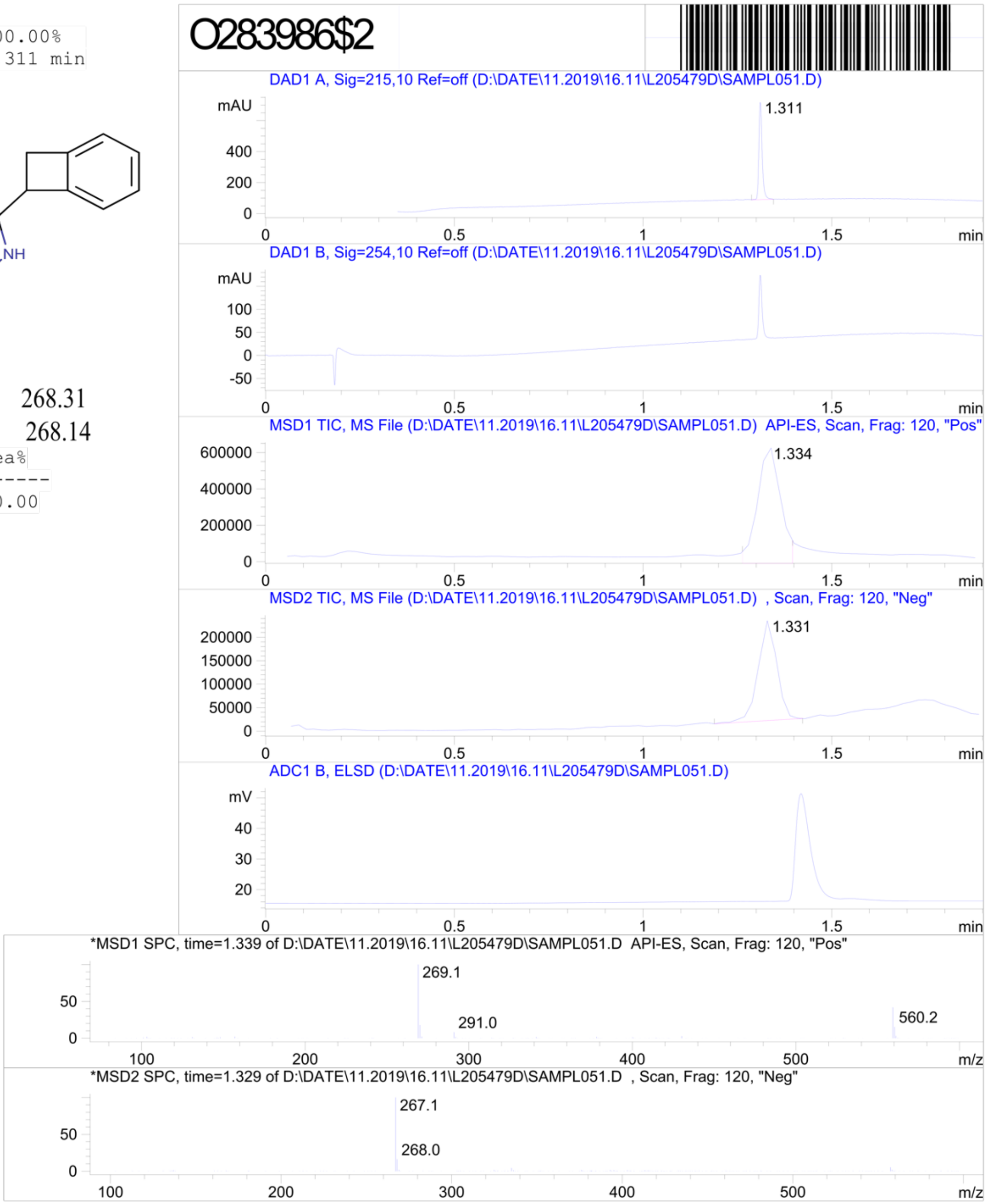

RT 1.334

RT 1.331

Inj.Date 11/16/2019 AK P2-F-07 -SL- Acq. Method C:\HPCHEM\-> ->

LCMS of **Compound 6**

MaxPeak: 100.00%
Ret_Time: 1.281 min

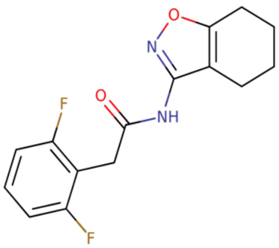

Mol Wt 292.28
Exact Mass 292.12

#	Time	Area%
1	1.281	100.00

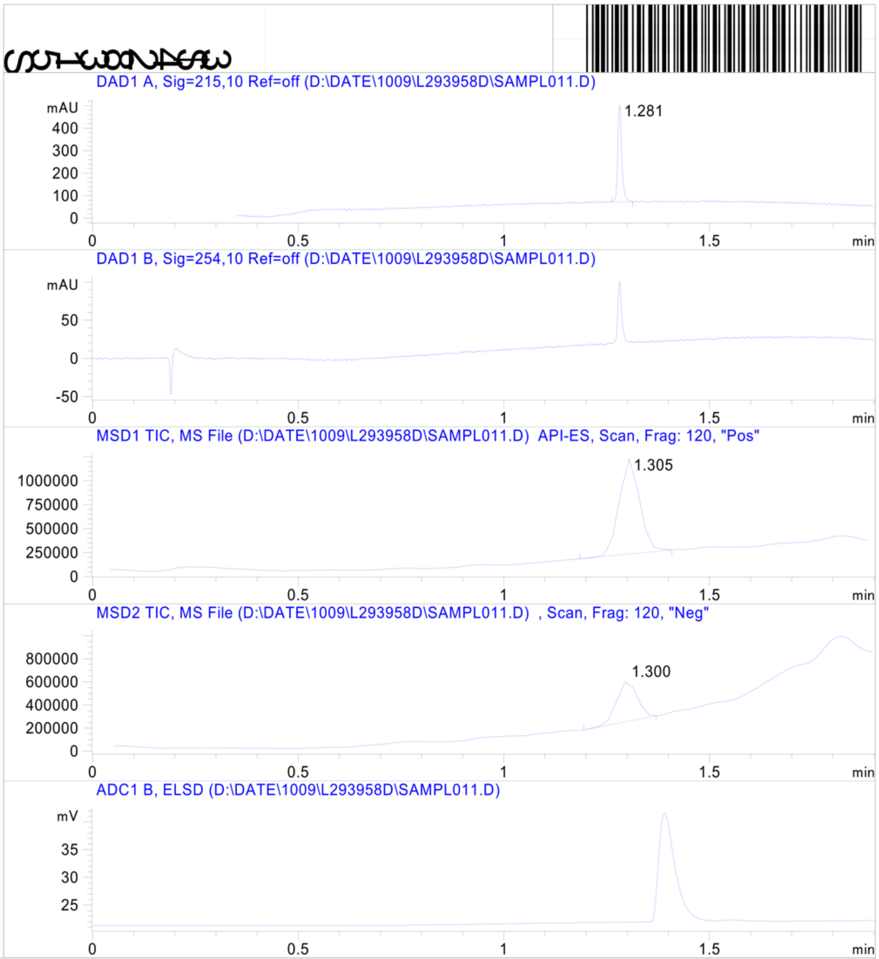

RT 1.305

RT 1.300

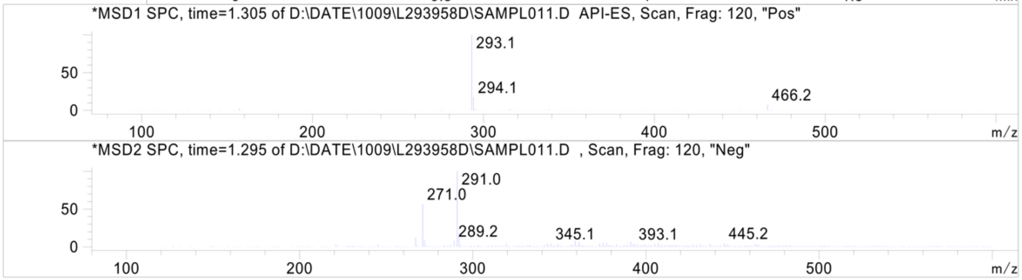

Inj.Date 10/9/2020 E -SL- Acq. Method C:\HPCHEM\-> ->

LCMS of **Compound 7**

MaxPeak: 100.00%
Ret_Time: 1.273 min

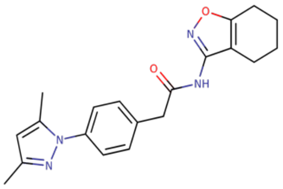

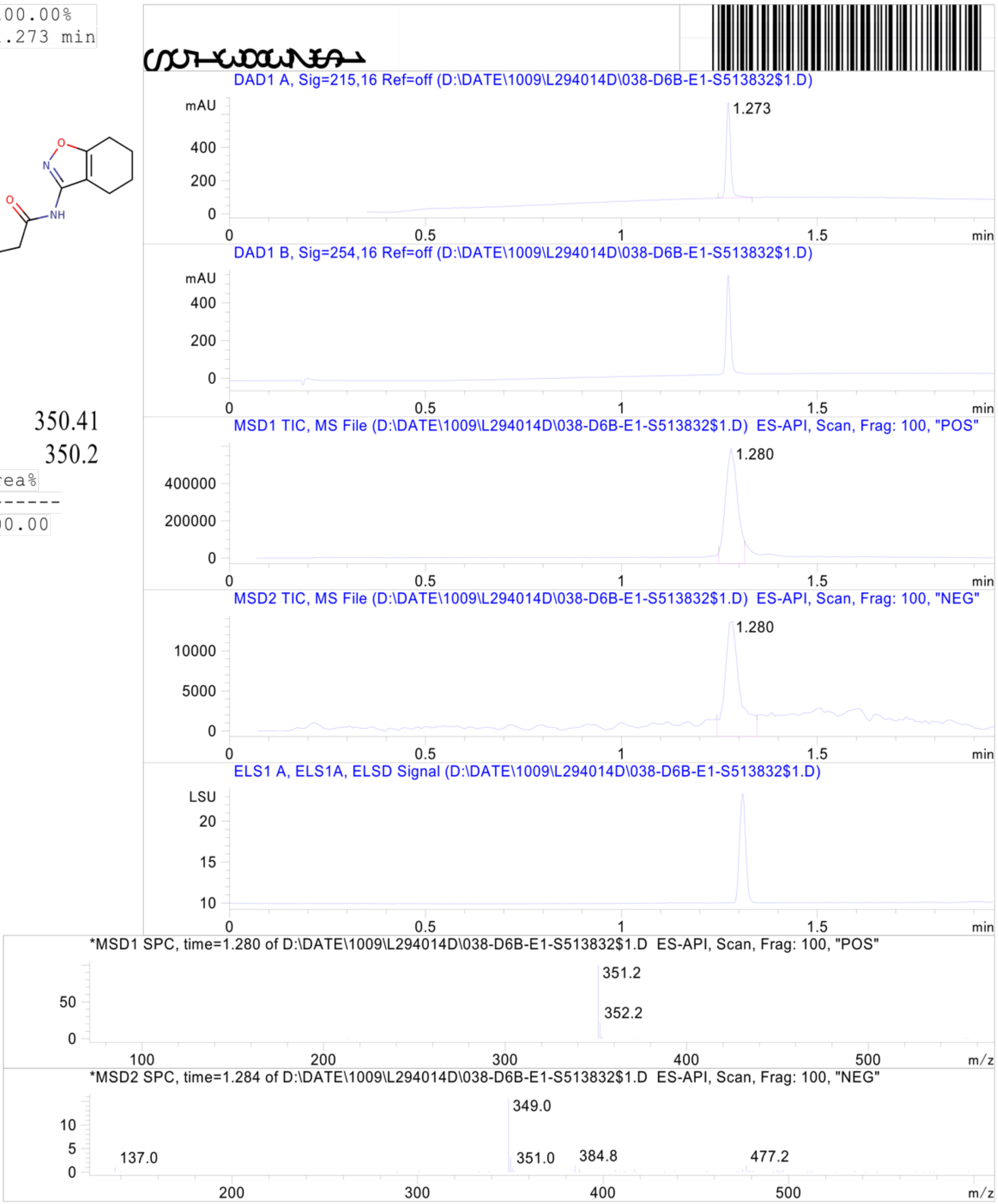

Mol Wt 350.41
Exact Mass 350.2

#	Time	Area%
1	1.273	100.00

RT 1.280

RT 1.280

Inj.Date 10/8/2020 E -15- Acq. Method C:\Chem32\-> ->

LCMS of **Compound 8**

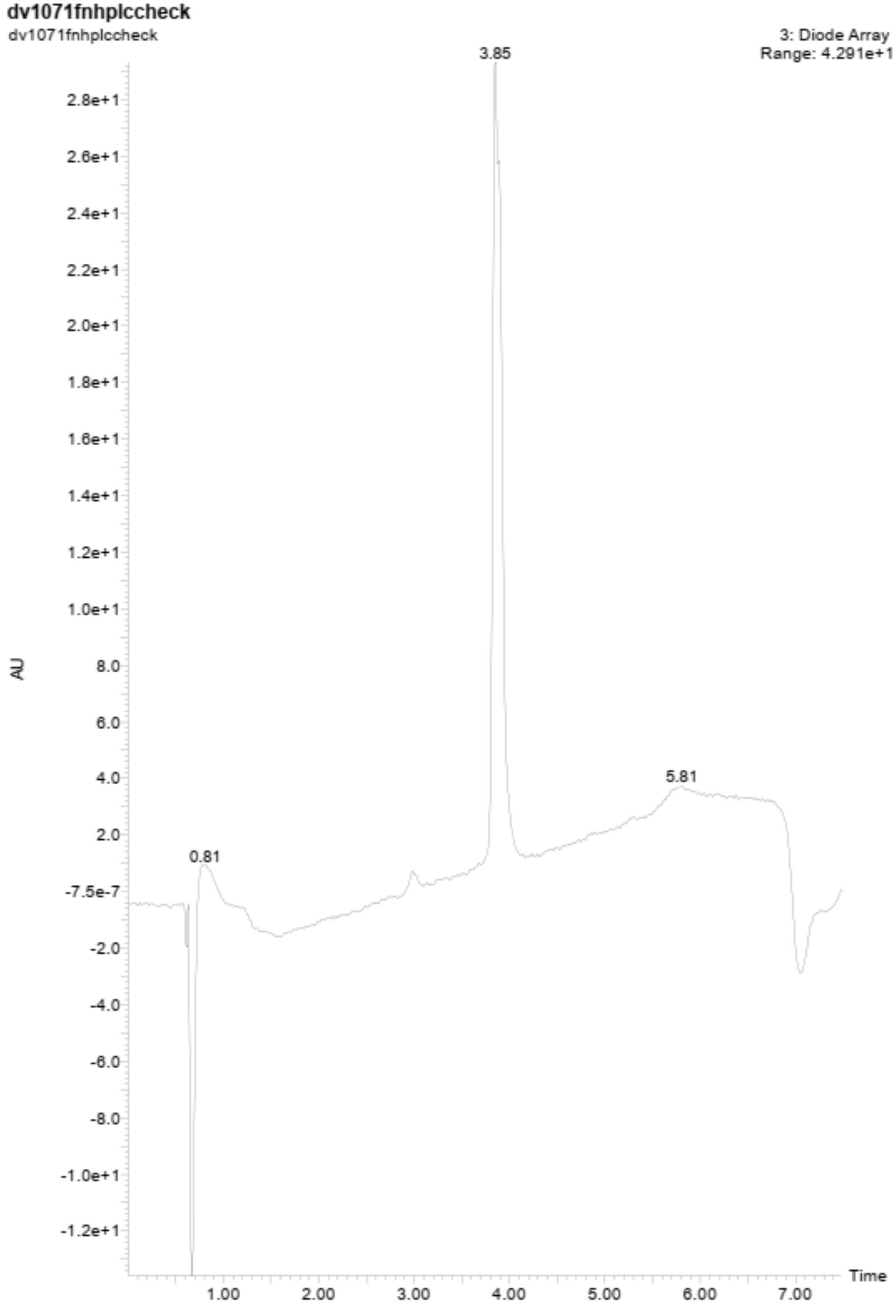

LCMS of **Compound 9**

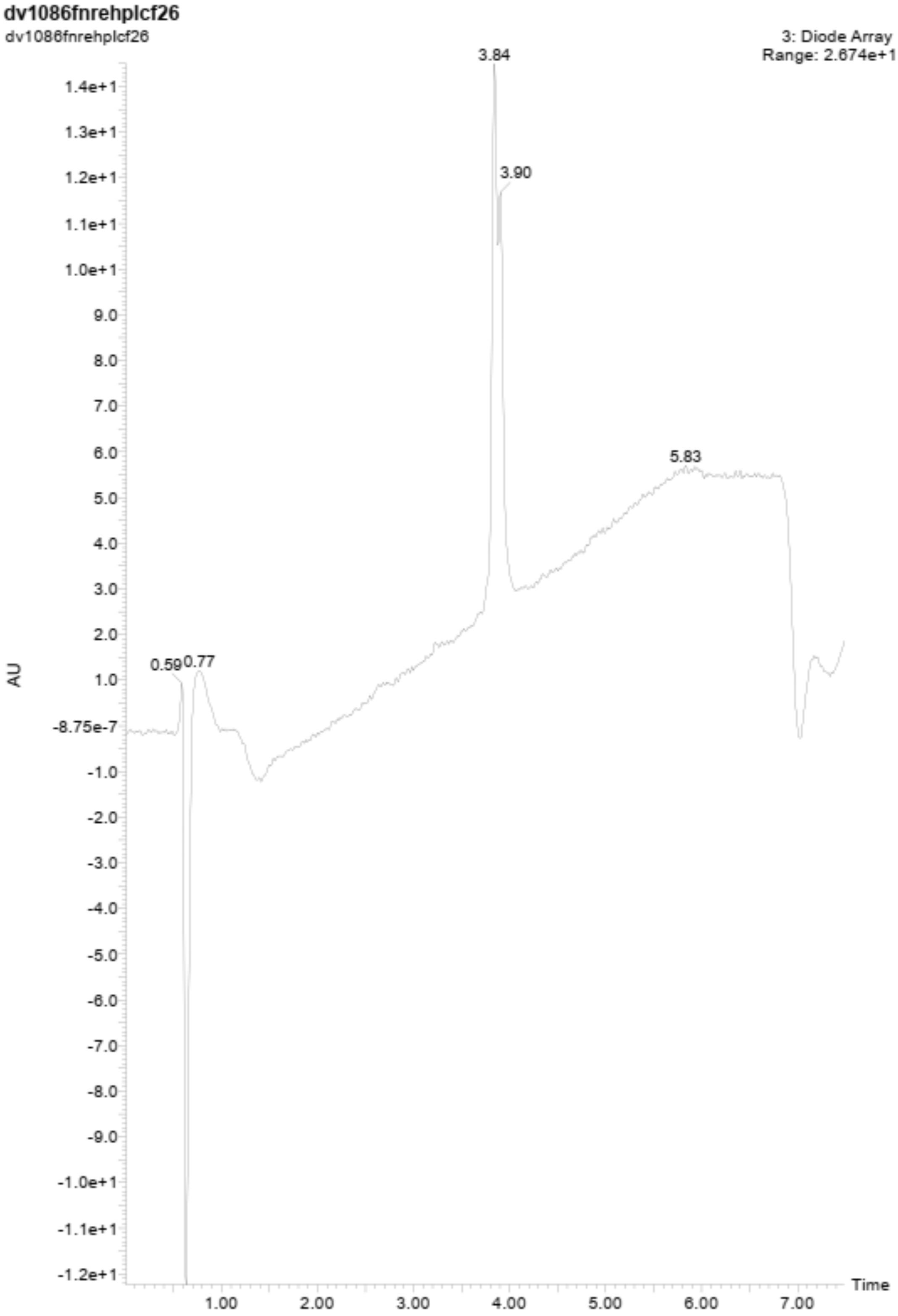

LCMS of **Compound 10**

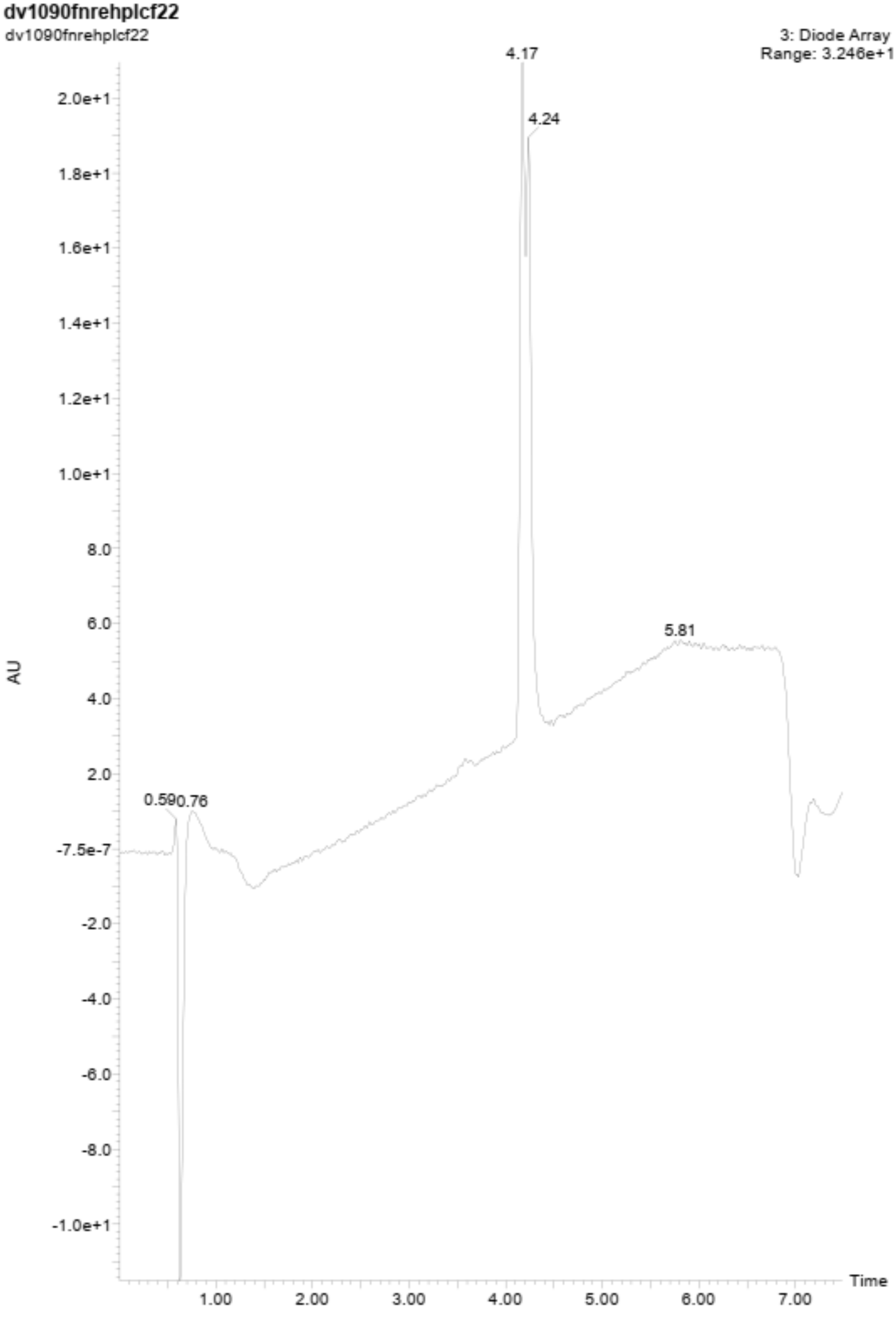

LCMS of **ZINC900776257**

MaxPeak: 90.54%
Ret_Time: 1.120 min

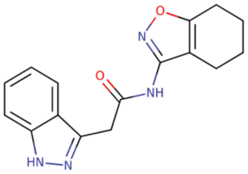

Mol Wt 296.32
Exact Mass 296.14

#	Time	Area%
1	0.836	3.39
2	0.986	2.01
3	1.120	90.54
4	1.161	4.06

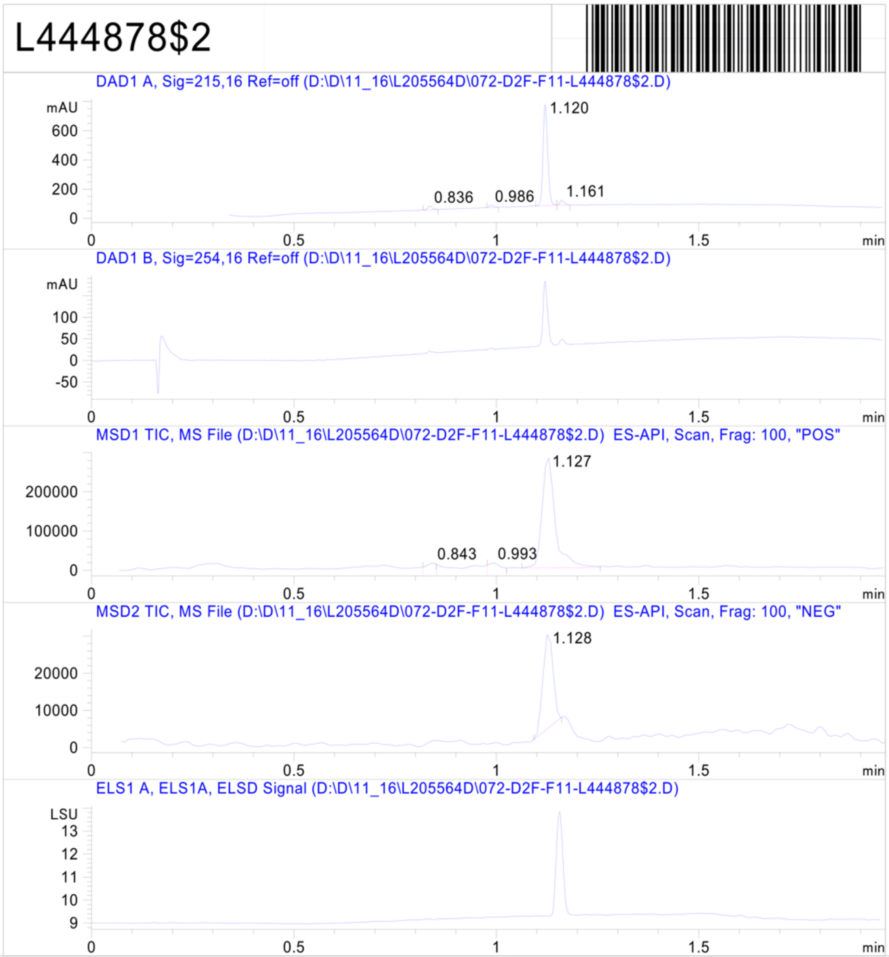

RT 0.843

RT 0.993

RT 1.127

RT 1.128

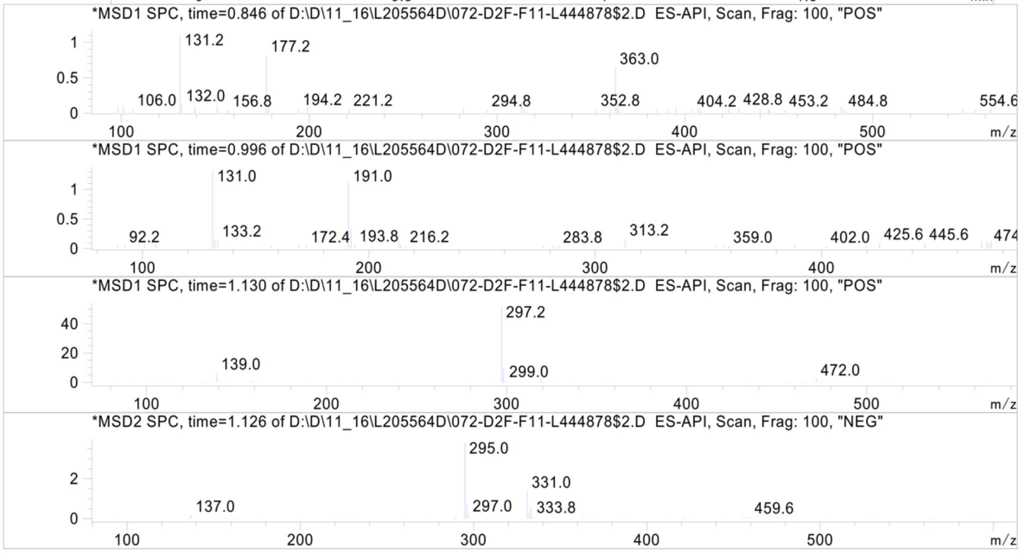

Inj.Date 11/16/2019 N <invalid> -17- Acq. Method C:\Chem32\-> ->

LCMS of **Compound 11**

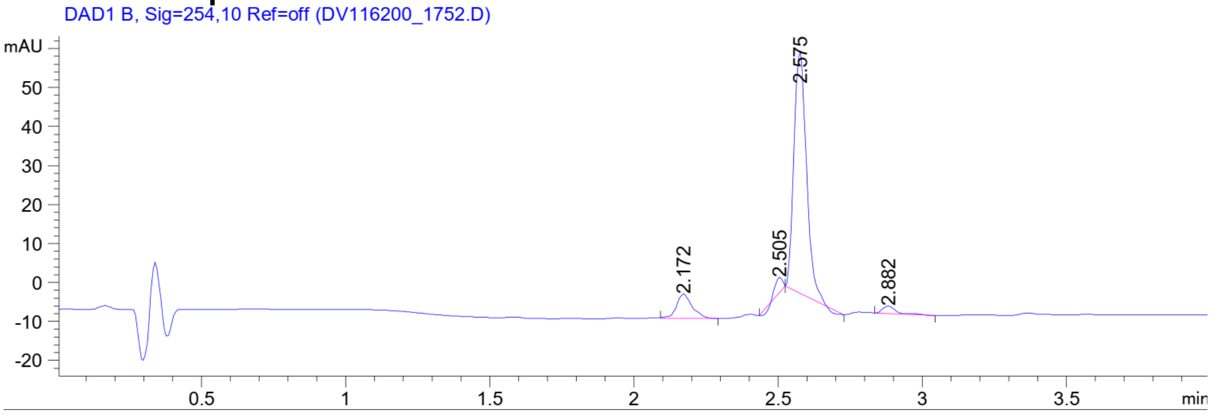

LCMS of **Compound 12**

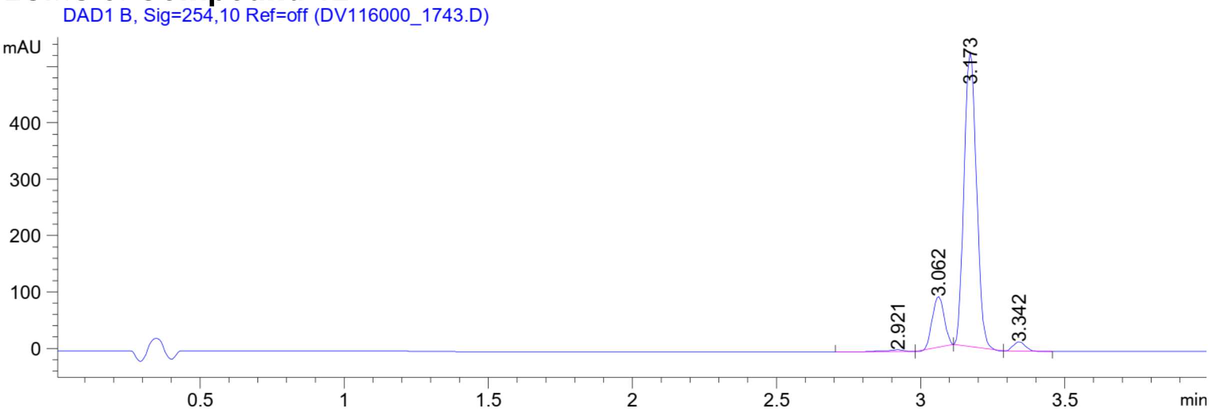

LCMS of **Compound 13**

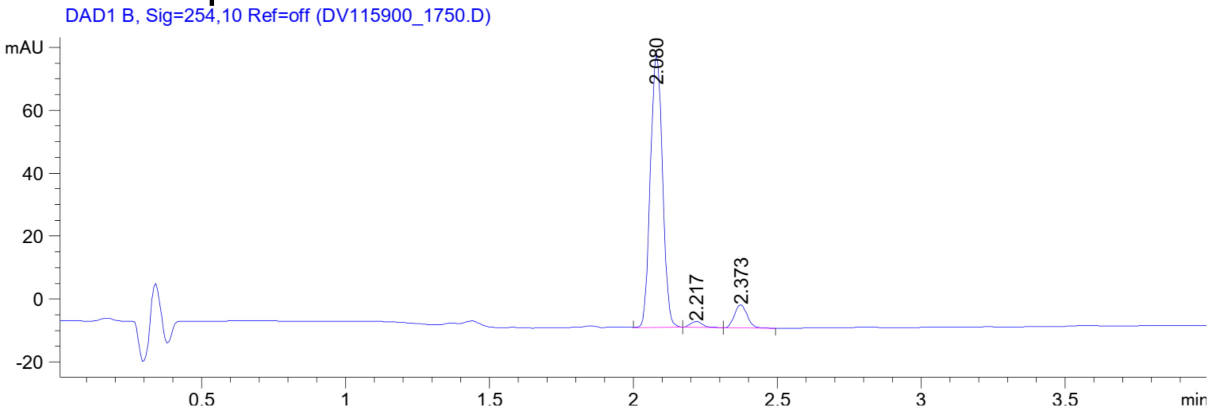

LCMS of **Compound 14**

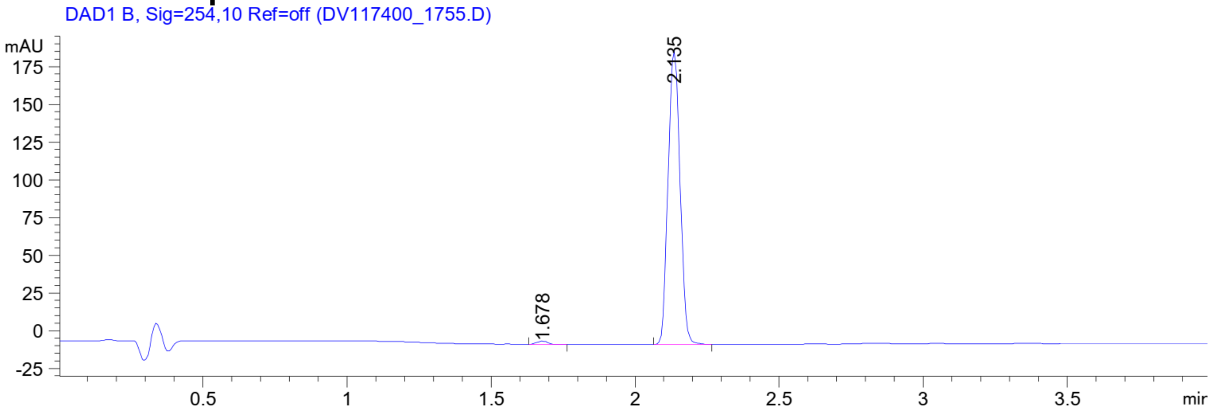

LCMS of **Compound 15**

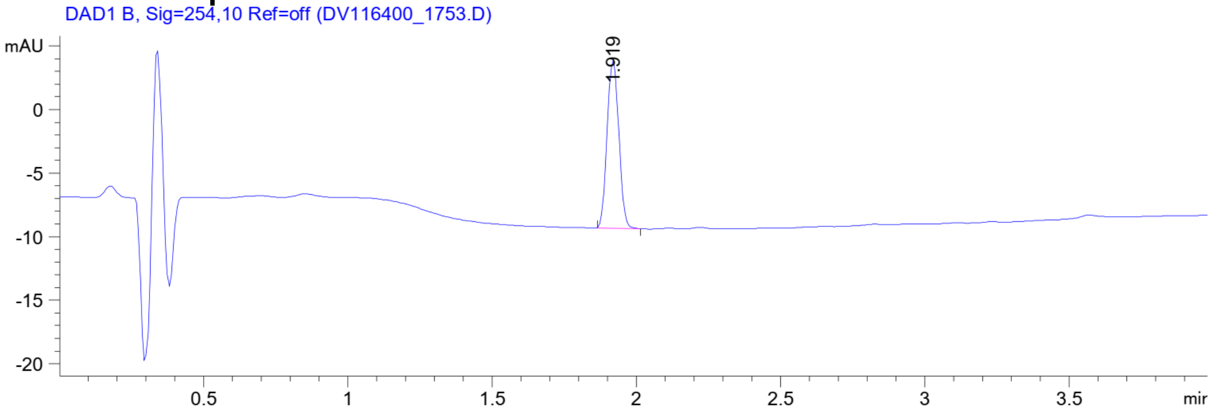

LCMS of **Compound 16**

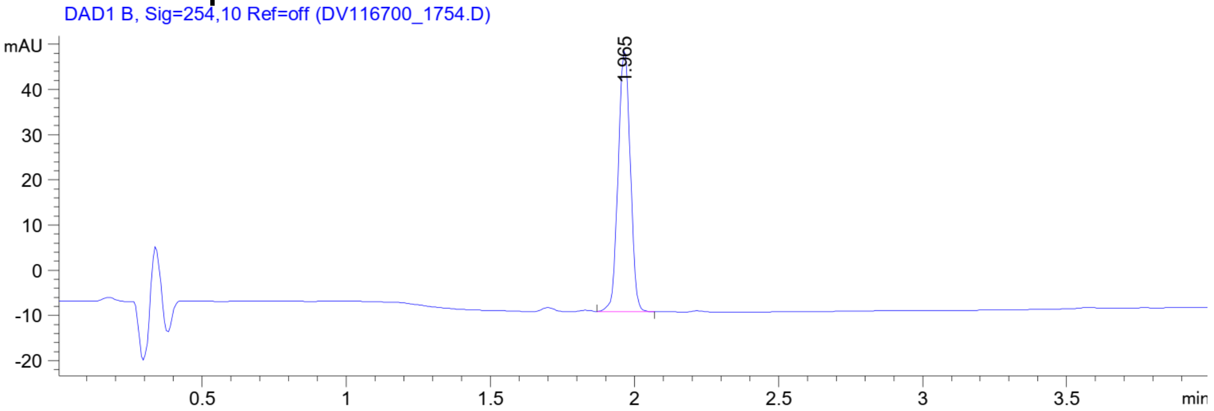

LCMS of **Compound 18**

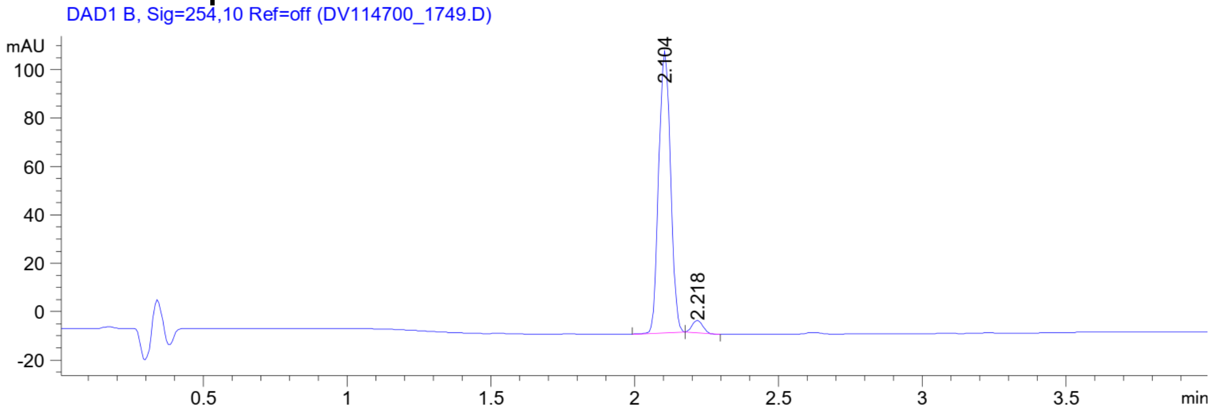

LCMS of **Compound 21**

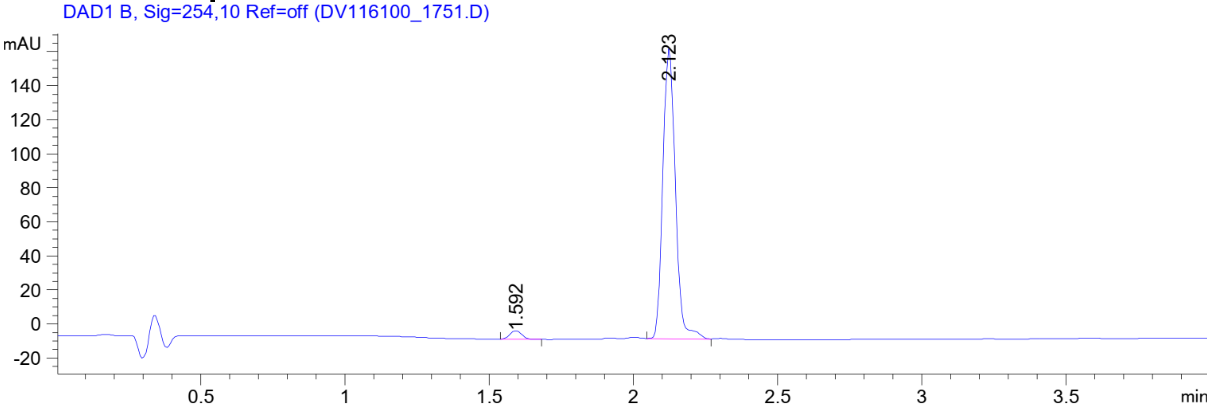

LCMS of **Compound 22**

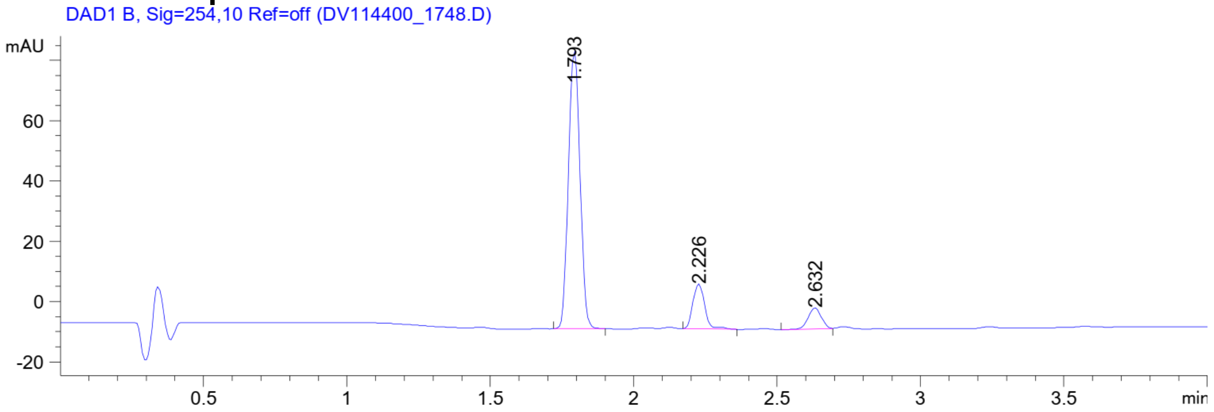

LCMS of **Compound 17**

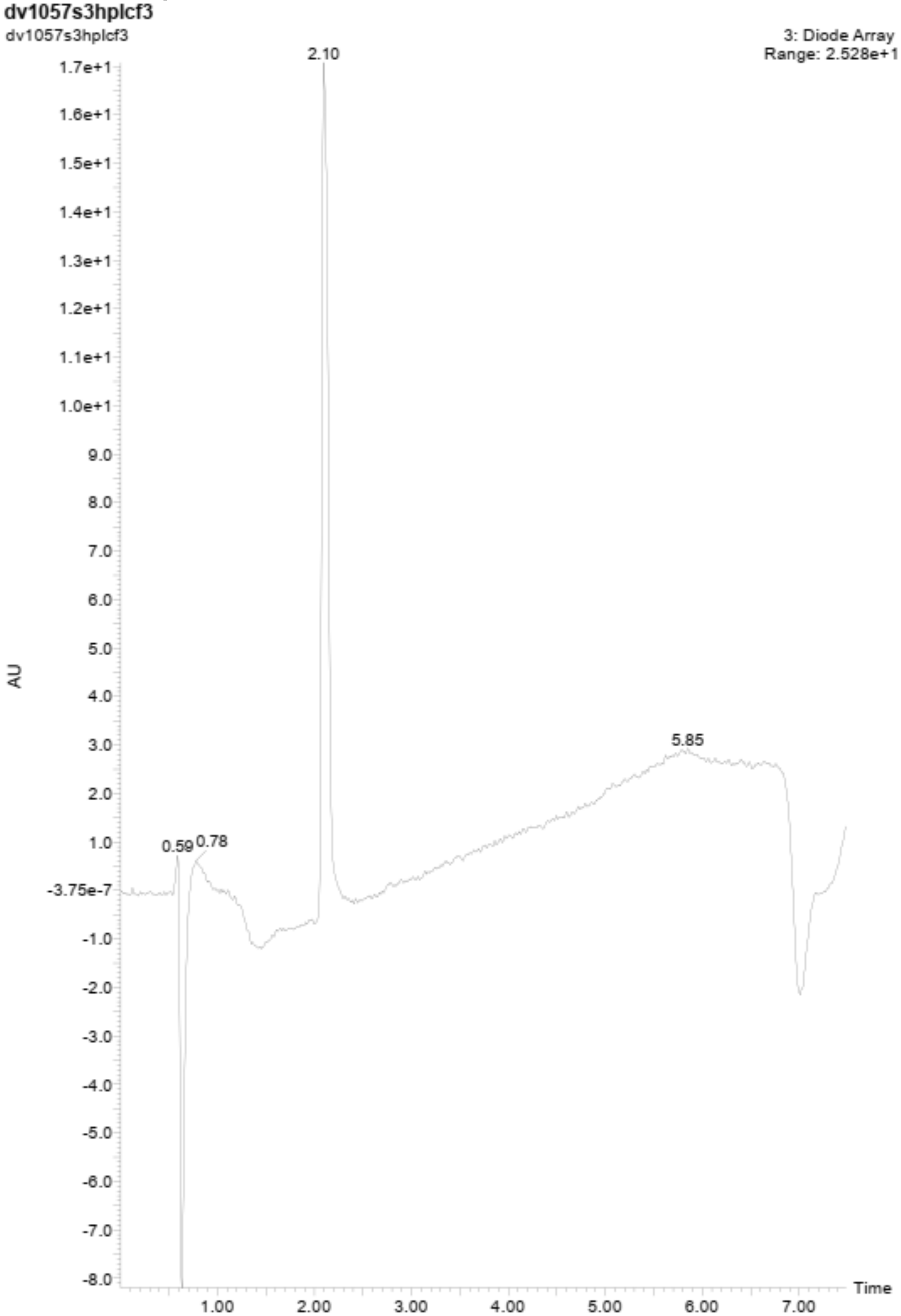

LCMS of **Compound 19**

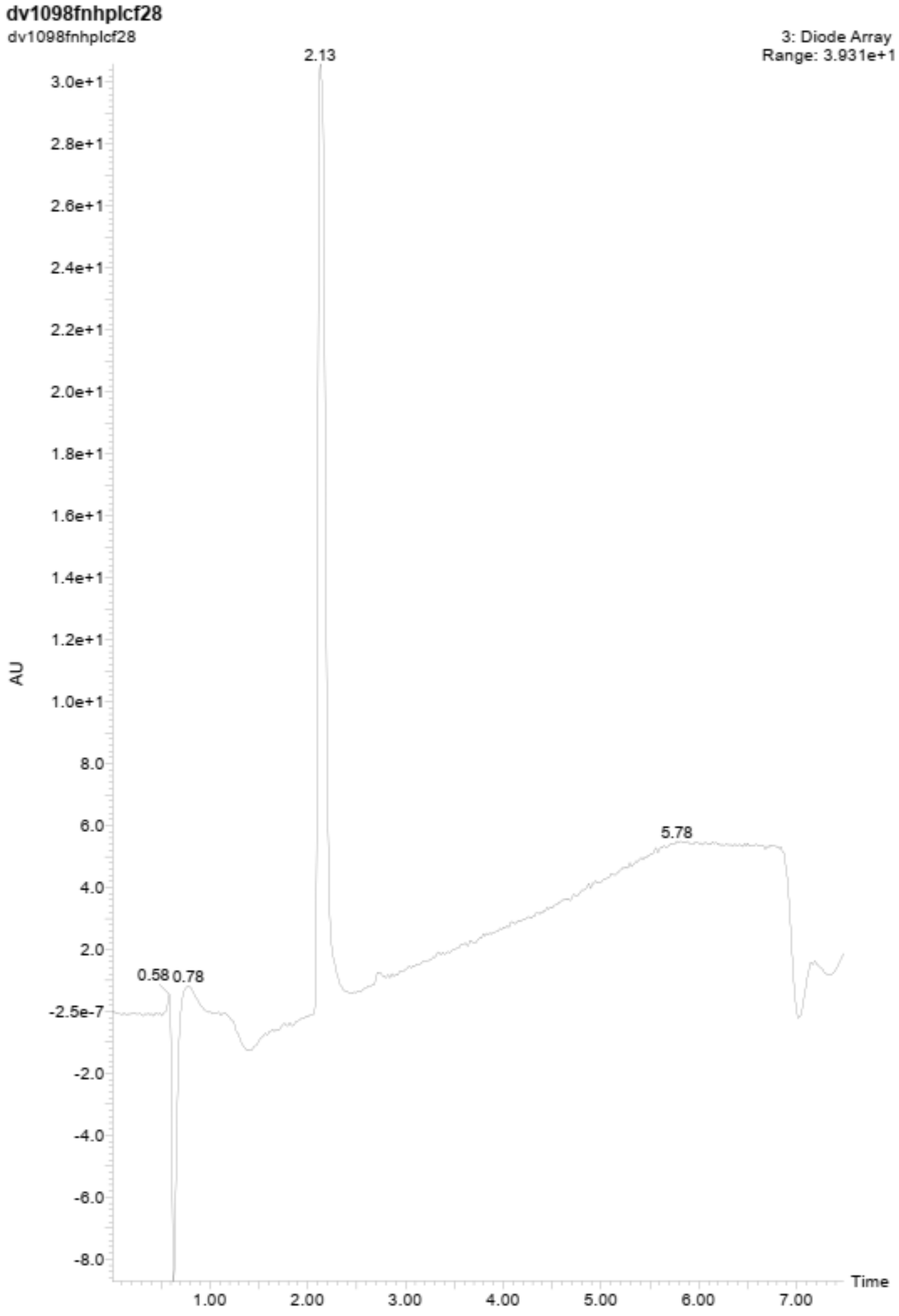

LCMS of **Compound 20**

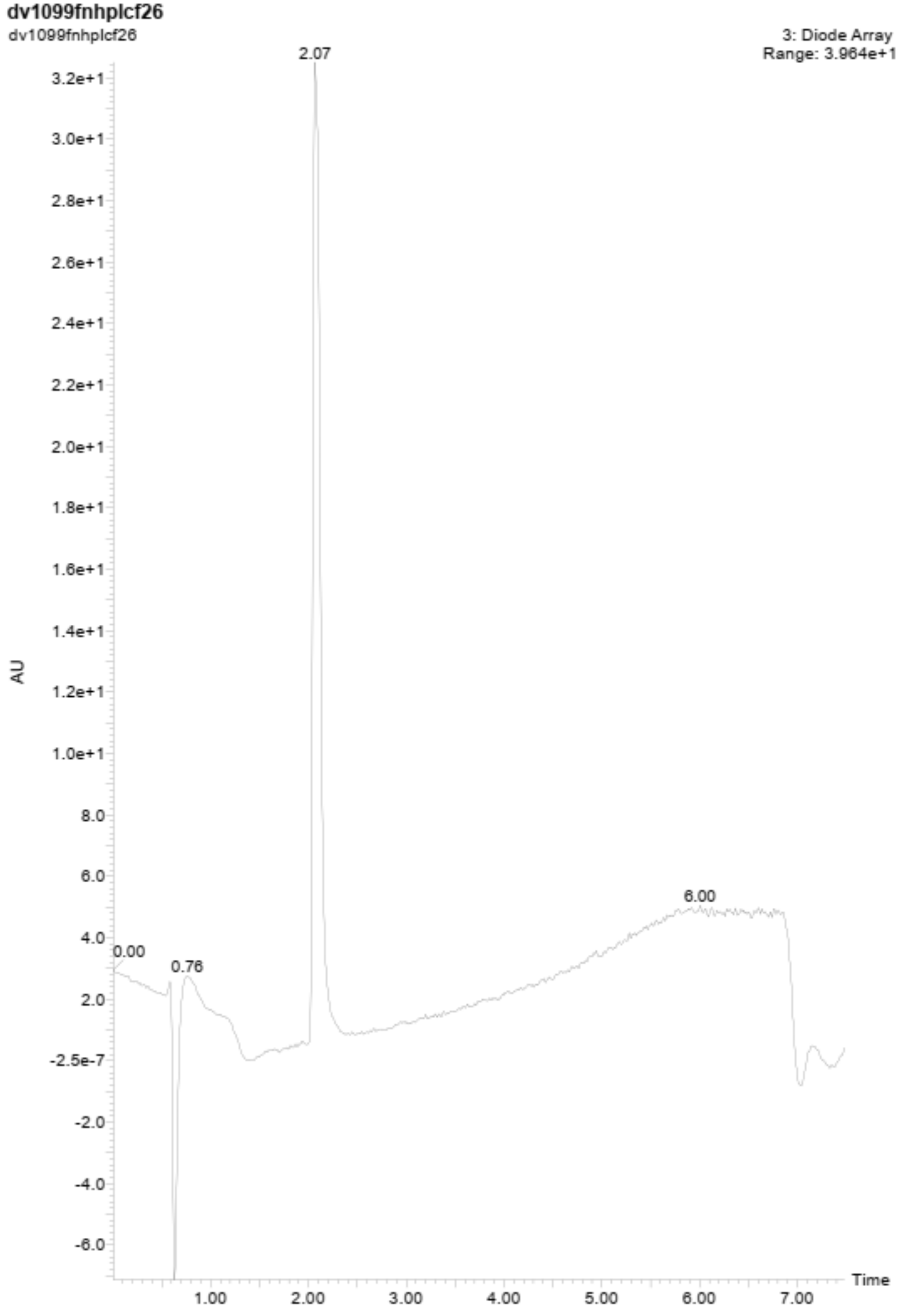

LCMS of **Compound 23**

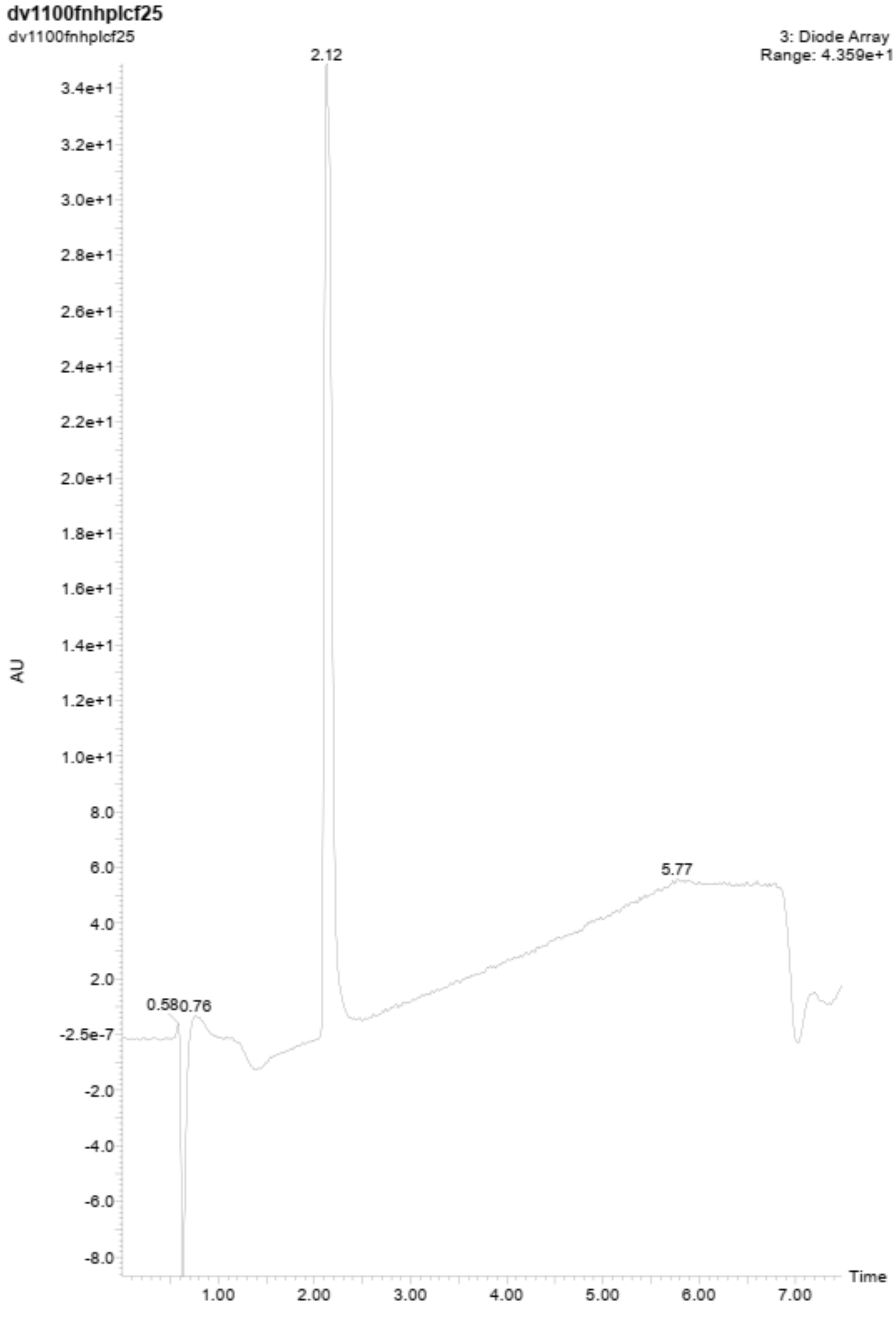

NMR Spectral Data

^1H NMR of **Compound 21** (600 MHz, CD_3OD)

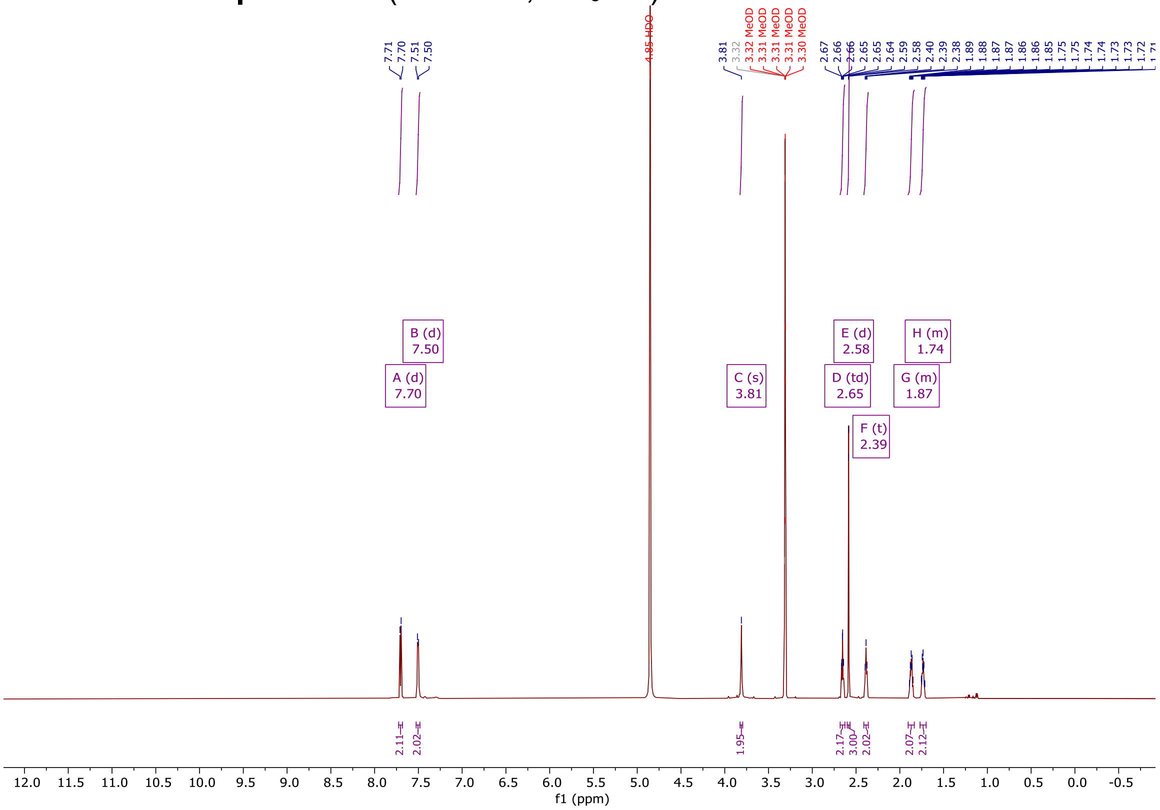

^{13}C NMR of **Compound 21** (150 MHz, CD_3OD)

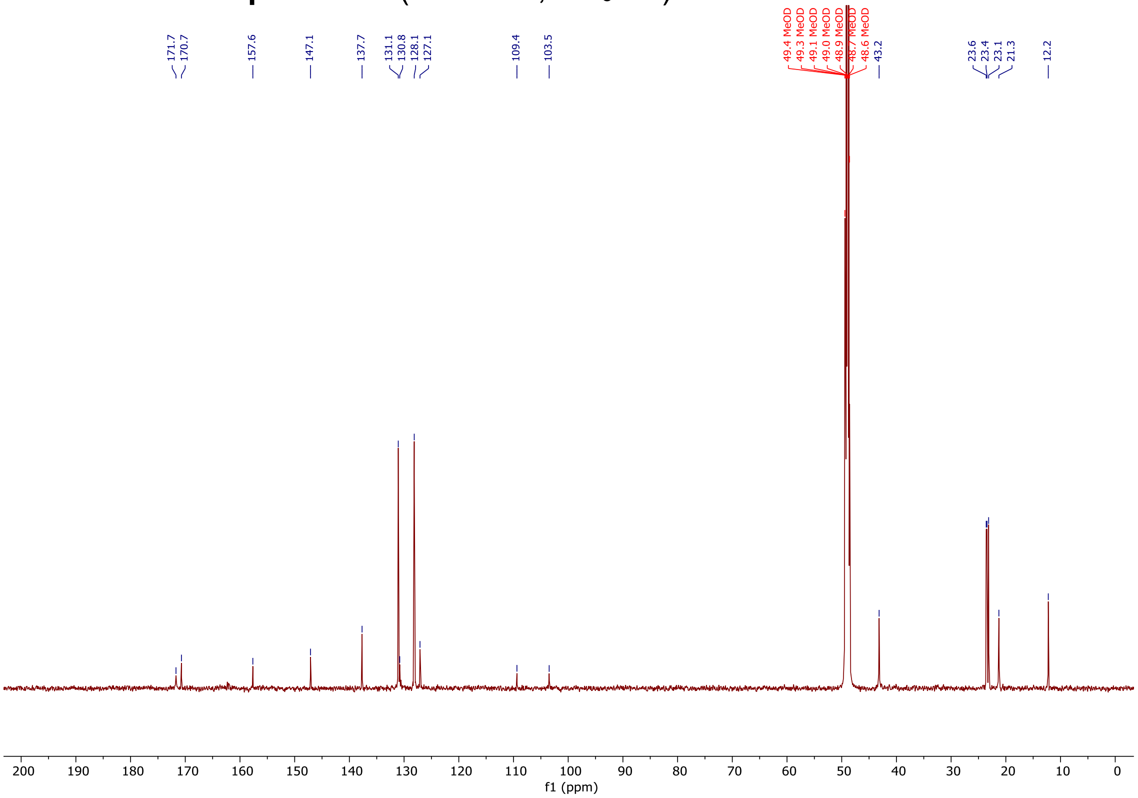

^{1}H NMR of **Compound 18** (600 MHz, CD_3OD)

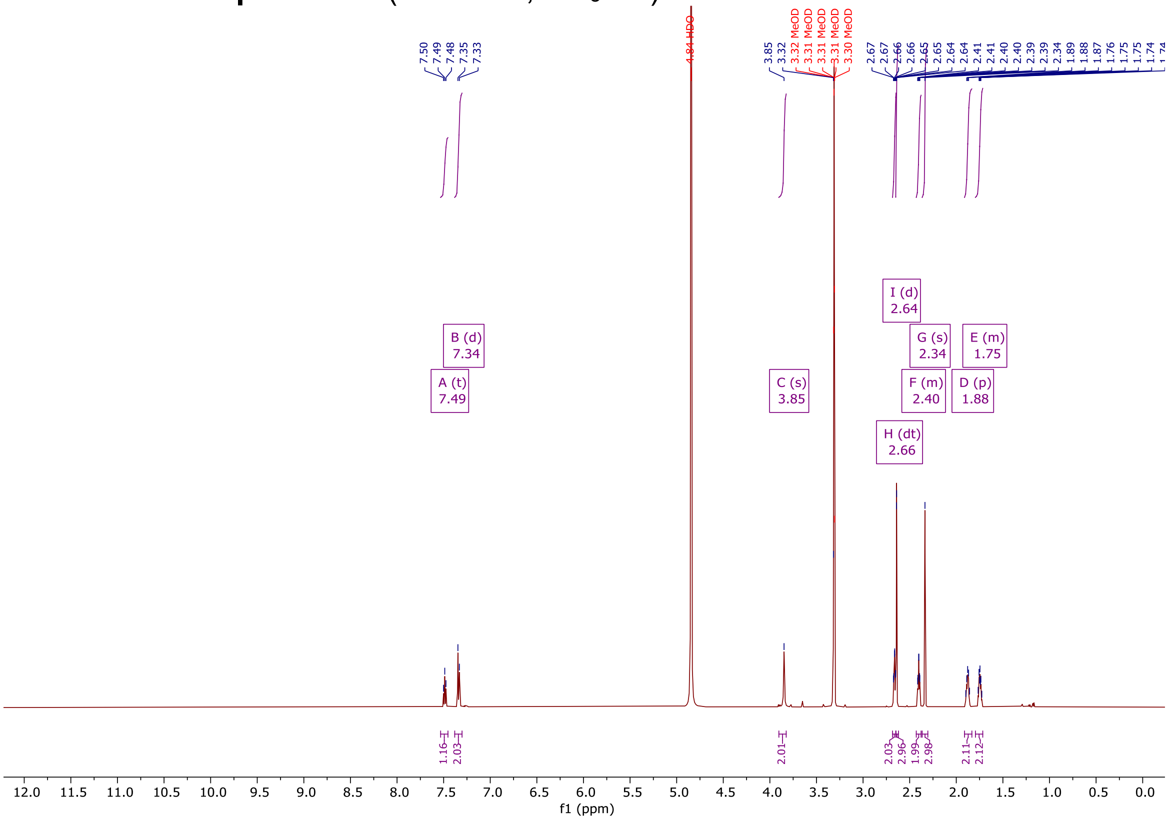

^{13}C NMR of **Compound 18** (150 MHz, CD_3OD)

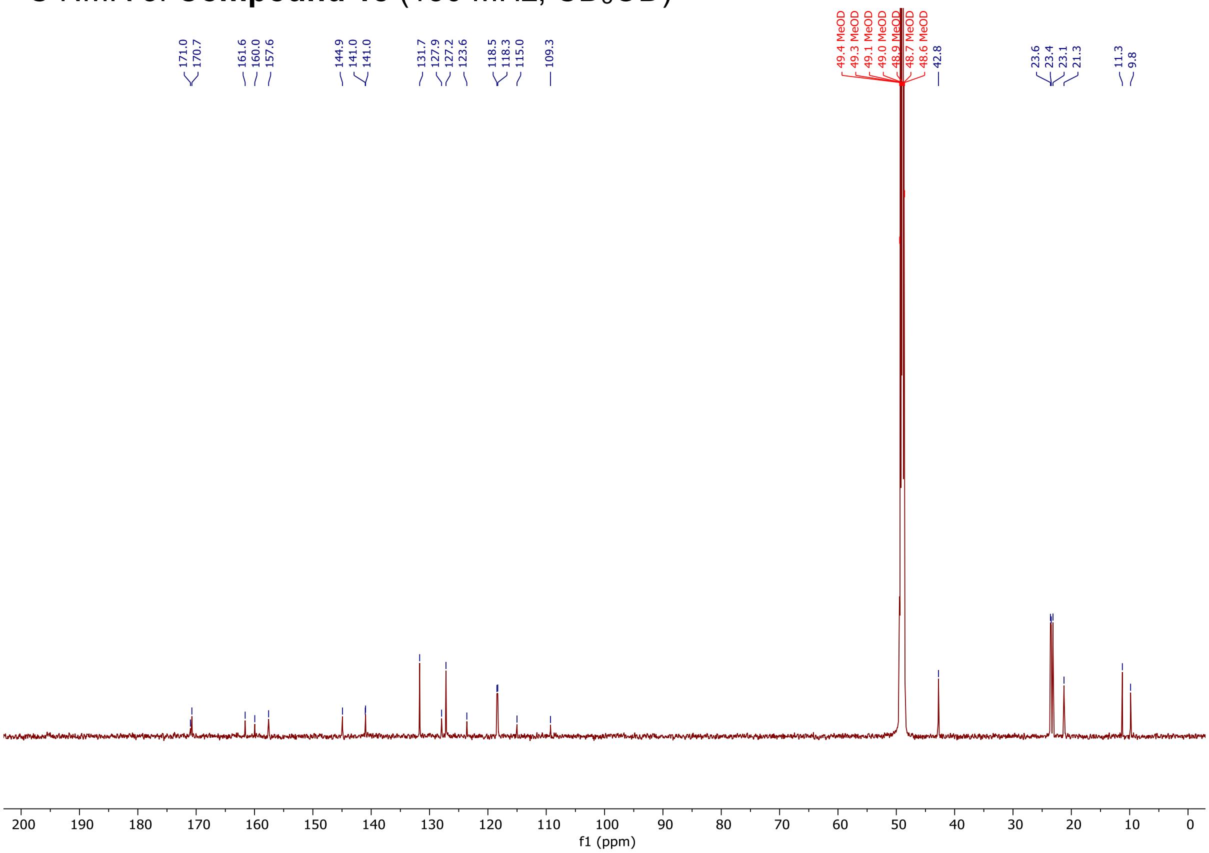

^{1}H NMR of **Compound 22** (600 MHz, CD_3OD)

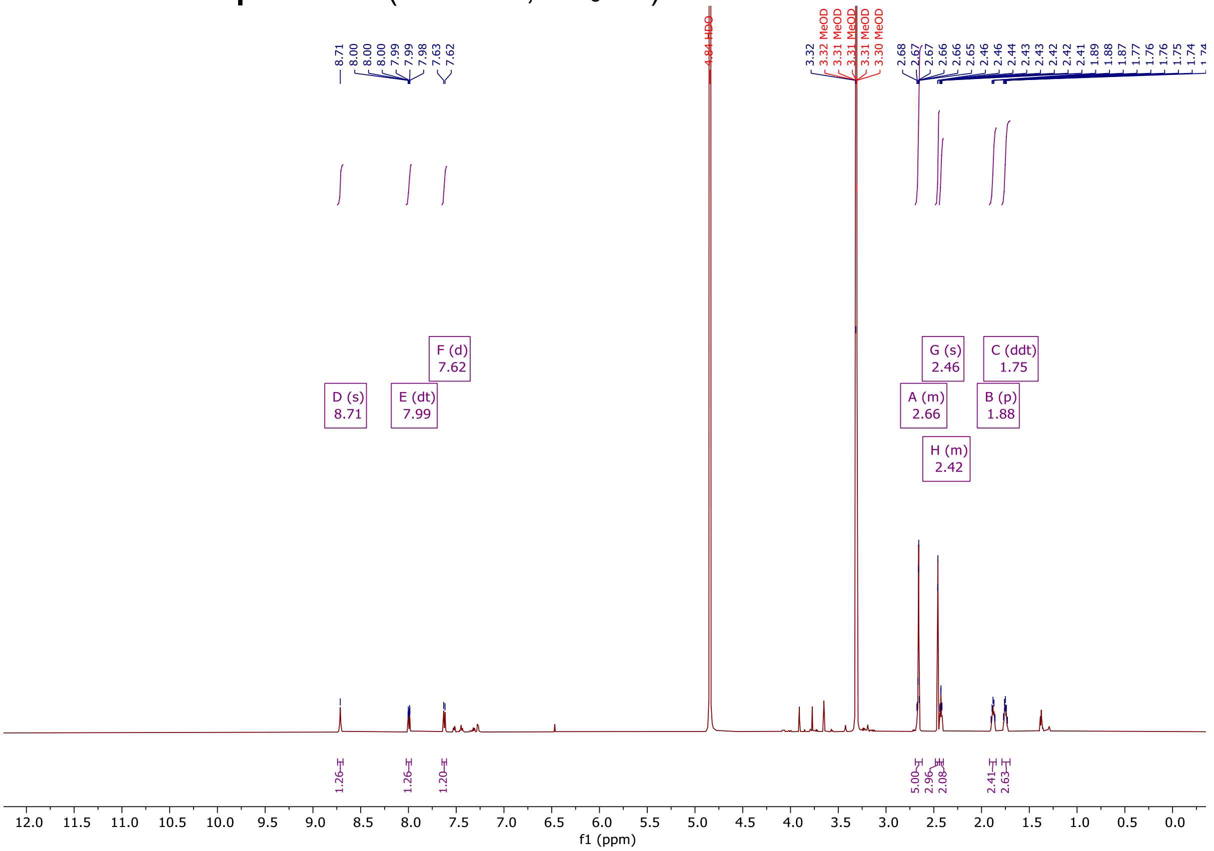

^{1}H NMR of **Compound 11** (600 MHz, CD_3OD)

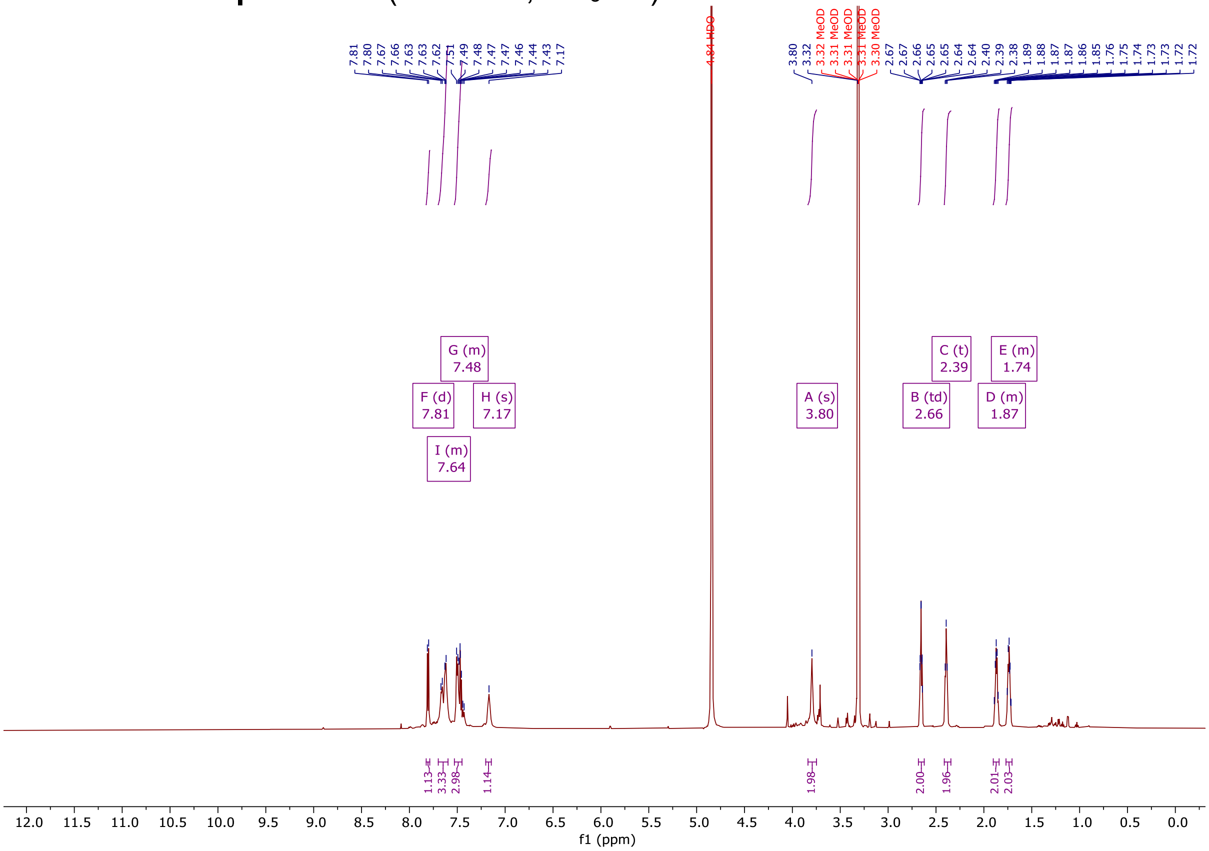

^{1}H NMR of **Compound 12** (600 MHz, CD_3OD)

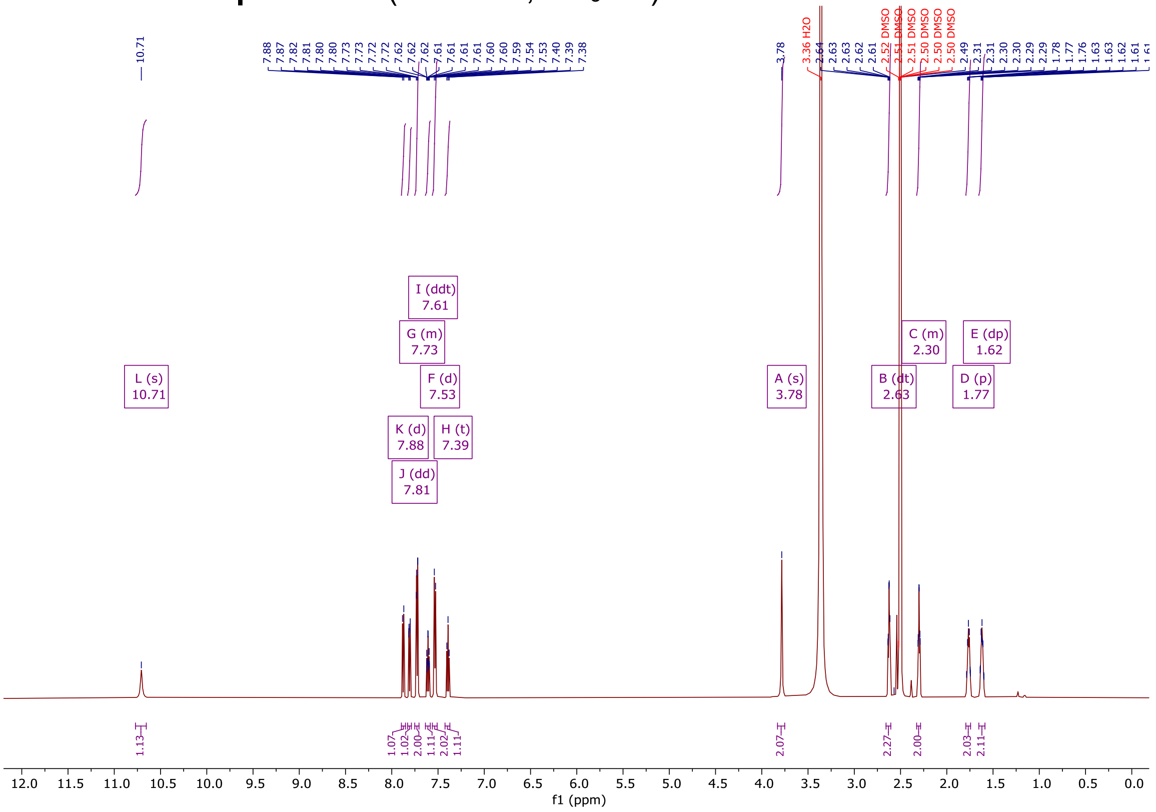

^{13}C NMR of **Compound 12** (150 MHz, CD_3OD)

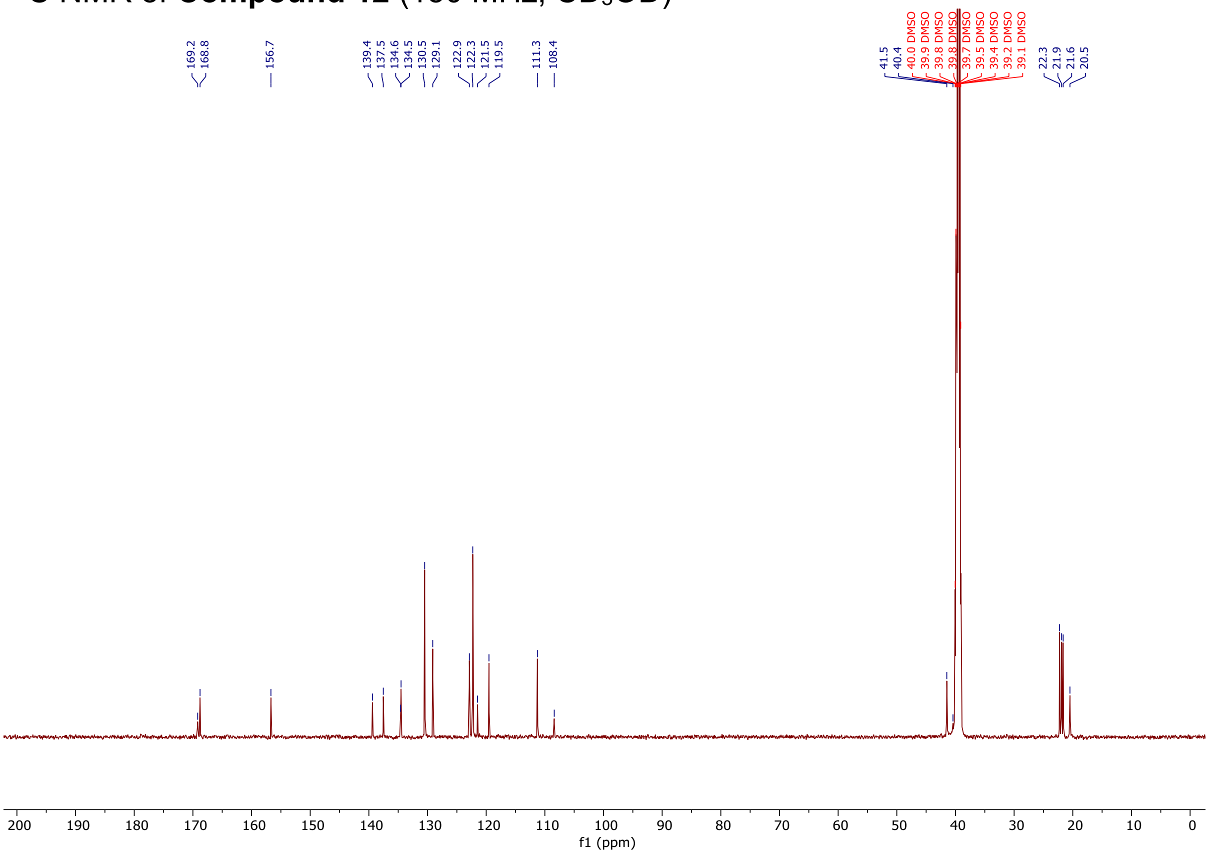

^{1}H NMR of **Compound 13** (600 MHz, CD_3OD)

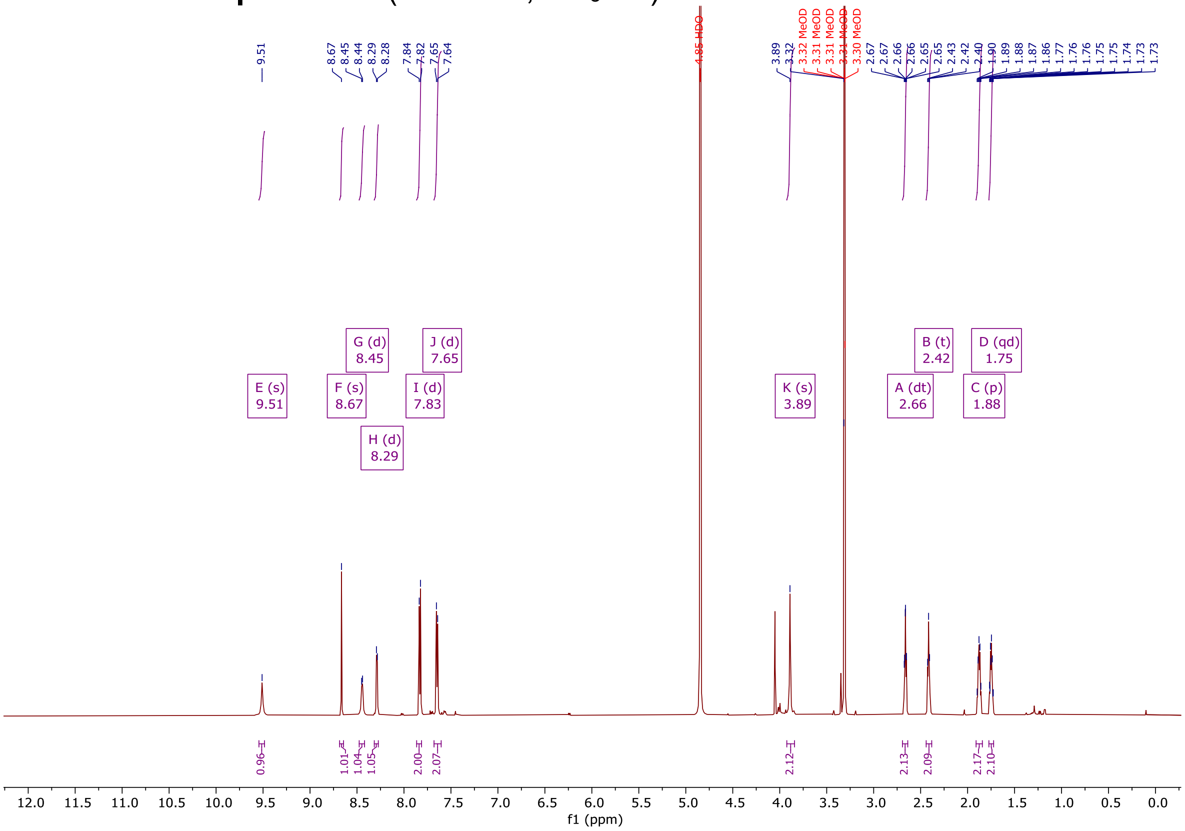

^{13}C NMR of **Compound 13** (150 MHz, CD_3OD)

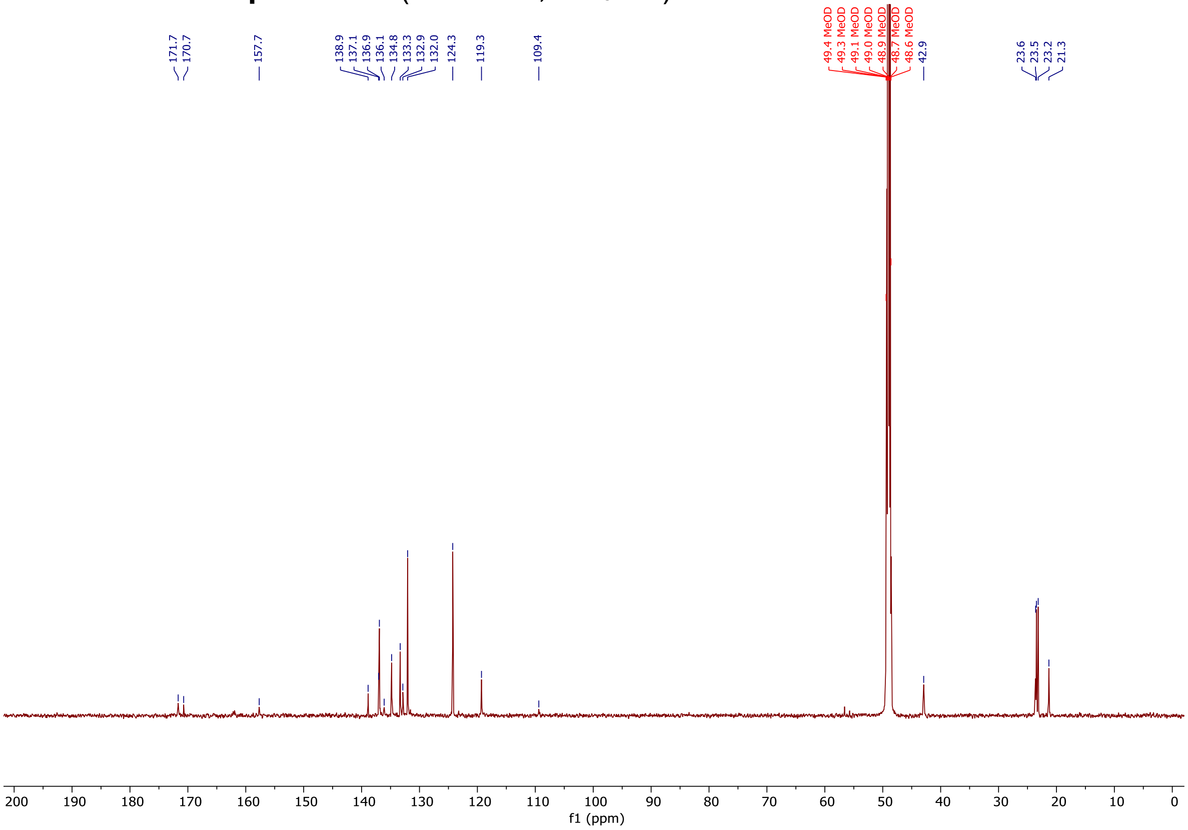

^{1}H NMR of **Compound 15** (600 MHz, CD_3OD)

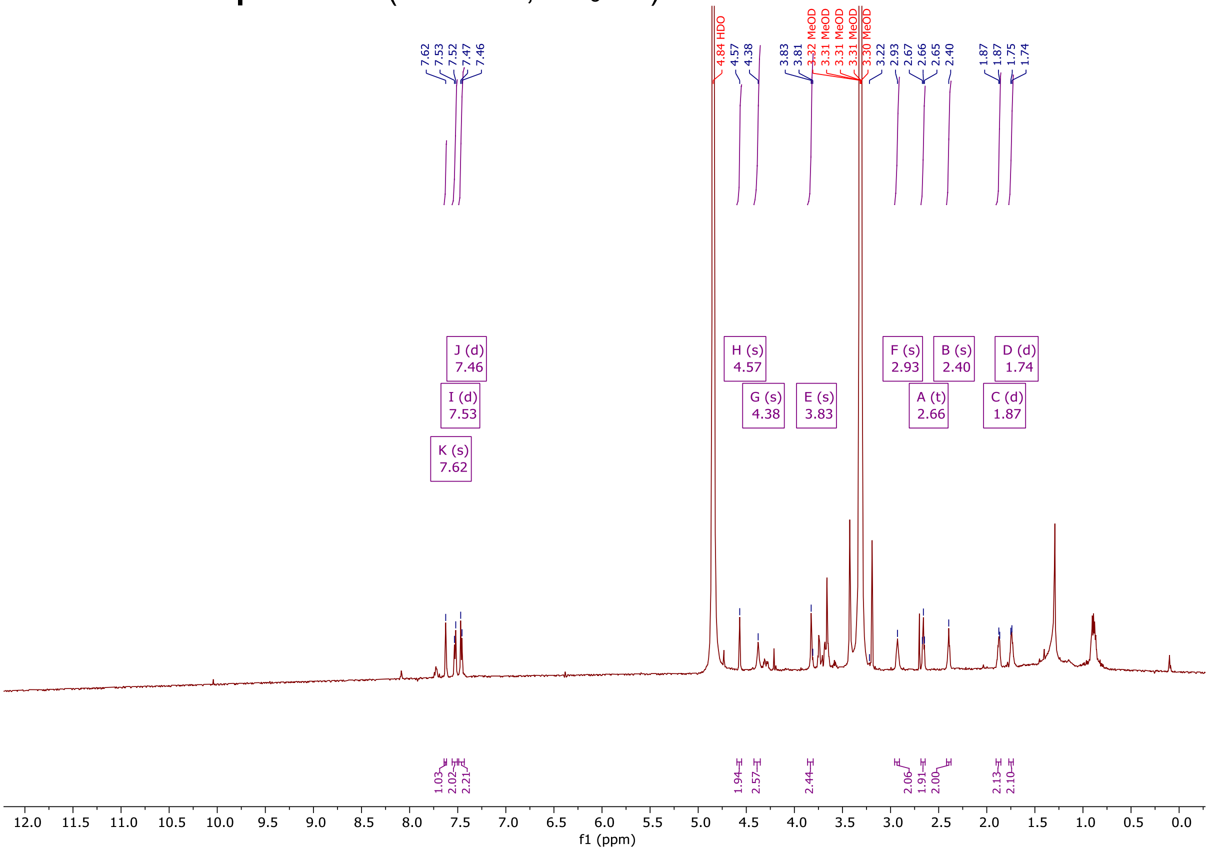

^{1}H NMR of **Compound 16** (600 MHz, CD_3OD)

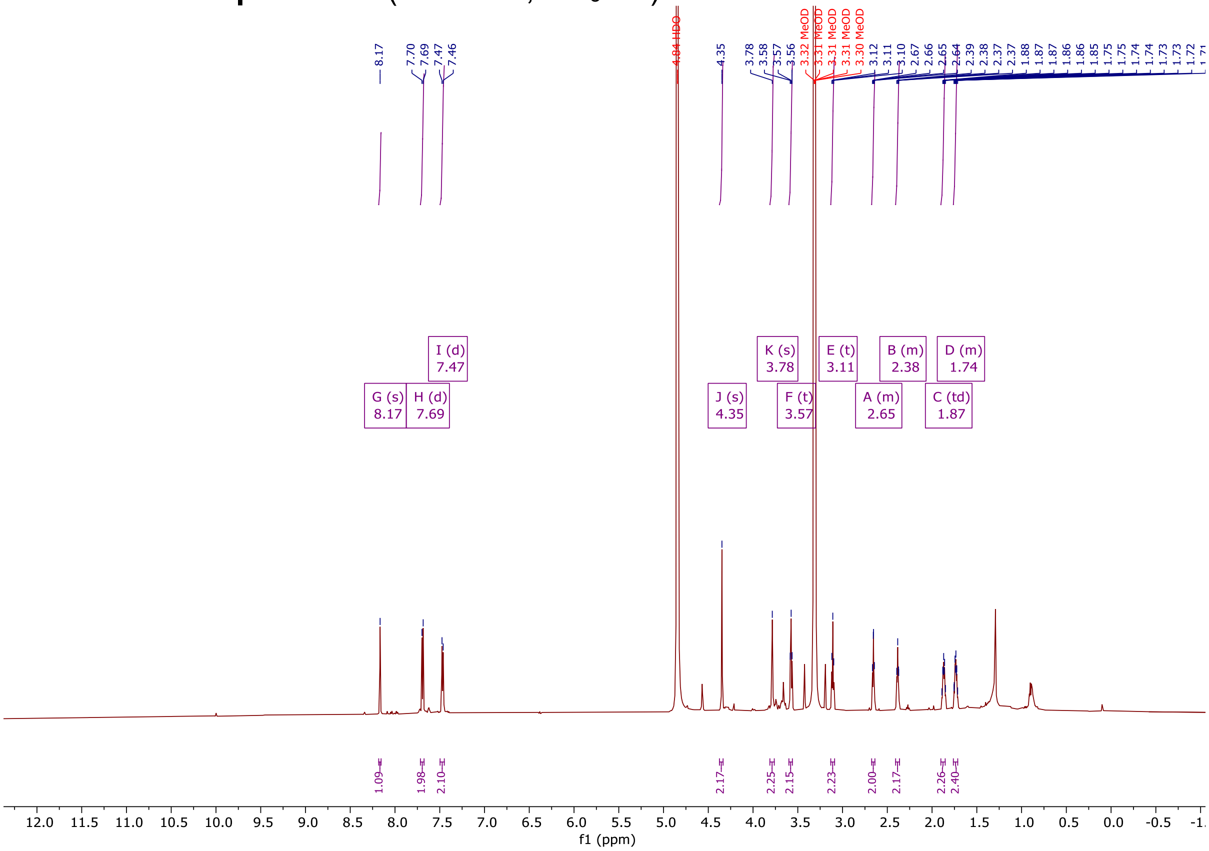

^{1}H NMR of **Compound 14** (600 MHz, CD_3OD)

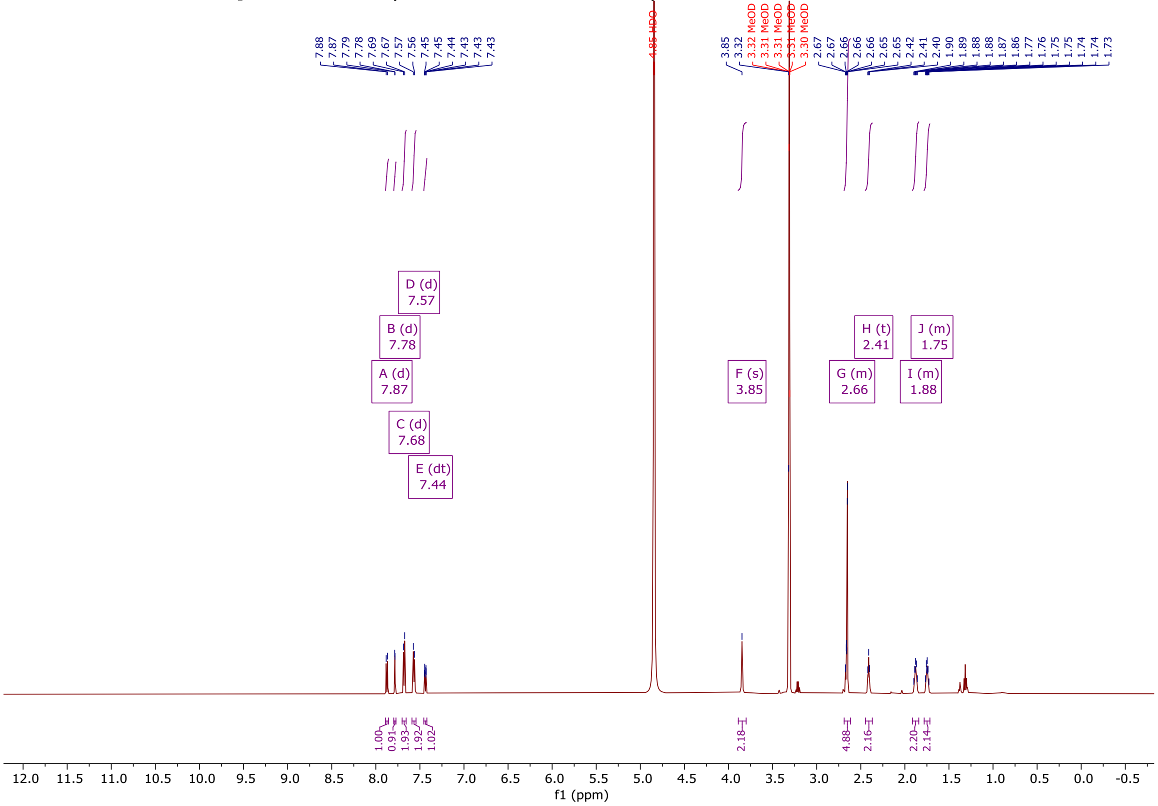

^{13}C NMR of **Compound 14** (150 MHz, CD_3OD)

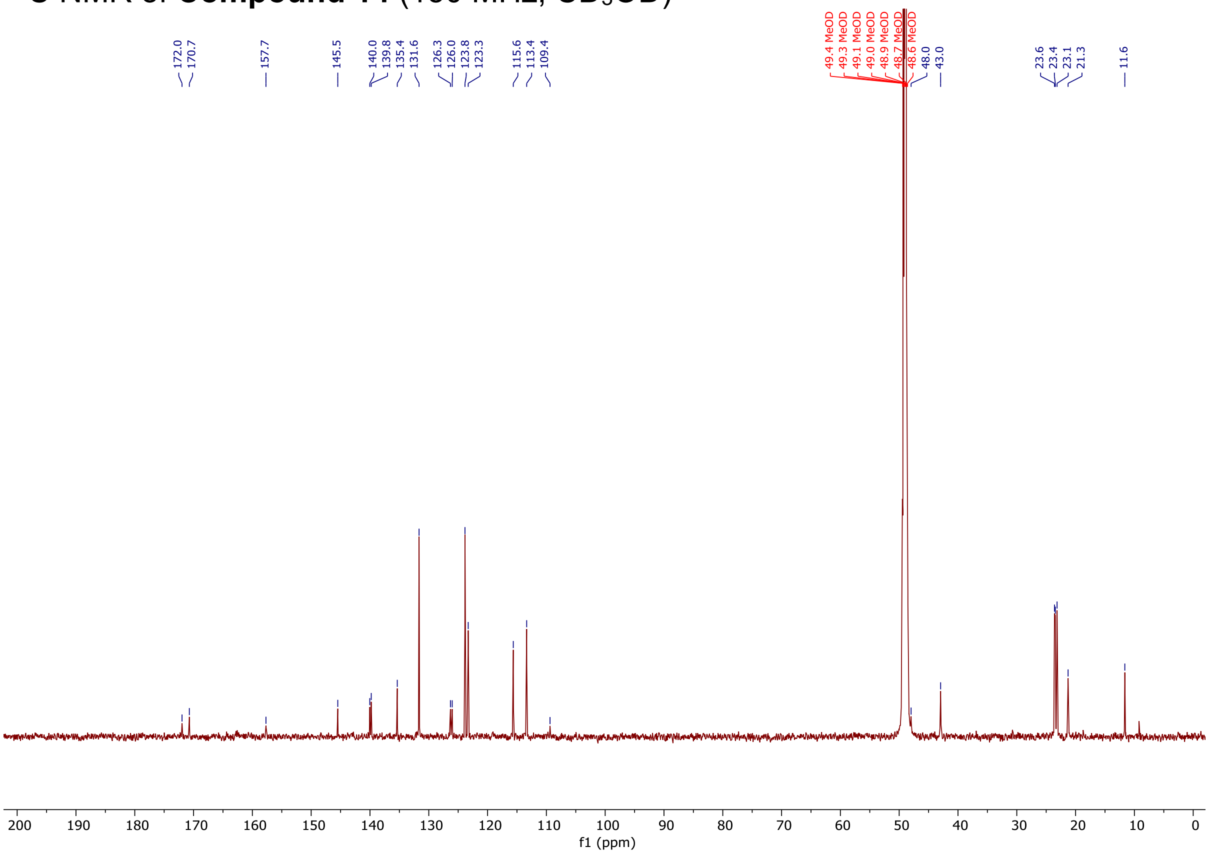

^1H NMR of **Compound 23** (600 MHz, CD_3OD)

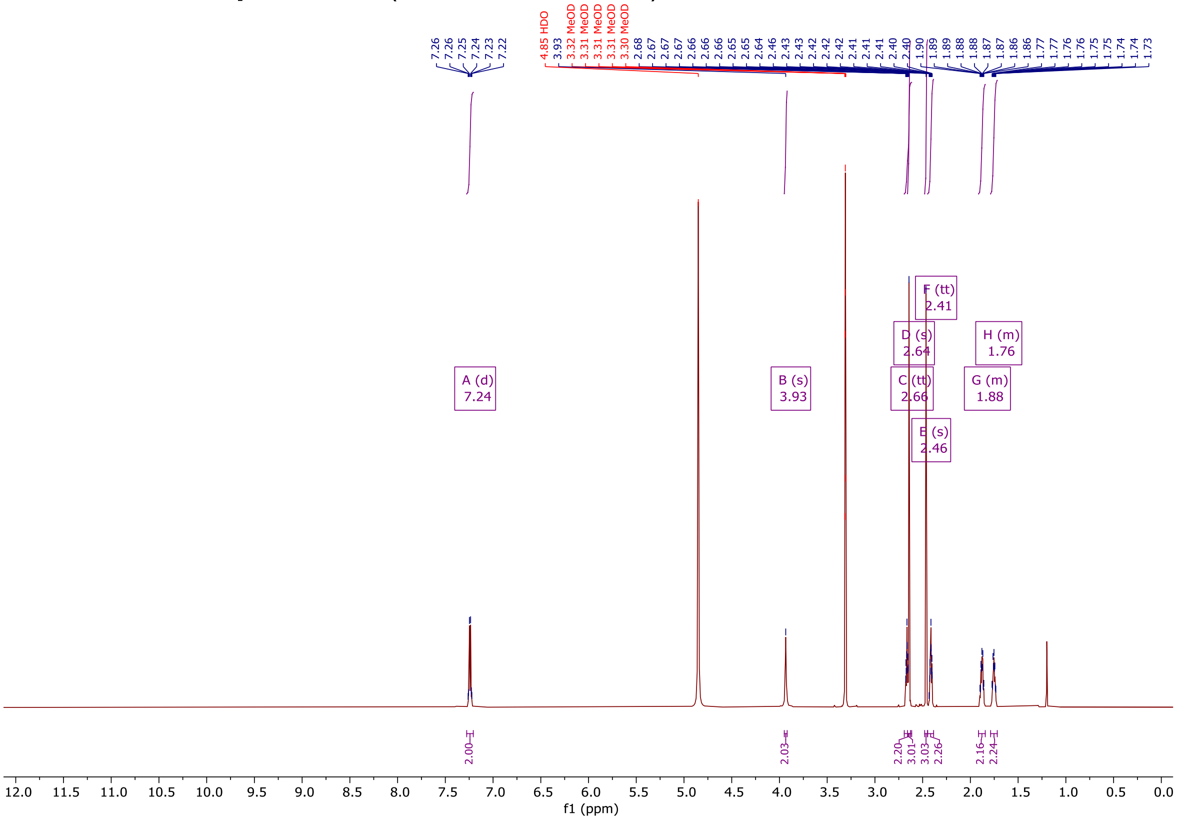

^{13}C NMR of **Compound 23** (150 MHz, CD_3OD)

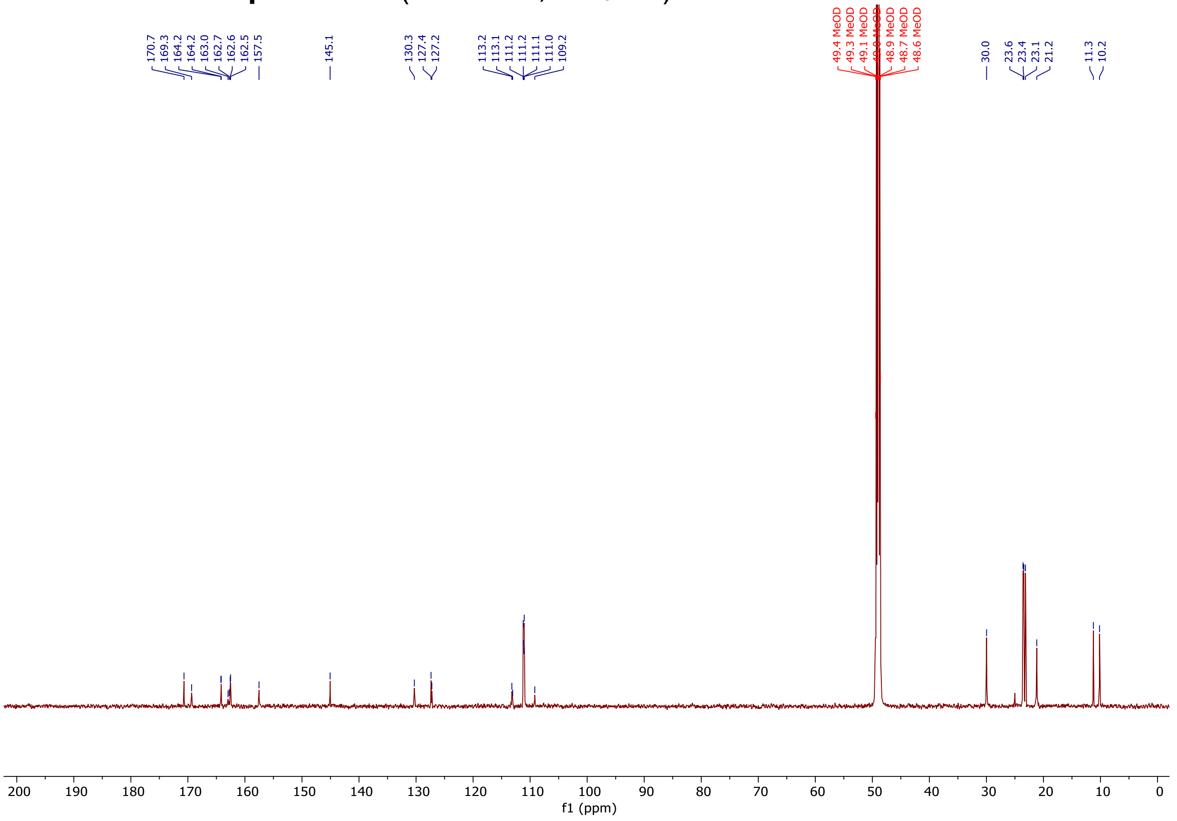

^{1}H NMR of **Compound 20** (600 MHz, CD_3OD)

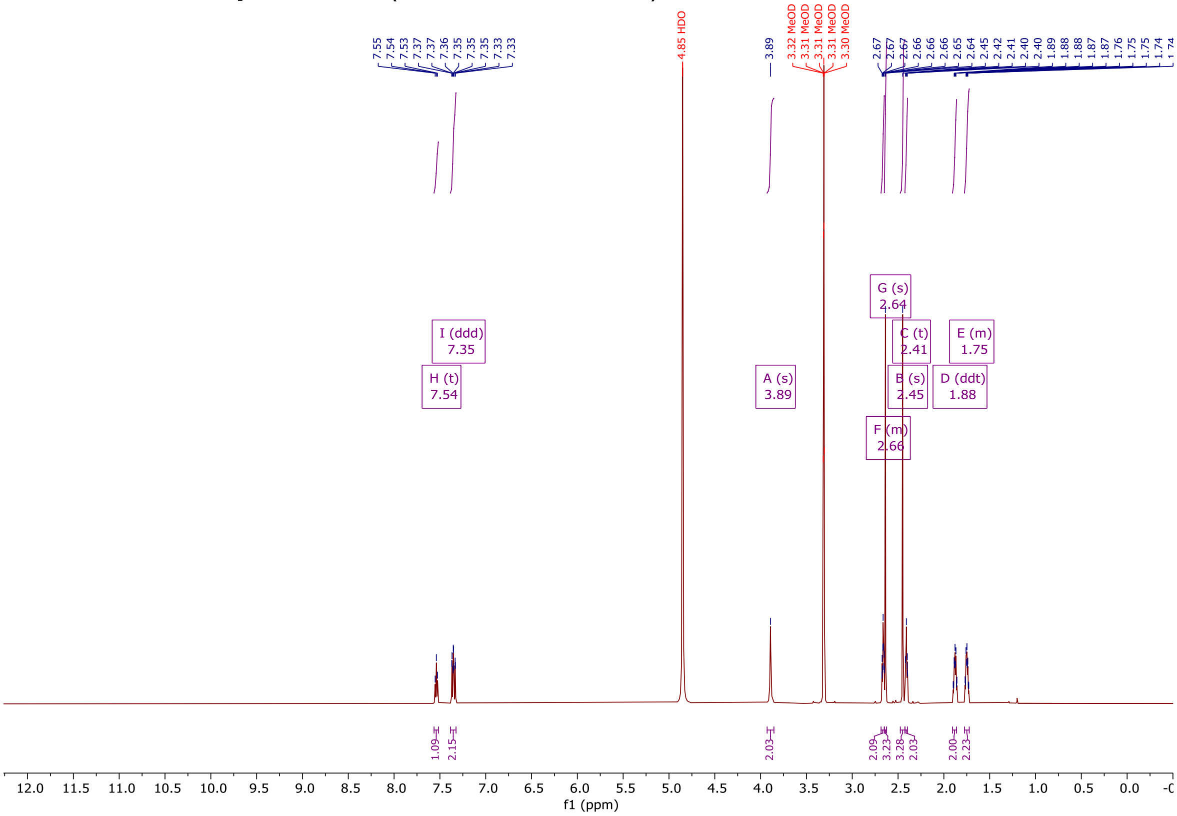

^{13}C NMR of **Compound 20** (150 MHz, CD_3OD)

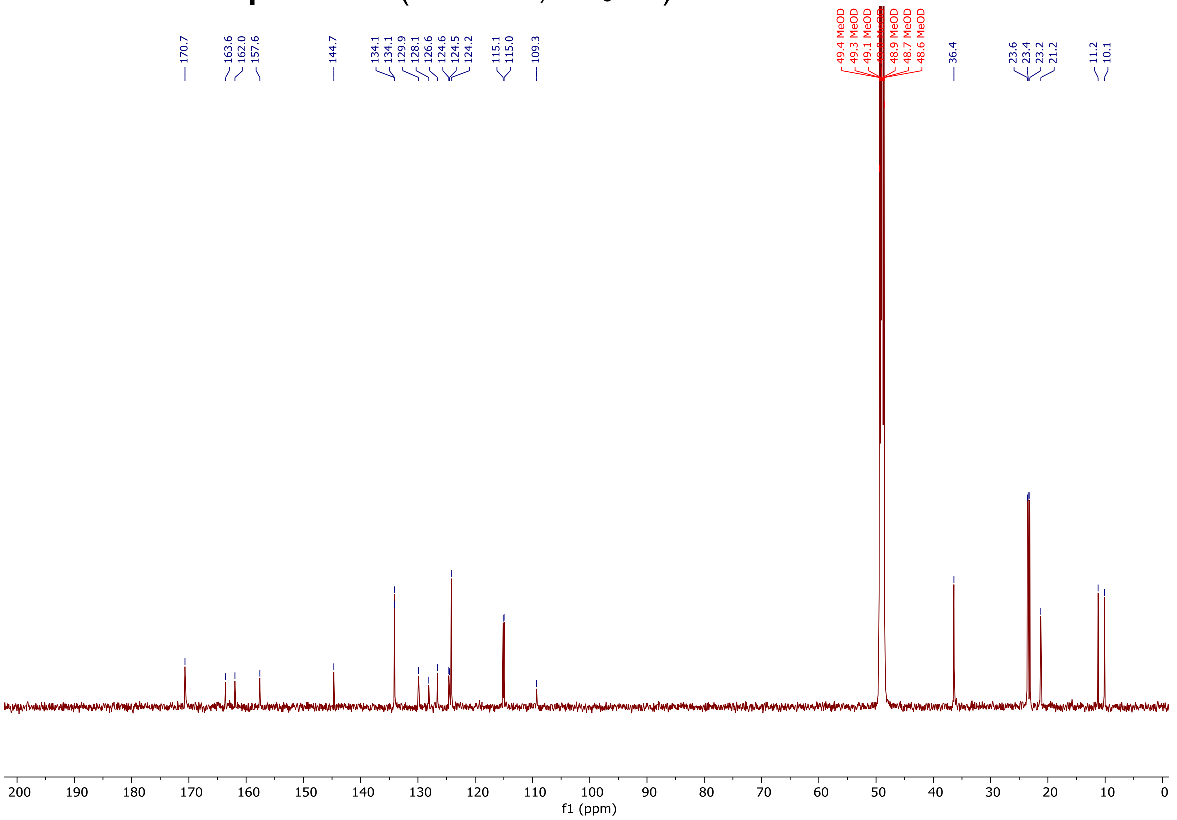

^{1}H NMR of **Compound 19** (600 MHz, CD_3OD)

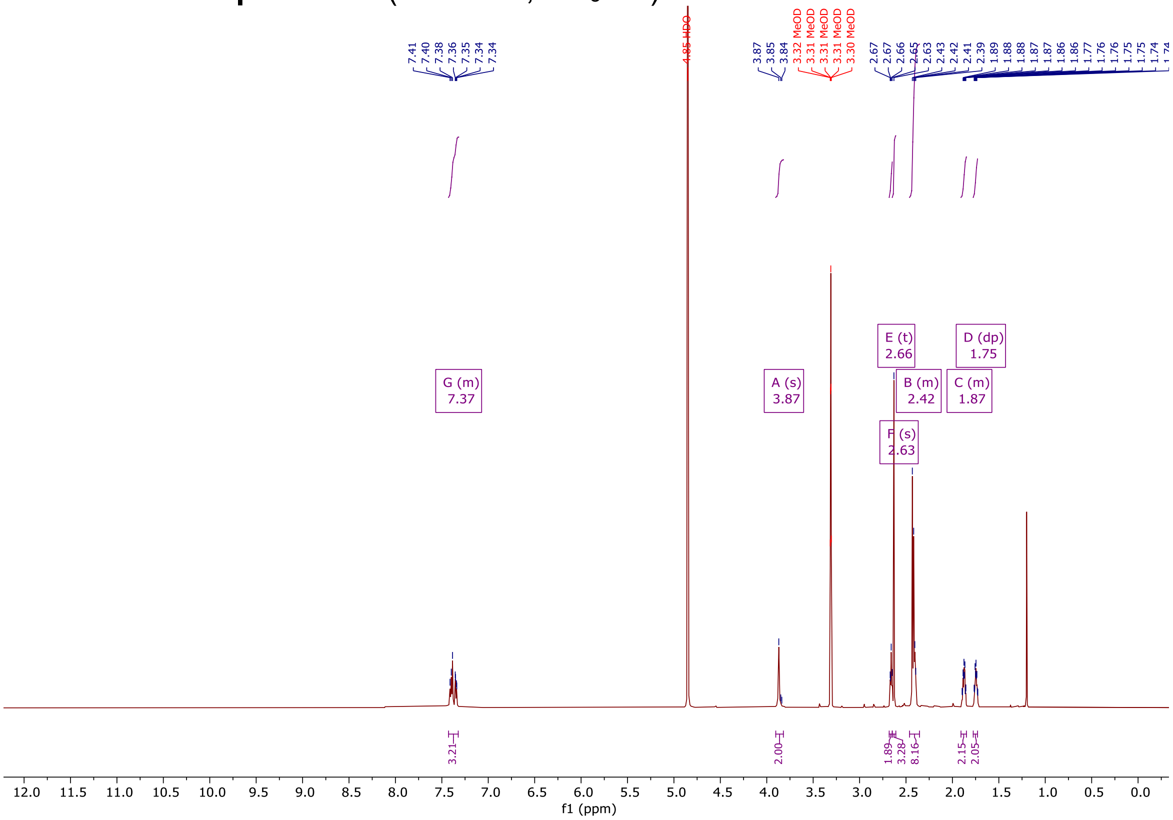

^{13}C NMR of **Compound 19** (150 MHz, CD_3OD)

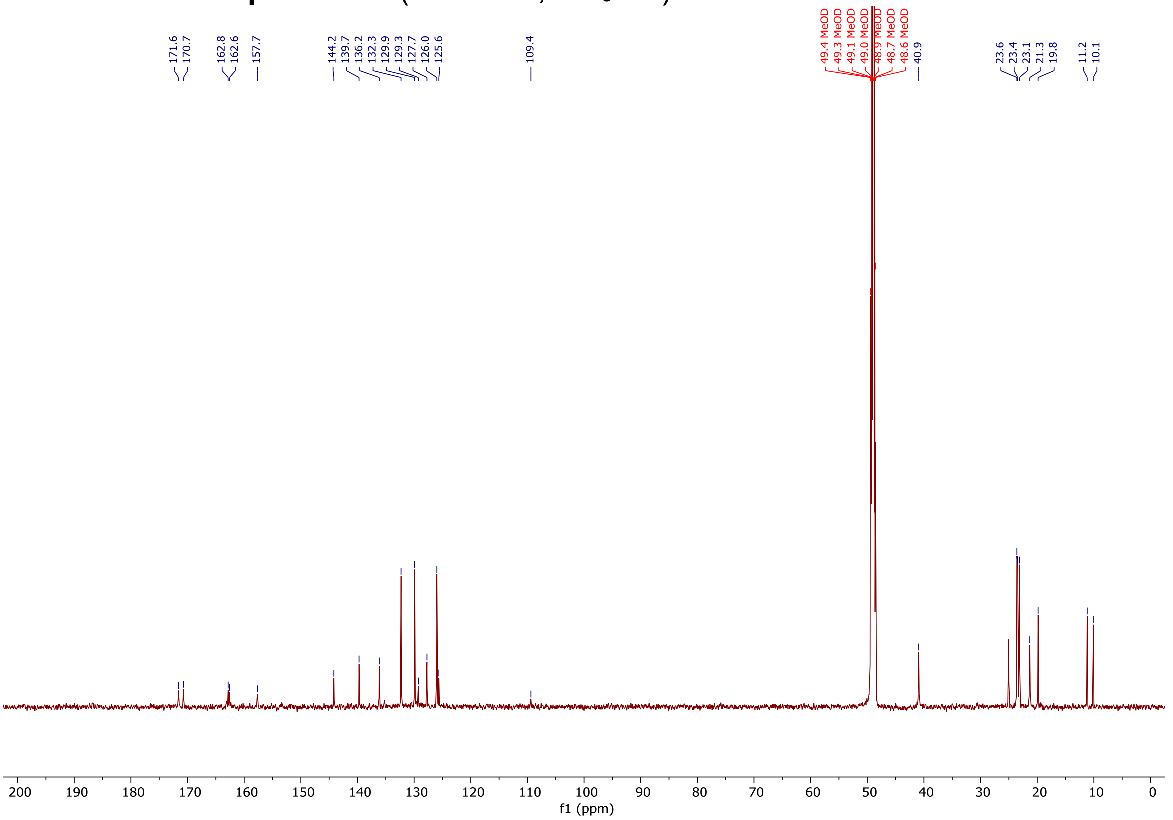

^{1}H NMR of **Compound 17** (500 MHz, CD_3OD)

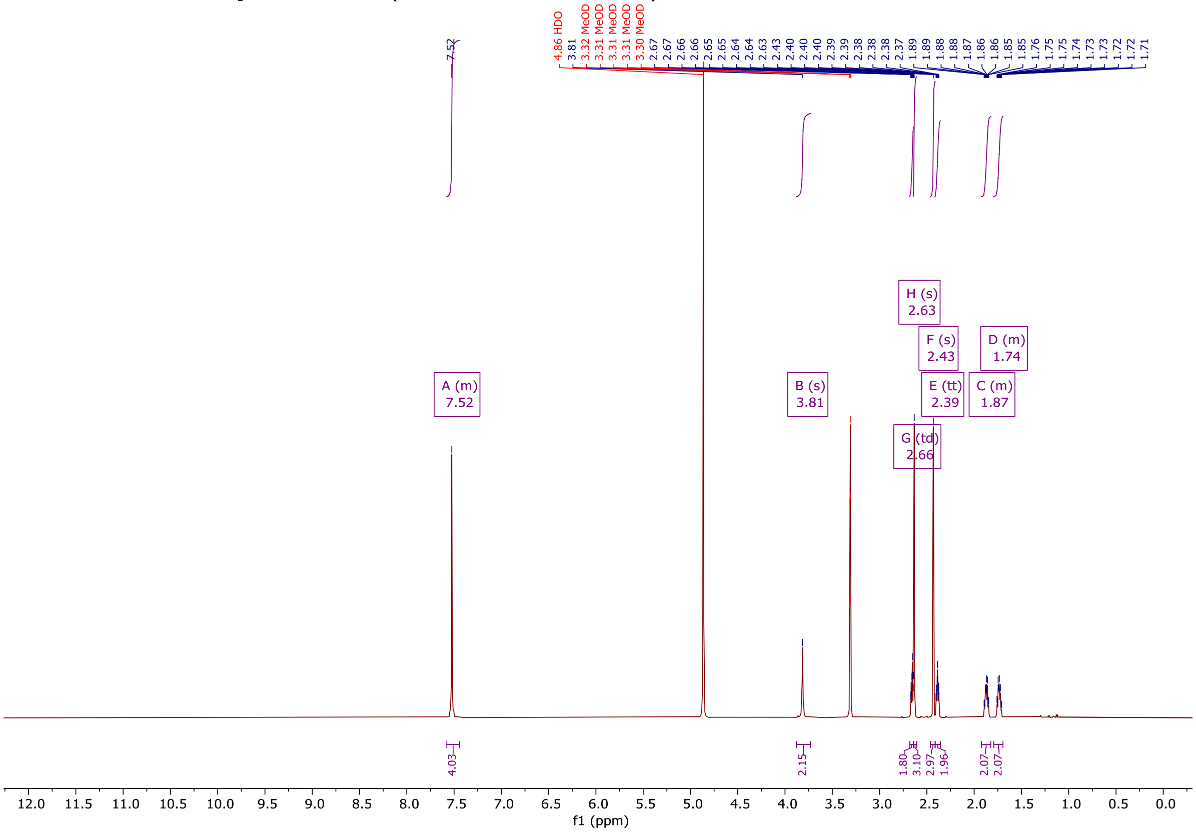

^{13}C NMR of **Compound 17** (125 MHz, CD_3OD)

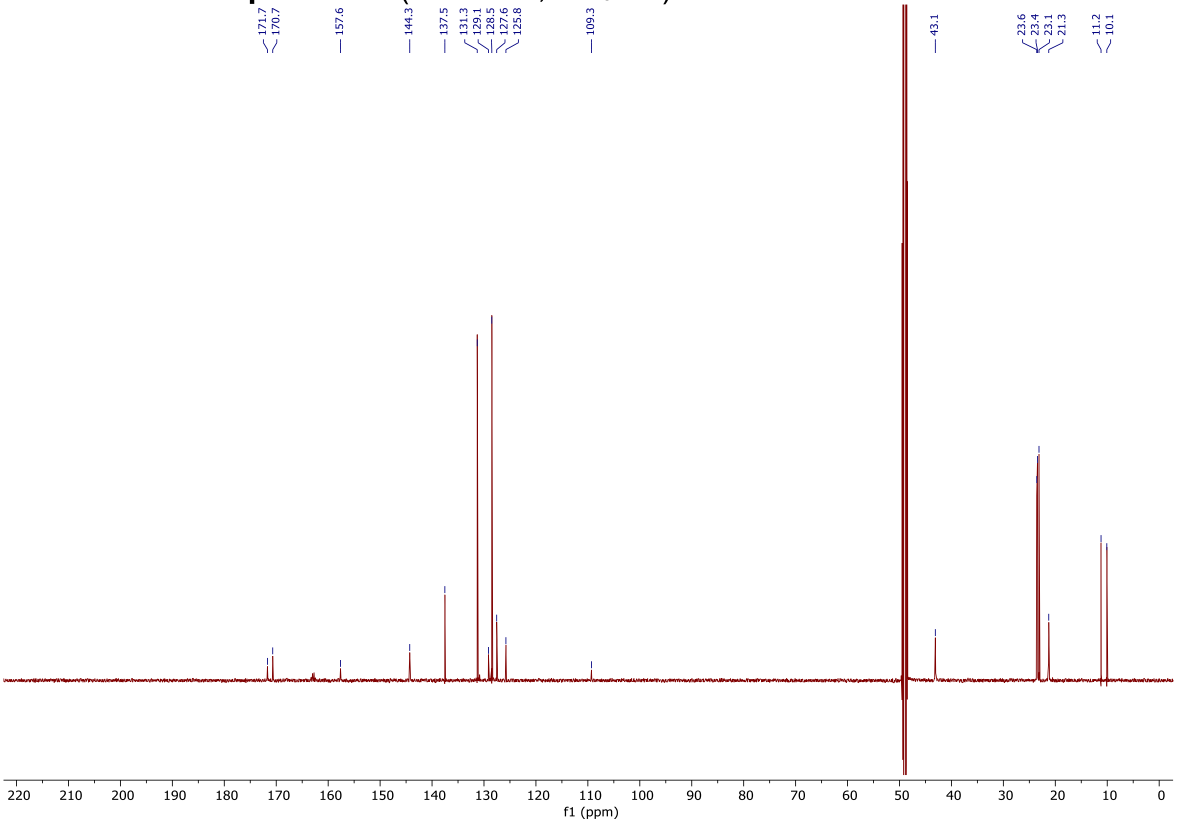

^{1}H NMR of **Compound 9** (500 MHz, CD_3OD)

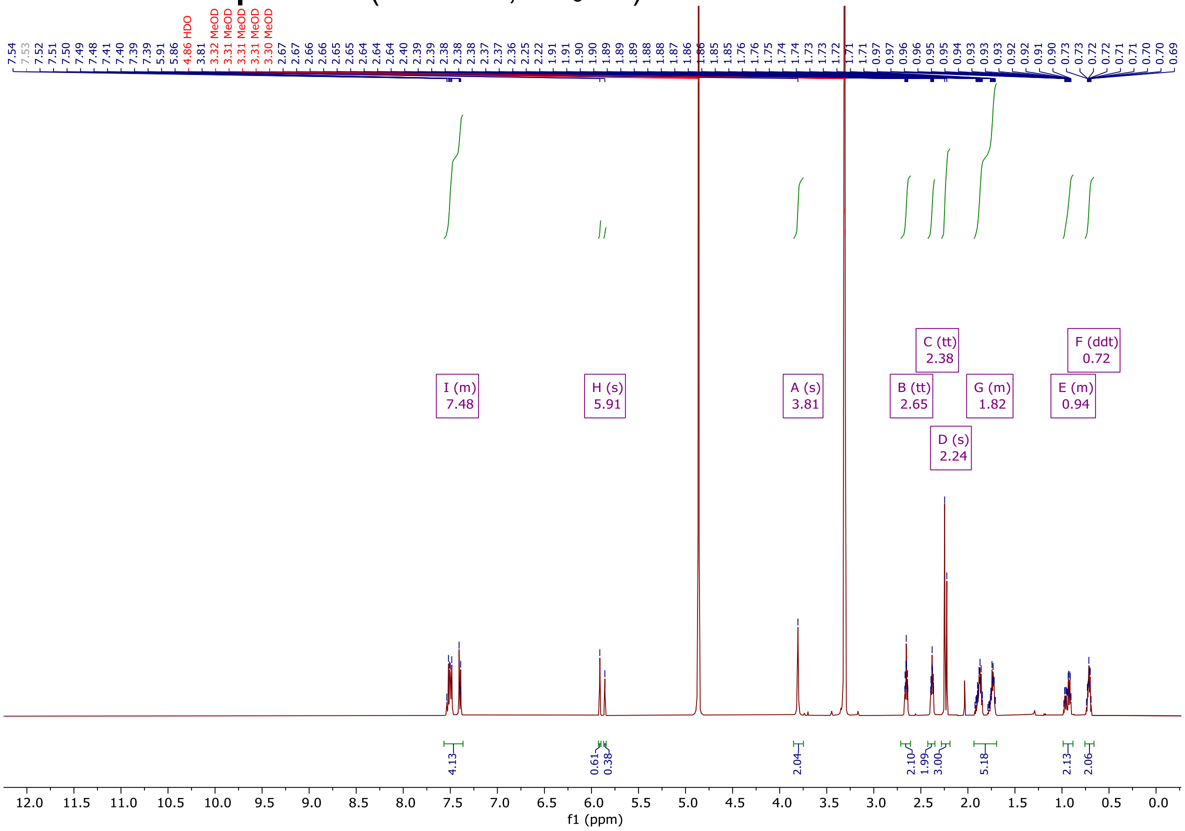

^{13}C NMR of **Compound 9** (125 MHz, CD_3OD)

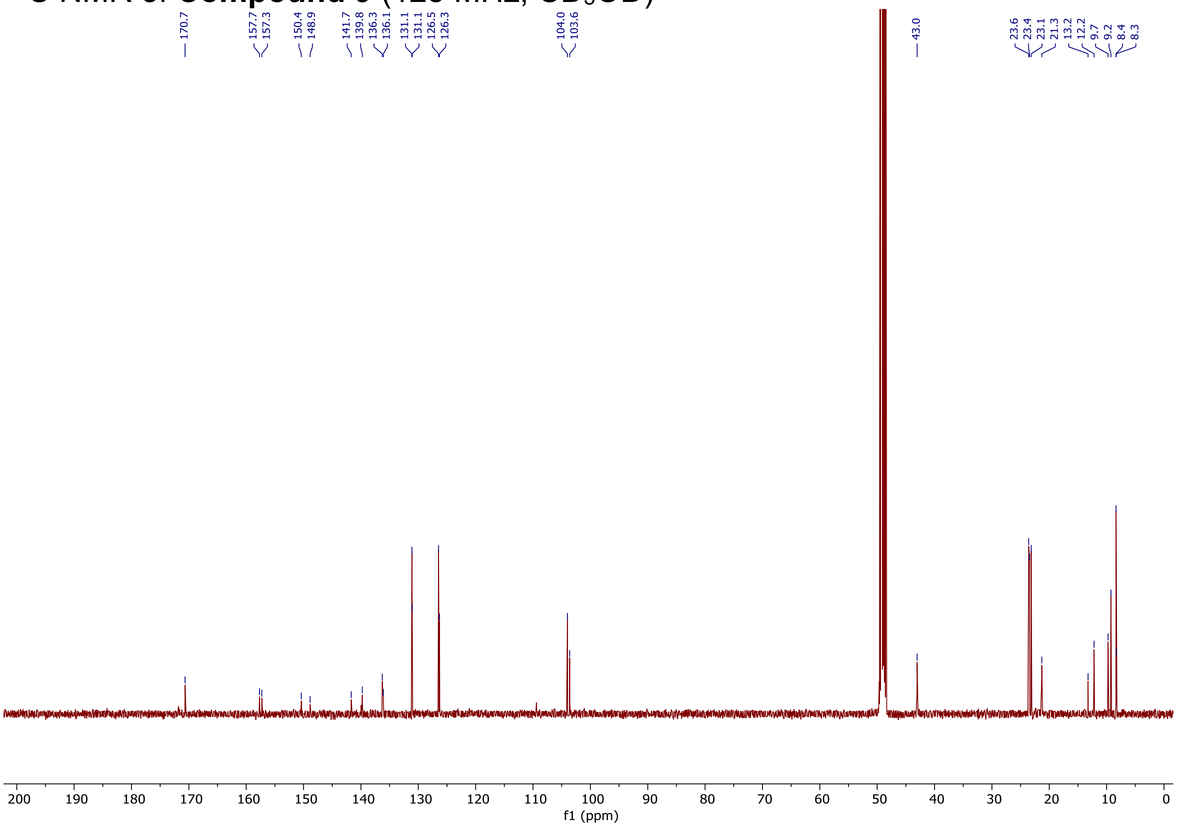

^{1}H NMR of **Compound 8** (600 MHz, CD_3OD)

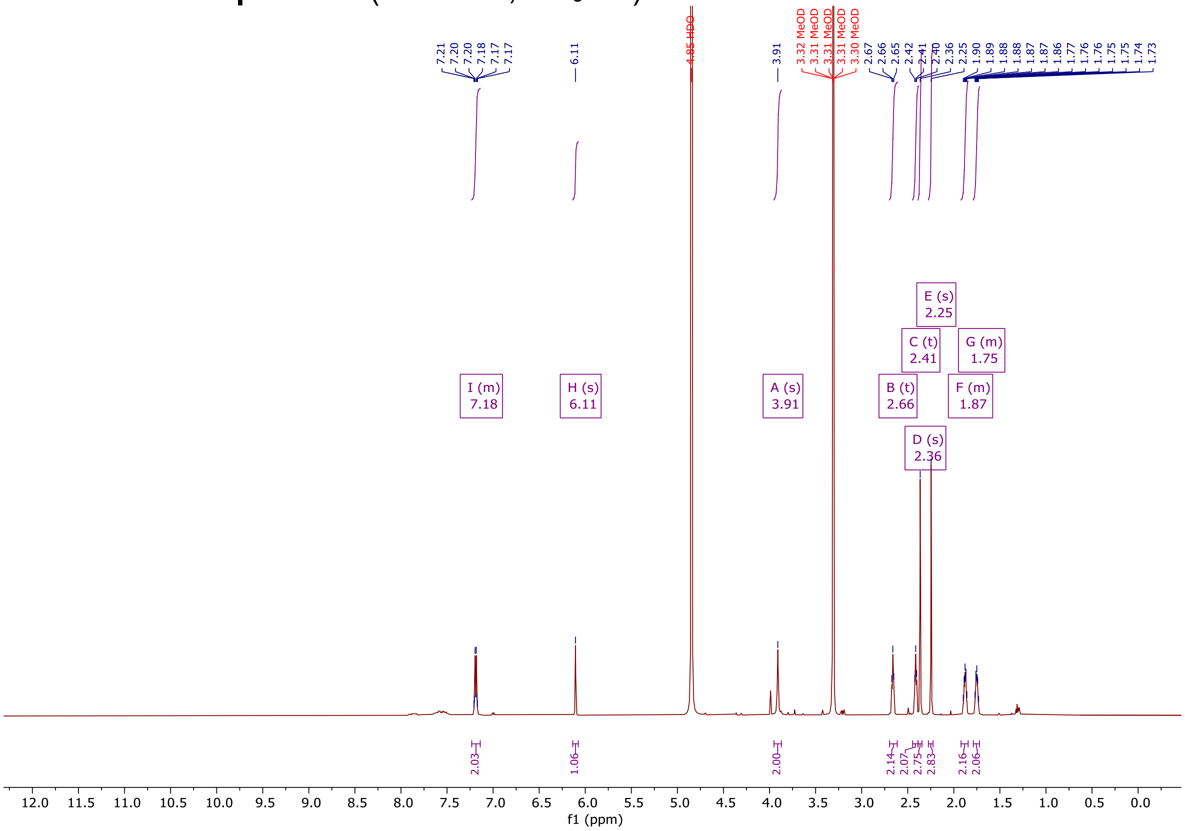

^{13}C NMR of **Compound 8** (150 MHz, CD_3OD)

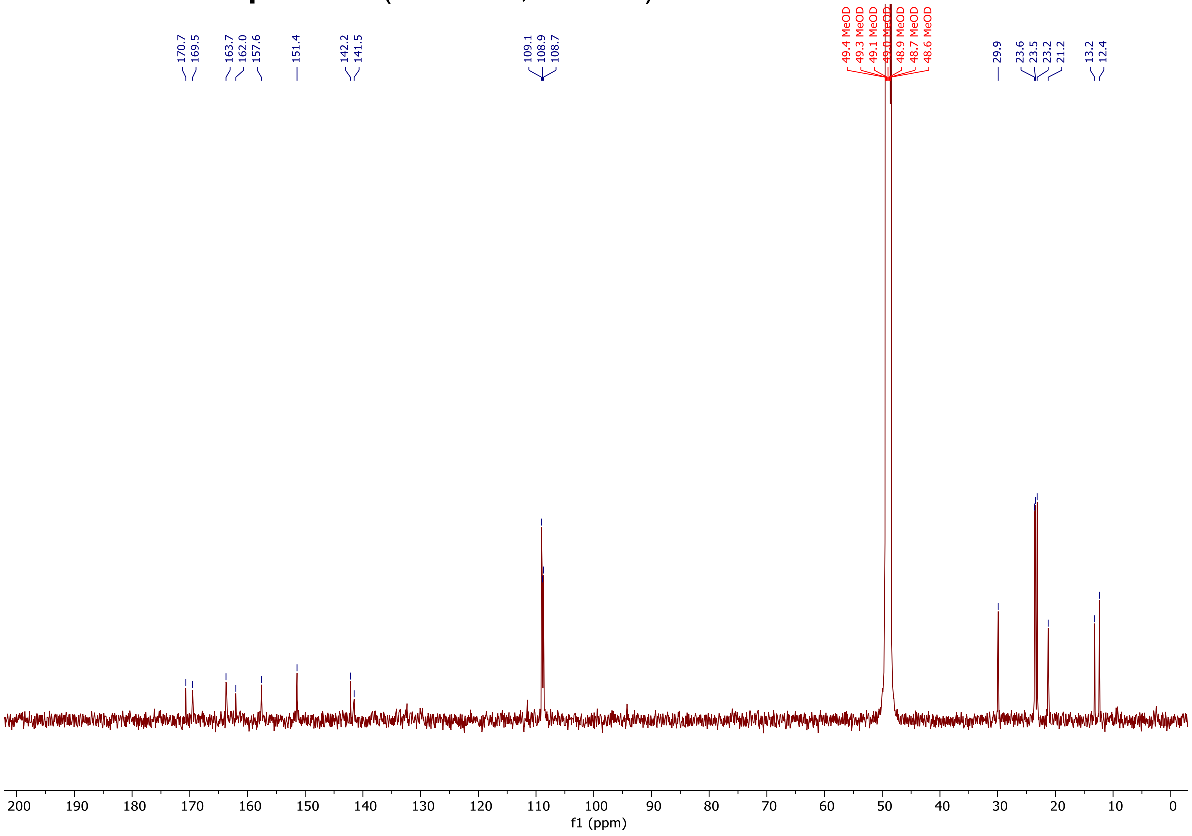

^{1}H NMR of **Compound 10** (500 MHz, CD_3OD)

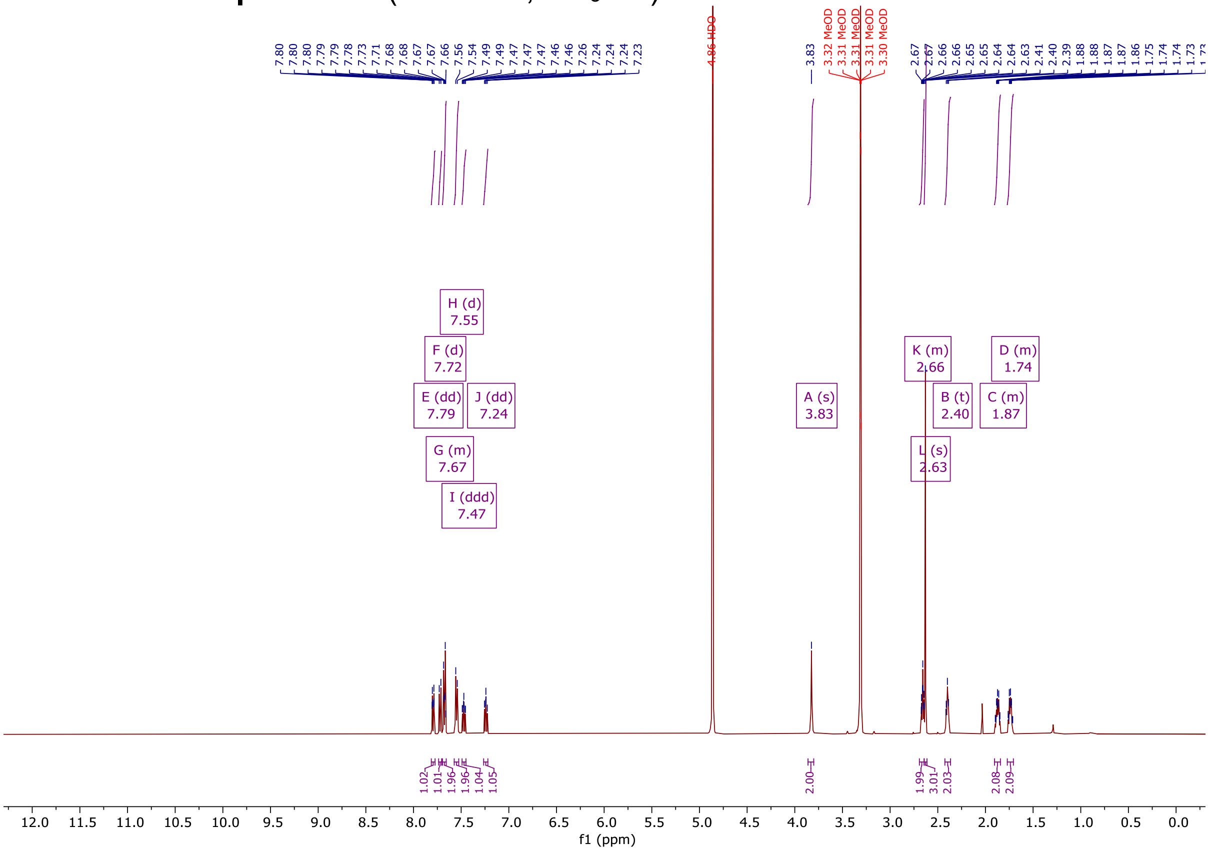

^{13}C NMR of **Compound 10** (125 MHz, CD_3OD)

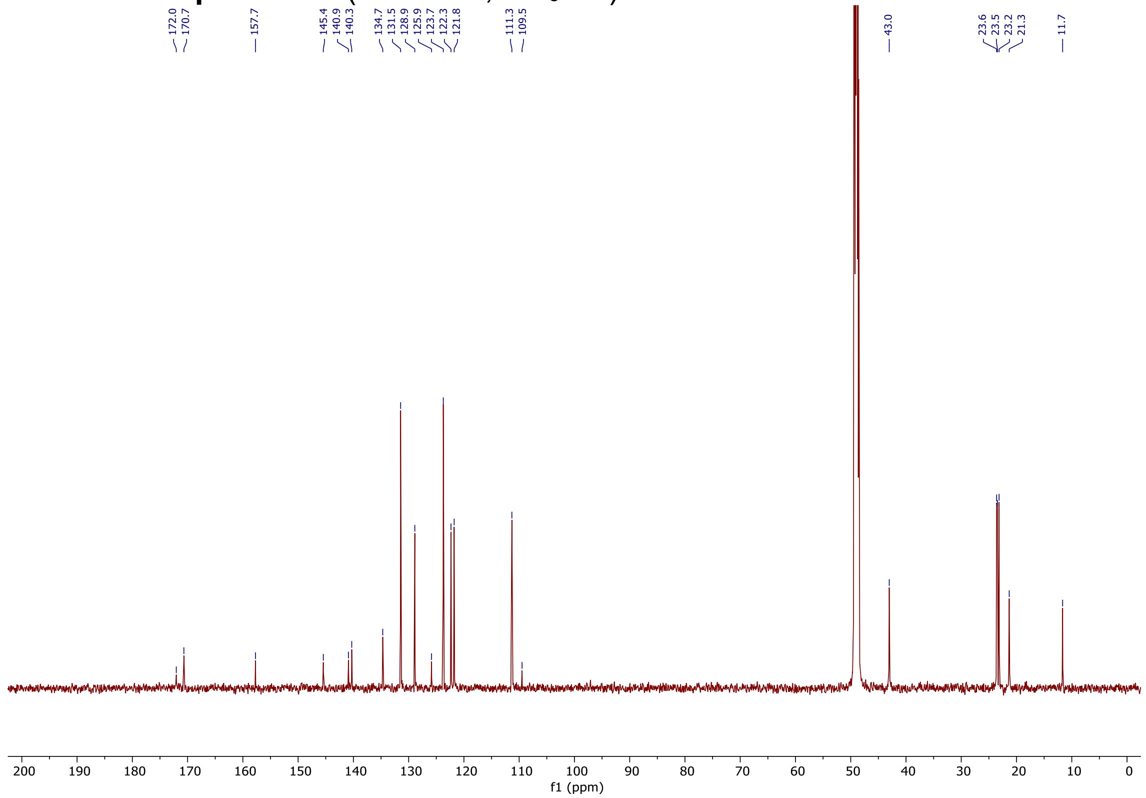

Supplementary references

1. Visnes, T. *et al.* Small-molecule inhibitor of OGG1 suppresses proinflammatory gene expression and inflammation. *Science* **362**, 834-839 (2018).
2. Visnes, T. *et al.* Targeting OGG1 arrests cancer cell proliferation by inducing replication stress. *Nucleic Acids Res*. **48**, 12234-12251 (2020).
3. Mitchell L.H., *et al*. Novel Oxindole Sulfonamides and Sulfamides: EPZ031686, the First Orally Bioavailable Small Molecule SMYD3 Inhibitor. *ACS Med. Chem. Lett.*, **7** 134-138 (2015)
4. Dubianok Y., *et al*., 2018, https://www.rcsb.org/structure/5QJK
5. Grosjean H., *et al*., 2020, https://www.rcsb.org/structure/5RKI
