# Supplementary material for: Virtual fragment screening for DNA repair inhibitors in vast chemical space
Source: Nat Commun. 2025 Feb 18;16:1741. doi: 10.1038/s41467-025-56893-9 (PMC11836371; doi:10.1038/s41467-025-56893-9)
Supplement: Supplementary file 2 — Reporting Summary [file 41467_2025_56893_MOESM2_ESM.pdf]

nature portfolio

Corresponding author(s): Jens Carlsson

Last updated by author(s): Dec 5, 2024

Reporting Summary

Nature Portfolio wishes to improve the reproducibility of the work that we publish. This form provides structure for consistency and transparency in reporting. For further information on Nature Portfolio policies, see our Editorial Policies and the Editorial Policy Checklist.

Statistics

For all statistical analyses, confirm that the following items are present in the figure legend, table legend, main text, or Methods section.

n/a	Confirmed	
☐	☒	The exact sample size (*n*) for each experimental group/condition, given as a discrete number and unit of measurement
☐	☒	A statement on whether measurements were taken from distinct samples or whether the same sample was measured repeatedly
☐	☒	The statistical test(s) used AND whether they are one- or two-sided<br>*Only common tests should be described solely by name; describe more complex techniques in the Methods section.*
☒	☐	A description of all covariates tested
☐	☒	A description of any assumptions or corrections, such as tests of normality and adjustment for multiple comparisons
☐	☒	A full description of the statistical parameters including central tendency (e.g. means) or other basic estimates (e.g. regression coefficient) AND variation (e.g. standard deviation) or associated estimates of uncertainty (e.g. confidence intervals)
☐	☒	For null hypothesis testing, the test statistic (e.g. *F*, *t*, *r*) with confidence intervals, effect sizes, degrees of freedom and *P* value noted<br>*Give P values as exact values whenever suitable.*
☒	☐	For Bayesian analysis, information on the choice of priors and Markov chain Monte Carlo settings
☒	☐	For hierarchical and complex designs, identification of the appropriate level for tests and full reporting of outcomes
☒	☐	Estimates of effect sizes (e.g. Cohen's *d*, Pearson's *r*), indicating how they were calculated

Our web collection on statistics for biologists contains articles on many of the points above.

Software and code

Policy information about availability of computer code

Data collection	* Molecular modeling: DOCK3.7.1<br>* smarts.plus (https://smarts.plus/)<br>* CCP4 suite version 7.1.016 (https://www.ccp4.ac.uk/), containing Phaser version 2.8.3, COOT version 0.9.8.7, REFMAC5 version 5.8.0267<br>* Prism v.6.07 (GraphPad Software)
Data analysis	* Molecular modeling: UCSF Chimera V.1.12.0, interactive SMARTS visualizer (https://smarts.plus), cheminfTools 1.0, RDKit 2019_Q3 and OpenEye Toolkits 2020.0.4.<br>* XLfit version 5.5.0.5 (IDBS)<br>* ImageJ version 1.54f (https://imagej.net/ij/)

For manuscripts utilizing custom algorithms or software that are central to the research but not yet described in published literature, software must be made available to editors and reviewers. We strongly encourage code deposition in a community repository (e.g. GitHub). See the Nature Portfolio guidelines for submitting code & software for further information.

Data

Policy information about availability of data

All manuscripts must include a data availability statement. This statement should provide the following information, where applicable:

- Accession codes, unique identifiers, or web links for publicly available datasets
- A description of any restrictions on data availability
- For clinical datasets or third party data, please ensure that the statement adheres to our policy

* Molecular modeling: The identities of the compounds docked in this study are freely available from the ZINC database (https://zinc15.docking.org) and the Enamine REAL database (https://enamine.net/compound-collections/real-compounds/real-database). Most compounds may be purchased from Enamine, with the exception of compounds 7 - 23, which were synthesized in house. Synthesis routes and NMR/LCMS spectra of compounds 7 - 23 are presented in the supporting information.

* Crystallographic structures and the corresponding structural factors were deposited in Protein Data Bank (https://www.rcsb.org), with the corresponding PDB accession codes: 7QEL, 7ZG3, 8CEX, 8CEY, 7Z5R, 7ZC7, 7Z3Y, 7Z5B. Crystallographic data collection and refinement statistics are presented in supplementary table 3.

* Figures with associated raw data include: figures 1, 2, 3, 4, 5 and supplementary figure S1. Additional data are included in the Source Data file.

* Tables with associated raw data include: table 1 and supplementary tables 1, 2, 4, 5, 6, 7, 8. Additional data are included in the Source Data file.
Table 1: Compound identifier, Lewis structure, OGG1 enzyme inhibition pIC50 ± SD (M), OGG1 thermal stabilization ± SD (K)
Supplementary table 1: Lewis structure, compound identifier, docking score (kcal/mol), OGG1 dock rank, OGG1 cluster rank, OGG1 thermal stabilization (K)
Supplementary table 2: Virtual fragment screening hits
Supplementary table 3: Data collection and refinement statistics from crystallographic experiments
Supplementary table 4: Lewis structure, compound identifier, OGG1 enzyme inhibition IC50 (uM)
Supplementary table 5: Compound identifier, Lewis structure, OGG1 enzyme inhibition pIC50 ± SD (M), NFkB cellular efficacy pEC50 ± SD (M), OGG1 thermal stabilization ± SD (K)
Supplementary table 6: Physicochemical and in vitro ADME properties of OGG1 inhibitors
Supplementary table 7: Comparison of chemical space sizes

Source data are provided with this paper.

Research involving human participants, their data, or biological material

Policy information about studies with human participants or human data. See also policy information about sex, gender (identity/presentation), and sexual orientation and race, ethnicity and racism.

Reporting on sex and gender	N/A
Reporting on race, ethnicity, or other socially relevant groupings	N/A
Population characteristics	N/A
Recruitment	N/A
Ethics oversight	N/A

Note that full information on the approval of the study protocol must also be provided in the manuscript.

Field-specific reporting

Please select the one below that is the best fit for your research. If you are not sure, read the appropriate sections before making your selection.

[x] Life sciences [ ] Behavioural & social sciences [ ] Ecological, evolutionary & environmental sciences

For a reference copy of the document with all sections, see nature.com/documents/nr-reporting-summary-flat.pdf

Life sciences study design

All studies must disclose on these points even when the disclosure is negative.

Sample size	Unless otherwise stated, n=3 was chosen as the minimal number of replicates per experiment for advanced molecules that would allow for adequate analysis to draw statistically meaningful conclusions from the data. We determined this to be sufficient based on the low observed variability between samples from the experiments.
Data exclusions	No data was excluded from analysis.

Replication	All attempts at replication have been successful. To ensure reproducibility, the same reagent suppliers and catalogue numbers were maintained as long as possible at all concerned experimental locations. When reagents needed to be replenished, validation and optimization experiments were conducted to ensure data reproducibility.<br><br>Figure 2: f; Mean values ± SD from three independent experiments.<br>Figure 3: b; IC50 ± SD (uM) from three independent experiments., c; relative stabilization ± SEM from three independent experiments. d; Mean maximum intensity from three independent experiments., e; Mean values of 15 cells for each condition from three independent experiments are shown (5 cells per experiment). Relative intensity ± SEM from three independent experiments. f; Percentage cellular efficacy ± SD from three (compound 23) or six (TH5487) independent experiments. g; percentage cell viability from two independent experiments. h; cytotoxicity from two independent experiments.<br><br>Table 1: OGG1 enzyme inhibition pIC50 ± SD (M) from three independent experiments. Thermal stabilization ± SD (K) from three independent experiments.<br>Supplementary table 1: OGG1 thermal stabilization (K) from four technical replicates<br>Supplementary table 2: OGG1 enzyme inhibition IC50 (uM) was carried out in two independent experiments, OGG1 thermal stabilization (K) was carried out in four technical replicates.<br>Supplementary table 4: OGG1 enzyme inhibition (uM), n=1-3 independent experiments<br>Supplementary table 5: OGG1 enzyme inhibition pIC50 ± SD (M, n=1-5 independent experiments), NFkB cellular efficacy pEC50 ± SD (M, n=1-3 independent experiments), OGG1 thermal stabilization (K, n=1-4 independent experiments)<br>Supplementary table 6: in vitro ADME properties determined from a single replicate experiment
Randomization	Not applicable.
Blinding	Not applicable.

Reporting for specific materials, systems and methods

We require information from authors about some types of materials, experimental systems and methods used in many studies. Here, indicate whether each material, system or method listed is relevant to your study. If you are not sure if a list item applies to your research, read the appropriate section before selecting a response.

Materials & experimental systems

n/a	Involved in the study	
☐	☒	Antibodies
☐	☒	Eukaryotic cell lines
☒	☐	Palaeontology and archaeology
☒	☐	Animals and other organisms
☒	☐	Clinical data
☒	☐	Dual use research of concern
☒	☐	Plants

Methods

n/a	Involved in the study	
☒	☐	ChIP-seq
☒	☐	Flow cytometry
☒	☐	MRI-based neuroimaging

Antibodies

Antibodies used	Anti-β-Actin (Abcam, Cat. #ab6276, 1:10 000); Anti-SOD1 (G-11) (Santa Cruz Biotechnology, Cat. #sc-17767, 1:1000); Anti-OGG1 (Abcam, Cat. #ab124741, 1:1000).
Validation	All antibodies have been validated for their use in their respective application including western blotting as stated on the manufacturers' product pages.<br>Anti-β-Actin: KO validated. Suitable for ICC/IF, WB. Reacts with Mouse, Rat, Cow, Dog, Human, African green monkey, Chinese hamster. HeLa, Jurkat, COS-7, NIH/3T3, PC-12, Rat2, CHO, MDBK, and MDCK (Dox-inducible β-Actin) whole cell lysates were used as positive controls.<br>Anti-SOD1 (G-11): Suitable for WB, IP, IF, IHC-P, ELISA. Reacts with Human. Jurkat, DU 145 and HEK293T whole cell lysates were used as positive controls.<br>Anti-OGG1: Suitable for WB. Reacts with Human. In house validation for WB using OGG1 KO U2OS cells, siRNA and shRNA in several human cell lines.

Eukaryotic cell lines

Policy information about cell lines and Sex and Gender in Research

Cell line source(s)	A2780: ECACC 93112519, female<br>A549: ATCC 60150896, male<br>HCT116: ATCC CCL-247, male<br>BJhTERT: Gift from William Hahn, male<br>HEK293T pGF NFkB: female

	U2OS: ECACC 92022711, female<br>HL60: ATCC, CCL-240, female
Authentication	A2780, HCT116 and HEK293T cell lines were authenticated with STR profiling, frozen down and these stocks were used in experiments.
Mycoplasma contamination	All cell lines were routinely tested for mycoplasma contamination using the MycoAlertTM Mycoplasma Detection Kit (Lonza). All cell lines used in this study tested negative for mycoplasma contamination
Commonly misidentified lines<br>(See ICLAC register)	None of the cell lines used in this study are listed in the ILAC register of commonly misidentified cell lines.

Plants

Seed stocks	Not applicable.
Novel plant genotypes	Not applicable.
Authentication	Not applicable.
